# Conceptualising hardship areas in Sub-Saharan Africa: a scoping review

**DOI:** 10.1186/s12939-025-02694-x

**Published:** 2025-11-21

**Authors:** Caroline M. N. Auma, Pauline Karing’u, Eli Harriss, Mike English, Jacquie Oliwa, Emelda A. Okiro

**Affiliations:** 1 Population and Health Impact Surveillance Group, KEMRI-Wellcome Trust Research Programme, Nairobi, Kenya; 2 Health Services Unit, KEMRI-Wellcome Trust Research Programme, Nairobi, Kenya; 3 Department of Public Health, Institute of Tropical Medicine, Antwerp, Belgium; 4 Bodleian Health Care Libraries, University of Oxford, Oxford, UK; 5 Centre for Tropical Medicine and Global Health, Nuffield Department of Clinical Medicine, University of Oxford, Oxford, UK

**Keywords:** Hardship areas, Sub-Saharan Africa, Hard-to-reach, Remote, Underserved, Hard-to-stay, Deprived areas, Difficult regions, Skilled workforce

## Abstract

**Introduction:**

Many national development strategies are implemented at the subnational administrative level, serving as critical units for service delivery. Some subnational levels remain underserved and face significant obstacles to achieving equitable development. In Sub-Saharan Africa, underserved regions are often called hardship areas; however, there is no clarity on how such areas are defined across various contexts. Therefore, this scoping review aimed to delineate the definitions of hardship areas across countries in Sub-Saharan Africa and develop a unified typology of their features.

**Methods:**

This scoping review followed the framework by Arksey and O’Malley, aligned with Preferred Reporting Items for Systematic Reviews and Meta-Analyses extension for Scoping Reviews. We searched Ovid Embase, Ovid MEDLINE, CINAHL via EBSCOhost, Ovid Global Health, and Scopus from 1st January 2010 to 8th October 2024, as well as grey literature from websites. We imported the retrieved articles into Covidence. One reviewer conducted the title and abstract screening. For full-text review, one reviewer assessed all the articles, whereas the second reviewer independently evaluated a random sample of 20%, achieving a 90% agreement. Data were systematically extracted, tabulated, and synthesised using deductive and inductive thematic approaches.

**Results:**

Of the 15,017 articles screened, 210 articles met the inclusion criteria. Only 137 articles were included, as the remaining 73 provided no new information. We identified several definitions and synthesised them into 79 relatively distinct features. These were grouped into 6 contextual themes: physical/geographical location and accessibility, social services, economic and livelihood challenges, demographic characteristics, security and governance, and natural and man-made disasters, with 16 subthemes.

**Strengths and limitations:**

We applied the Arksey and O’Malley framework, which fostered collaborative engagement in developing the research topic and search strategy. Limitations included few primary studies capturing lived experiences, a focus on three AFRHiCARE countries which restricted broader policy comparisons, and omission of non-English publications.

**Conclusion:**

In Sub-Saharan Africa, hardship is a multidimensional notion with context-dependent features. The typology developed in this review lays the groundwork for geospatial characterisation of hardship, bridges definitional gaps, and supports transparent, effective, and equitable policy responses.

**Supplementary Information:**

The online version contains supplementary material available at 10.1186/s12939-025-02694-x.

## Introduction

The concept of equitable development emphasises that all people, regardless of geography or context, should have access to essential services and opportunities for improved well-being [1]. Many national development strategies, such as health, education, and infrastructure, are implemented through the subnational administrative level to foster inclusive growth [2]. In decentralised systems, it is imperative to comprehend and address the challenges facing these levels. Doing so is critical to achieving national and global goals, including the Sustainable Development Goals (SDGs), which call for leaving no one behind [3]. However, some subnational administrative levels remain underserved and face significant obstacles to equitable development and access to critical services.

Underserved subnational administrative levels are often referred to as hardship areas. They include arid and semi-arid areas, conflict and post-conflict zones, remote and rural areas, small or remote islands. Other examples are urban slums, refugee camps, minority and indigenous communities, and areas severely affected by natural or man-made disasters [4]. They are often defined by insecurity, limited infrastructure, harsh and severe climatic conditions, as well as inadequate access to essential services [5].

These definitions are not merely descriptive; they shape how governments identify hard to staff regions and rationalise the introduction of workforce recruitment and retention policies. For instance, the WHO emphasise that defining hardship areas is essential for formulating targeted workforce retention strategies [4]. The challenges in hardship areas hinder the delivery of services, constrain local development, and create formidable obstacles to attracting and retaining skilled professionals. In response, governments have introduced recruitment and retention strategies, including financial and non-financial incentives [6–9]. While such strategies are important, it is also necessary to ameliorate the conditions that characterise hardship areas, thereby enhancing their appeal as viable and supportive workplaces for skilled workers.

Nearly all African countries have areas that are hard to staff [10]. However, there is a lack of consensus on what features define hardship or hard-to-staff areas. Inconsistent definitions deter comparative assessment of strategies for recruiting and retaining skilled workers and undermine the use of evidence in policy and planning. Without clear and consistent definitions of hardship features, our understanding of intervention effectiveness remains fragmented.

The consequences of hardship are perhaps most visible in the health sector, where staffing inadequacies can hinder access to essential services for significant portions of the population and jeopardise progress towards Universal Health Coverage (UHC) and SDGs [4]. The staffing challenges span both retention and recruitment [11, 12]. Despite hiring a considerable number of skilled workers, many do not stay for long, resulting in high turnover rates [8]. Persistent retention challenges call for a deeper understanding of the characteristics that define hardship areas to inform effective solutions.

Consequently, this scoping review sought to examine definitions of hardship areas in Sub-Saharan Africa (SSA) and propose a more standard typology based on these distinct characteristics. A systematically developed typology, especially one that enables use of modern geospatial techniques to characterise locations, could support policy coherence and cross-country learning, ultimately helping to address inequities.

## Methods

This scoping review adopted the five steps of the Arksey and O’Malley Framework [13] to identify the features of hardship areas: (i) identifying the research question; (ii) identifying relevant studies; (iii) study selection; (iv) data charting; and (v) collating, summarizing, and reporting findings [13]. Reporting the results followed the Preferred Reporting Items for Systematic Reviews and Meta-Analyses extension for Scoping Reviews (PRISMA-ScR) guidelines [14] “see Supplementary Table 1, Additional file I”. The protocol for this review is available in the Open Science Framework (OSF) https://osf.io/tyezj with deviations from the protocol listed in Supplementary Table 2, Additional File 2.

### Identifying the research question

To guide the search strategy, our research question was, “What are the defining characteristics and regional variations of hardship areas that are reported to influence area-specific recruitment and retention of skilled workers in SSA?”

### Eligibility criteria

We selected studies using the population, concept, and context framework as detailed below:

Population: We included studies with participants from human populations, including rural communities, vulnerable, marginalised, displaced people, internal migrants, refugees, and asylum seekers.

Concept: We included studies defining synonyms of hardship areas such as “hard to reach,” “remote,” “rural,” “underserved,” or similar descriptors.

Context/setting: We included studies conducted in Sub-Saharan Africa. 

### Type of studies

We included primary research papers regardless of the methodology, relevant policies, guidelines, and reports from government and non-governmental organisations.

### Identifying relevant studies

The following databases were searched on 08/10/2024 to retrieve relevant records about hardship areas in Sub-Saharan Africa: Ovid Embase, Ovid MEDLINE, Ovid Global Health, Scopus, and CINAHL via EBSCOhost. To increase the specificity of our screening, we added a third concept, “health,” to Scopus and Embase. The search results were restricted to English-language publications published between 2010 and the date of the search. The search string can be found in Additional File 3. All references were exported to EndNote 21 (Thomson Reuters, New York, NY) and were deduplicated using a method described by Falconer [15] before being uploaded to Covidence www.covidence.org to complete the deduplication process before screening.

We also searched organisational websites including those of World Bank, World Health Organization (WHO), United Nations, and government websites for Kenya, Uganda, and South Africa. The goal was to supplement scholarly literature with grey literature and policy documents. We chose Kenya, Uganda, and South Africa because the three countries are partners in the AFRHiCARE project. This project aims to strengthen First Referral Hospitals to provide quality essential healthcare in the context of UHC. This focus enabled us to align the review with project objectives and ensured an in-depth analysis in settings directly relevant to the partnership. We also searched the reference lists of included studies using synonyms for hardship, which often contain definitions of hardship areas not captured in academic databases.

### Study selection

One reviewer (CM) evaluated titles and abstracts to determine their relevance in accordance with the inclusion and exclusion criteria. Any uncertainties were clarified through discussions with co-authors. CM assessed all full texts as the primary reviewer, while PK independently evaluated a random sample of 20%, yielding a 90% inter-reviewer agreement rate. Eligible full-text articles were subsequently included for data extraction. Although one reviewer conducted full-text screening introducing a potential risk of bias, this approach was necessary given the resource constraints. Importantly, our approach aligns with guidance from Mak & Thomas [16], which recommends that if dual review is infeasible, one reviewer can lead the review while the second reviewer assesses a subset of the studies to ensure consistency. By ensuring that at least 20% of articles were reviewed by two reviewers, with a minimum agreement rate of 90%, we maintained methodological rigor while balancing feasibility.

### Data charting and collation

We conducted data extraction using a template piloted with 10% of the included studies. After piloting, the research team held a meeting to gather feedback and discuss the results. Following the discussions, we updated the form embedded in Covidence and then proceeded with data extraction. CM led the data extraction process, whereas PK extracted 20% of the studies. Any disagreement was resolved through discussion, until a consensus was reached. Co-investigators EAO, JO, and ME validated the extracted data.

We charted the following items:

general study details, including lead author, year of publication, type of publication, study setting (national and sub-national), and study objectives. We also extracted the definitions of the hardship areas referenced in the included studies: primarily verbatim but edited where needed while preserving the author’s intent.

### Summarizing and reporting findings

We used “hardship” as an umbrella concept that captured synonymous terms, and their characteristics. These characteristics, extracted from the literature, were synthesized into thematic categories using deductive and inductive approaches to illustrate the different dimensions of hardship across contexts.

In the deductive approach, we identified two key references: (1) multisectoral criteria for defining underserved areas in Tanzania [17] and (2) criteria for defining hard-to-reach and stay areas in Uganda [18]. These two papers were chosen because the Tanzania paper took a multisectoral view of geography, economy, and infrastructure in defining hardship areas, while the Uganda report provided a practical example of how a country defines “hard-to-reach and stay” areas, offering a real-world application that could inform a broader framework. The two references informed the initial five themes: (i) geographic and environmental features; (ii) infrastructure and accessibility; (iii) social services and amenities; (iv) socioeconomic and livelihood factors; and (v) security and governance.

During the review of the included studies, we applied an inductive approach to identify additional themes and refine the initial ones. Four new themes emerged: (i) health and well-being factors; (ii) human resources for health; (iii) food insecurity; and (iv) demographic characteristics. We also separated the initial geographic and environmental theme into three: (i) geographical/physical factors, (ii) climate factors, and (iii) environmental challenges. Similarly, we disaggregated the security and governance theme into two components: (i) security and (ii) conflict.

The entire coding and synthesis process yielded an expanded typology of 12 contextual themes. We refined and merged closely correlated themes and created hierarchies to reduce redundancy and improve conceptual clarity. This ultimately condensed the 12 into six higher- order themes, each with two or three subthemes, resulting in a total of 16 subthemes “See Supplementary Fig. 1, Additional File 4”.

The iterative process of creating themes and sub-themes was guided by the identification of relatively distinct features, which served as the most granular elements in defining hardship. Accordingly, the 16 subthemes represent groups of related features connected by the same conceptual link. Conceptual links between sub-themes enabled us to create six higher-order contextual themes (which are broad categories that define or structure the experience of hardship). Thus, each specific feature of hardship identified is nested within a subtheme and, in turn, within a broader contextual theme. In this process, one reviewer (CM) identified the features and proposed initial sub-themes, while co-investigators EAO, JO, and ME refined and validated the sub-themes and higher-order contextual themes.

We present the findings through a combination of tables, maps, word clouds, and Sankey diagrams to illustrate patterns, relationships, and distributional trends in the data.

### Quality assessment

In line with established methodological guidance for scoping reviews [19], we did not conduct a formal quality assessment or critical appraisal of individual studies. Scoping reviews are designed to map the breadth and nature of existing literature rather than evaluate the methodological rigor of included studies. As such, all studies meeting our eligibility criteria were included regardless of quality. Our findings should therefore be interpreted as a comprehensive overview of how hardship areas are defined in SSA, rather than a judgment on the validity or robustness of specific definitions.

## Results

### Included studies

**Fig. 1:**
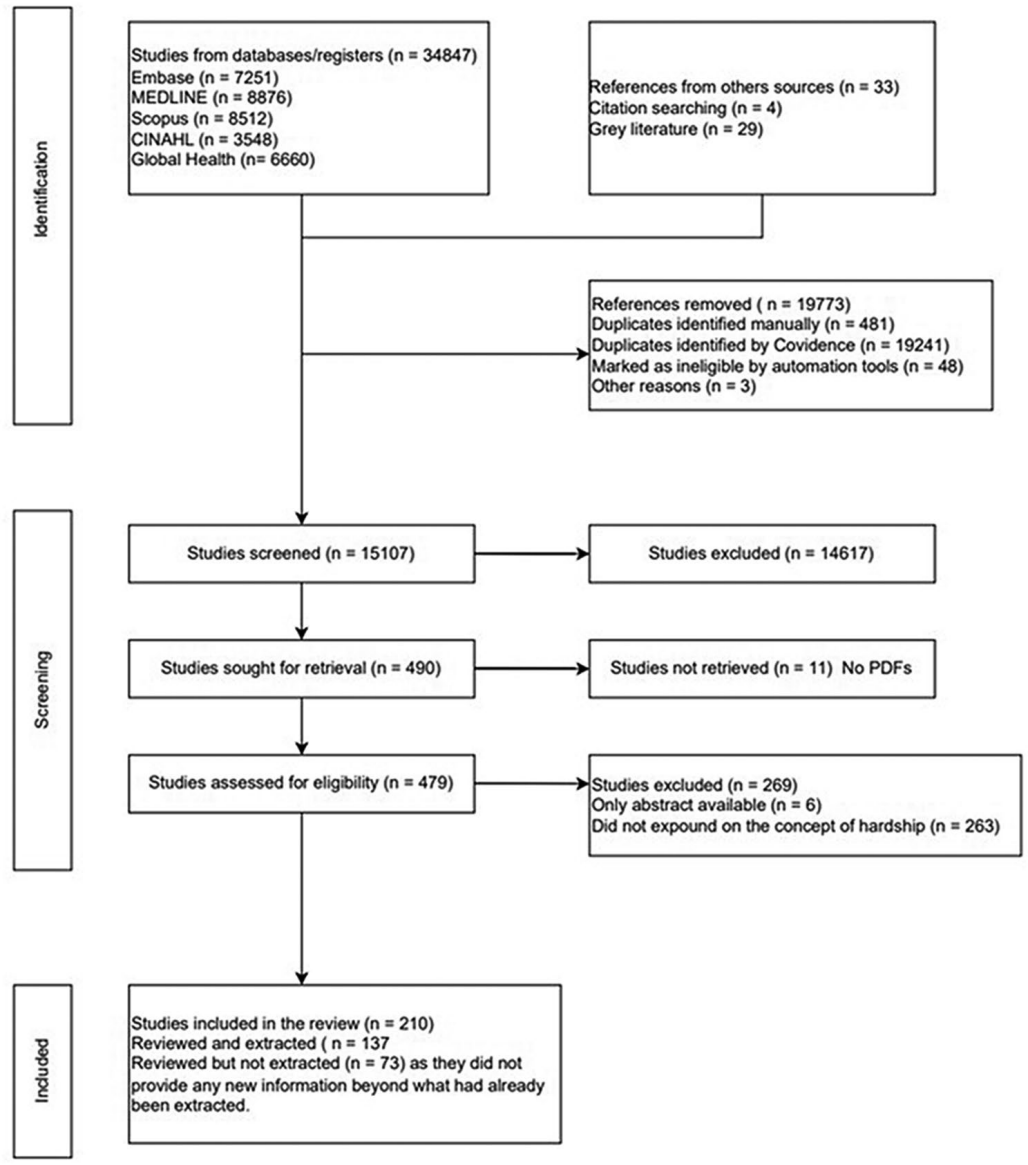
Prisma flow chart showing the study selection process

Of the 15,107 citations screened, 14,617 did not meet the inclusion criteria. An additional 280 citations were excluded at full-text review because 11 were not retrieved, 6 had no full text, and 263 did not define the various synonyms of hardship. Data were extracted from 137 of the remaining 210 studies “see Supplementary Table 3, Additional File 5”. The remaining 73 studies were reviewed but did not yield additional information on features beyond those already extracted. Figure 1 shows the PRISMA flow chart of the review process.

### General characteristics of included studies

**Fig. 2:**
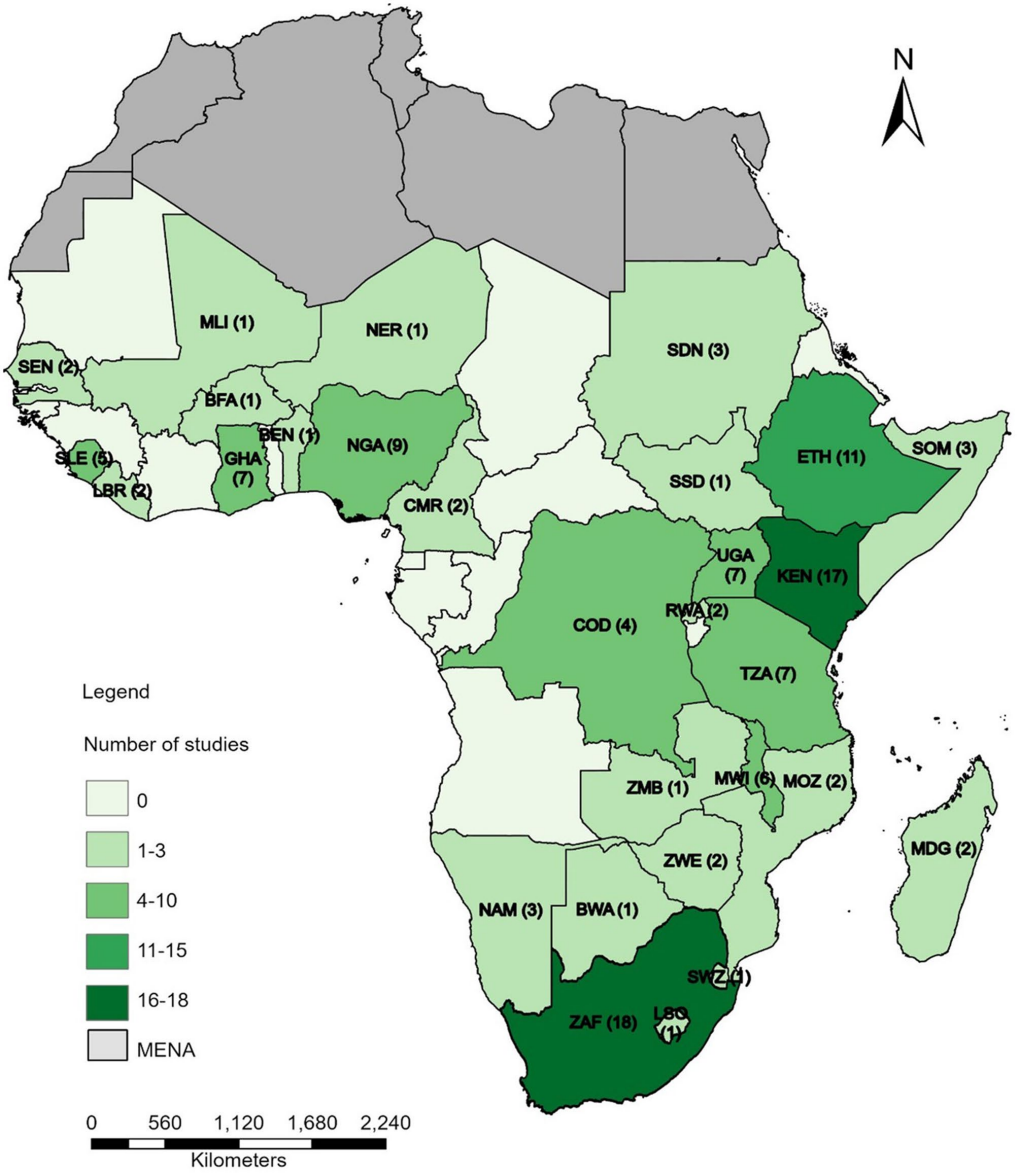
Map showing the distribution of studies in the review across different countries in SSA. The review excluded countries from the Middle East and North Africa region

Publication dates ranged from 2010 to 2024, with most studies published in 2021 (*n* = 20). Most studies were conducted in South Africa, Kenya Ethiopia, and Nigeria, while Uganda, Tanzania, and Ghana were also relatively well represented (Fig. 2). Conversely, a handful of countries, including South Sudan, Niger, and Benin were covered by a single study each (See Fig. 2).

Additionally, some studies focused on broader regions including the Sahel, the Horn of Africa, East Africa, and West Africa. Two studies focused on SSA, while one examined low and middle-income countries (LMICs). Additionally, four studies took a global approach, exploring the hardship concept beyond SSA “See Supplementary Table 4, Additional File 6”.

There were different governmental terms for hardship areas; for instance, Kenya used the term “hardship areas” [20], whereas Uganda referred to them as “hard-to-stay-and-reach areas” [18]. South Africa categorized these areas as rural and underserved [21–23], while Ghana described them as deprived areas [24]. Malawi and Sierra Leone defined them as hard-to-reach areas [25–27], while Senegal identified them as difficult regions [28]. While the terminologies differed, the underlying concept remained consistent, typically denoting areas characterised by difficult living conditions.

### Features of hardship areas in Sub-Saharan Africa

First, we present the features of hardship areas identified in the reviewed literature, which represent the most granular level of synthesis. Secondly, we demonstrate how we grouped these individual features into subthemes and subsequently into broader contextual themes, resulting in an overarching typology.

We identified 79 features (Table 1) associated with hardship areas, drawn from varying definitions, descriptions, and criteria across the 137 included studies. Frequently cited features included limited healthcare access, remoteness, poverty (generally referring to widespread economic deprivation among the resident population), inadequate access to safe water, limited access to electricity, and poor road infrastructure. Although the definitions of remoteness varied, it was commonly described as isolation from urban centres, poor transport networks, or physical distance or travel time from health facilities. Less frequently cited included extreme weather events such as cyclones, wildfires, and mudslides, among others. A word cloud summarizing these features is provided in Supplementary Fig. 2, Additional file 7.

**Table 1:** Features of hardship areas ranked by number of times they appeared in reviewed literature

Features of hardship areas	Frequency (%), N = 137
Limited healthcare access	50 (36.5%)
Remote	38 (27.7%)
Inadequate safe water	32 (23.4%)
Poverty	32 (23.3%)
Limited electricity access	26 (18.9%)
Inadequate sanitation facilities	24 (17.5%)
Poor road infrastructure	23 (16.8%)
Limited education access	19 (13.9%)
Reliance on agriculture	18 (13.1%)
Rural	18 (13.1%)
Poor communication services	17 (12.4%)
Arid and semi-arid	17 (12.4%)
Drought	17 (12.4%)
Low population density	17 (12.4%)
Conflict	16 (11.7%)
Poor housing	16 (11.7%)
Topographical barriers	15 (10.9%)
Unemployment	15 (10.9%)
Floods	14 (10.2%)
Physical barriers	13 (9.5%)
Insecurity	12 (8.8%)
Food insecurity	11 (8.0%)
Weak/unstable institutions	8 (5.8%)
Disease outbreaks, limited recreational facilities, reliance on natural resources	7 (5.1%) each
Low literacy levels	6 (4.4%)
Contentious land tenure, extreme temperatures, high population density, limited economic opportunities, malnutrition, overcrowding, water scarcity	5 (3.6%) each
Hard-to-reach, high disease incidence, low agricultural yields, pollution, unaffordable transport, unsustainable environments	4 (2.9%) each
Landslide, limited banking services, low-income, predominately rural (90% of the population live in rural), small-scale business	3 (2.2%) each
Aging population, dry spells, economic crisis, famine, hunger, limited market access, limited vegetation, outward migration, poor quality land, strong winds, very young population, Sahel climate	2 (1.5%) each
Cyclone, deforestation, dependency on humanitarian aid, ecological degradation, food shortage, high dependency rate, high ecological sensitivity, hostility of locals, Intermittent and costly food supplies, lack of political participation and accountability, land inaccessibility for food production, limited professional personal and family growth, low living standards, no drainage system, no streetlights, non-serviced built environment, poorly planned area, unsustainable irrigation, volcanic eruptions, wildfire, mudslide	1 (0.7%) each

Several studies did not refer to a single feature in isolation but rather described hardship areas using multiple, co-occurring features. For instance, in Nigeria, hard-to-reach areas were defined in one report as having geographically challenging terrain with any of the following criteria: having inter-ward, inter-Local Government Area (LGA), or interstate borders; scattered households; a nomadic population; or a waterlogged or riverine area without easy access to healthcare facilities, and insecurity [29]. 

The reviewed literature also showed that some countries applied specific definitions to these features; for example, in South Africa a low level of income in rural nodes was defined as per capita income of R114.10 per month [21]. Kenya and Uganda applied a feature-weighted scoring system; Kenya assigned a total weight of 100 and a cut-off score of 55, as outlined by the Directorate of Personnel Management in 2008 and cited by the Parliament of the Republic of Kenya [20], while Uganda assigned a total weight of 10 [30]. This diversity in how features appear or are interpreted in various contexts was further illustrated in country-specific terminology and classifications.

### Regional and country variations

Analysis of 137 articles revealed variations in terminologies and specific issues across different countries. Nonetheless, several challenges are shared across SSA, including geographical isolation defined by physical and topographical barriers, inadequate or dilapidated transport infrastructure, and remoteness. These areas also struggle with limited social services including inadequacies in access to electricity, safe water, healthcare, and communication services. Additionally, they face economic limitations like high unemployment and poverty. Natural and man-made disasters, including floods, droughts, arid and semi-arid conditions, and food insecurity occurred frequently, just like insecurity stemming from instability, resource-based tension, and local conflicts.

Some minor feature-related variations were country-specific. For instance, cyclones were frequently reported in Mozambique due to its coastal geography [31]. Landslides were reported in parts of Uganda, Rwanda and Ethiopia, driven by population pressure on limited but fertile soils, as well as topographical, geological, and climate factors [32–34]. In relation to food security challenges, Kenya experience periods of complete food shortage reflecting both availability and accessibility challenges [20]. In contrast, Uganda’s food insecurity is characterized more by intermittent and costly supplies, indicating that food is available nationally but not equitably accessible due to the disruption in supply chain [30]. Rural areas in SSA are characterized by sparse population [35], as evidenced by examples from Tanzania [36], Ghana [37], Nigeria [38], and Malawi [39]. However, South Africa had a mix of both sparsely and densely populated rural areas as features of underserved regions [21, 40].

### Contextual themes

Beyond the granular insights, a more structured and interpretable classification of hardship areas emerges by categorizing these features into 16 subthemes and then into six broader contextual themes, illustrating their multidimensionality.

Six themes were identified to characterise hardship areas (see Table 2): physical/geographical location and accessibility, social services, economic and livelihood challenges, demographic characteristics, natural and man-made disasters, and security and governance. These themes encompass key aspects. They include difficulties in physical access, availability and quality of essential services, economic opportunities and livelihoods, population structure and dynamics, vulnerability to environmental and climate-related shocks, and safety, stability, and governance.

Among the six contextual themes, the most cited were social services 85/137 (62%) followed by physical location/geographical barriers 71/137 (51.8%) while the demographic characteristics theme was the least referenced by 30/137 (21.9%). The major subthemes cited across the 137 articles included human development/quality of life 59/137 (43.1%), agriculture and rural economy 51/137 (37.2%), and basic living conditions 49/137 (35.7%), “See Table 2”.

**Table 2:** Features of hardship areas in Sub-Saharan Africa classified under six themes and 12 sub-themes

Contextual themes	Sub-themes	Meaning of sub-themes	Features of hardship areas	Number (%) of publications (n = 137)
Physical/geographical location and accessibility	Physical location/Geographical barriers	This is in reference to where an area is located and natural barriers that make access difficult	Physical barriers Topographical barriers Rural Hard to reach^1^	43 (31.4%)
	Distance/Time	This is in reference to how long it takes to reach essential services	Remote^2^	36 (26.3%)
	Transport infrastructure	This is in reference to the availability of public transport and condition of roads and bridges	Poor road infrastructure No motorized transport Unaffordable transportation Unreliable transport	35 (25.5%)
Social service	Human development and quality of life	This is reference to social services that support education, healthcare, and recreational opportunities	Limited healthcare access Limited education access Limited recreational facilities High disease incidences	59 (43.1%)
	Basic living conditions	This is in reference to the availability of essential services such as housing, electricity, running water, and sanitation	Limited electricity access Inadequate safe water Poor housing Inadequate sanitation facilities Poorly serviced built environment No drainage system^3^ No streetlights	49 (35.7%)
	Communication/Technology	This is in reference to access to internet, mobile networks, and other forms of communication	Limited internet access Poor communication services Lack of infrastructure (computer)	17 (12.4%)
Economic and livelihood Challenges	Agriculture and rural economy	This is in reference to challenges in agricultural livelihoods and production	Reliance on agriculture Reliance on natural resources Low agricultural yields Limited access to markets Operation of small-scale businesses Land inaccessibility for food production	51 (37.2%)
	Economic and financial constraints	This is in reference to barriers to financial stability such as employment opportunities, income levels, and poverty	Poverty Low income Unemployment Low literacy levels Economic crisis Dependency on humanitarian aid Limited economic opportunities Limited/lack of banking services Low living standards (general economic deprivation) Limited professional, personal, and family growth	24 (17.5%)
Demographic characteristics	Population density and distribution	This is in reference to how people are spread out within an area.	Low population density High population density Overcrowding Predominately rural (90% of the population live in rural)	27 (19.7%)
	Age dynamics	This is in reference to age structure and population composition	High dependency rate Very young population Aging population	4 (2.9%)
	Mobility and migration	This is in reference to the movement of people from one area to another	Outward migration (Rural-urban migration and population displacement)	3 (2.2%)
Natural and Man-made disasters	Climate factors	This is in reference to the extreme weather conditions such as droughts	Arid and semi-arid climate Sahel climate Dry spells Extreme temperatures Drought Floods Cyclones Wildfires Strong winds Disease outbreaks (cholera, seasonal malaria)	39 (28.5%)
	Food & water scarcity	This is in reference to availability, accessibility, and affordability of food and water	Food insecurity/Famine Food shortage / Intermittent and costly food supplies Malnutrition Hunger Water scarcity	20 (14.6%)
	Environmental challenges	This is in reference to factors that affect living conditions and sustainability such as pollution, landslides/mudslides	Landslides Mudslide Poor quality land Unsustainable environment^4^ Pollution Limited vegetation Volcanic eruptions- (exposure to Sulphur dioxide) Deforestation Ecological degradation Unsustainable irrigation Poorly planned area High ecological sensitivity	17 (12.4%)
Security and governance	Security	This is in reference to threats to safety and stability such as the presence of crime	Insecurity^5^ Conflict (armed or violent conflict) Hostility of locals	30 (21.9%)
	Governance	This is in reference to political structures, policy failures, and governance challenges	Lack of political participation and accountability Weak/unstable institutions^6^ Contentious land tenure	14 (10.2%)

^1^ Hard to reach-this is in reference to physical barriers and dilapidated transportation that make access difficult.

^2^ Remote- this is in reference to geographical barriers that delay access/ due to distances from capital cities, nearest towns, hospitals, drug shops/due to travel time

^3^ No drainage systems-this is in reference to water flow and management-rainwater, wastewater)

^4^ Unsustainable environment-this is in reference to hazardous locations such as located along floodplain, seashore, river basin, areas prone to underground fires)

^5^ Insecurity (insurgency, high crime rate, human-wildlife conflict). Conflict (related to wars)

^6^ Weak/unstable institutions (corruption, political instability, political arbitrariness, poor law enforcement)

**Fig. 3:**
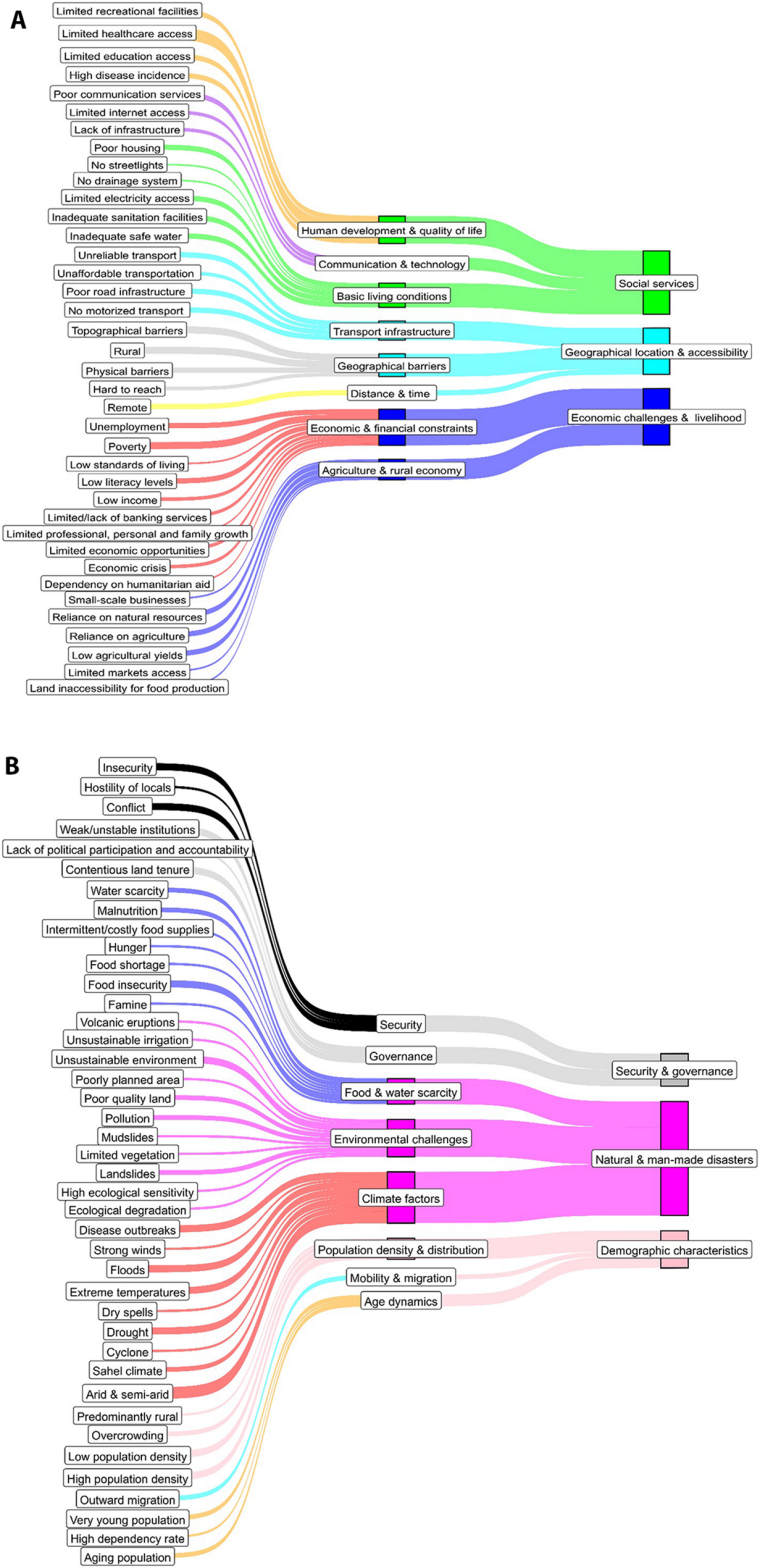
**a** Sankey diagram linking hardship area features, eight subthemes, and contextual themes on social services, geography, and economy. ** b** Sankey diagram linking hardship area features, eight subthemes, and contextual themes on security, and disasters, and demographics

The Sankey diagrams in Fig. 3a and b depict the relationship between features, subthemes, and contextual themes reported in Table 2.

## Discussion

In our review, we synthesized 137 articles published between 2010 and 2024 to explore how hardship is defined and characterised across SSA. We identified 79 relatively distinct features, with limited healthcare access, remoteness, and poverty being the most cited. We grouped the features into six contextual themes (see Fig. 3a and b). This thematic grouping reflects the multifaceted nature of hardship.

Features like remoteness and poverty frequently appear across definitions, yet they are often insufficiently unpacked, raising concerns about their conceptualisation and policy application. Remoteness is commonly equated to distance or travel time, which may misclassify regions where the primary causes of hardship are beyond geographic location. Similarly, poverty can be determined by asset ownership, income, or access to opportunities or services. Therefore, using it as a blanket descriptor obscures distinction between social exclusion, institutional neglect, and economic deprivation, hindering the design of tailored interventions. Additionally, blanket descriptors oversimplify hardship framing it as logistical issue rather than a structural problem. This calls for precise and contextual framing to inform effective interventions.

At its core, this review demonstrates that hardship is a widely recognized concept, yet its features are context-dependent, inconsistently defined and applied across settings. Hardship is framed using different terms such as “hard-to-reach,” “deprived,” or “hard-to-reach-and-stay,” which are shaped by political and local administrative contexts. The objective indicators like distance dominate official definitions; they are easy to quantify and integrate in planning [41] but do not capture the lived experiences of hardship. Subjective indicators like hostility from local people and perceived isolation [31], are frequently overlooked despite their influence on both service delivery and workforce retention. These definitional discrepancies, coupled with neglect of subjective experiences, limit the usefulness of evaluations of strategies that aim to address hardship, improve service delivery, and workforce retention as well as equitable development.

The synthesis of 137 articles reveals that the designation of hardship areas is a political process, shaped by bargaining, historical oversight, and administrative expediency rather than impartial evaluation of need. For instance, in Kenya, when the Teachers Service Remuneration Committee (TSRC) first proposed a list of hardship areas and hardship allowance rates, the then Minister for Education demanded that his constituency, a wealthy area, be included in the list of hardship areas before he would ratify the document. The TSRC members subsequently included their home regions on the list too [42]. Such practices illustrate how decisions regarding staff deployment and resources allocation may be driven by political interests rather than rather than severity of hardship. This undermines fairness, transparency and long-term accountability in efforts to promote equitable development.

To address this definitional gap, we developed a typology of the features grouped into 16 subthemes nested within six themes “See Table 2”. This typology provides a structured framework for understanding and assessing the features that characterise hardship areas. It enables linkage to geospatial data and indicators to map areas of hardship. The typology provides a useful starting point for governments seeking to formalise or revise the designation of hardship areas. This typology should be seen as a flexible tool rather than a fixed blueprint. Policymakers can use it to tailor workforce retention incentives and guide equitable allocation of resources by accounting for diverse contextual challenges such as limited social services, poor infrastructure, or insecurity.

The findings show that physical and geographical location significantly contribute to the hardship status of localities in SSA. Geographically, hardship areas are often viewed as predominantly rural; however, our findings challenge this rural-centric perspective. This is because informal urban settlements in SSA also experience poor road networks hindering access in cases of emergencies [43]. Additionally, many are located in hazardous zones such as along riverbanks, in floodplains, and on sewer lines [44]. Framing hardship solely as a rural issue risks excluding vulnerable urban populations and undermining inclusive policy design. Consequently, the delineation of hardship areas should account for the rural-urban spectrum.

Limited access to social services is a significant contributor to hardship in SSA. The findings emphasise that hardship is not just a service issue but includes political and historical aspects [42]. In some areas, marginalisation stems from colonial policies and practices coupled with insufficient post-colonial corrective redistributive measures [45]. The administrative and investment frameworks of these policies often favoured economically significant regions while systematically disregarding others. Consequently, some regions continue to suffer generations of underdeveloped infrastructure with minimal state presence, which reinforces cycles of marginalisation. Therefore, addressing service issues in hardship areas requires a deeper understanding of political economy and how power and resources are shared.

The review further emphasises that economic and livelihood challenges mirror hardship areas. This phenomenon reflects structural inequities that are more often part of national development pathways. The persistent rural-urban divide, characterized by infrastructural underdevelopment in rural areas, is an example [46]. This urban-centric investment contributes to a declining rural connectivity. Mitigating excessive urban bias in public investment can lead to balanced growth and reduce poverty in rural areas [47]. This is because efficient transport systems generate economic opportunities and multiplier effects like improved market accessibility and employment [48]. To support rural development and alleviate poverty calls for sharper policy focus with more balanced investment.

Climate-related factors, though less frequently referenced, play a significant and increasing role in exacerbating hardship. This is because climate change has altered disease patterns and disrupted agricultural livelihoods. Moreso, it has led to displacement and resource-based conflicts as residents scramble for the few existing resources. As the climate changes, so will the current context in hardship areas. Therefore, in subsequent work we intend to explore the shift in hardship with climate change.

Although a widely acknowledged concept, at the policy level there is limited conceptualisation and comprehension of hardship. Many definitions are confined to infrastructural deficiencies failing to encompass the lived experiences of grassroot stakeholders. The gap between official and grassroot perceptions reflects a top-down framing, shaped more by urban viewpoint of rural life than by lived realities [42]. Moving forward, we propose participatory validation of the typology to integrate the viewpoints of grassroots stakeholders. This will ensure that the typology integrates technical and structural features, while also reflecting the lived experiences of individuals in hardship areas. In doing so, the typology becomes legitimate, actionable, and sustainable. In addition, we will evaluate the effectiveness of the typology through geospatial mapping to assess whether spatial visualisation identifies hardship zones more accurately than current method. Such improvements could strengthen targeting and promote equity in resource distribution.

We recommend that governments and regional bodies move from evidence to action. This necessitates the prioritisation of developing standardised but flexible frameworks for the designation of hardship areas. These frameworks should be grounded in multidimensional criteria, while remaining adaptable to local contexts and applicable for planning, budgeting, and accountability. Cross-sectoral collaboration is also necessary because hardship is not just a health issue; it signifies systemic failure across various sectors. Therefore, incentive packages should be integrated within comprehensive investments in infrastructure, education, and governance reform.

### Strengths and limitations

The strength of this review lies in the application of the Arksey and O’Malley framework for scoping reviews which enables collaborative engagement in the review process [13]. This entailed formulating a research topic and developing key terms to identify pertinent studies. There are several limitations to considered in this review. First, many government documents containing the criteria for defining hardship areas were not available online. Second, very few articles originated from primary research involving interviews with individuals living in hardship areas, which limited synthesis of lived experiences [5, 49]. Third, our focus on three countries under the AFRHiCARE partnership limited opportunities for wider policy comparison. Additionally, grey literature from other SSA countries was not extensively captured, which may have constrained the breadth of contextual insights. Finally, only English-language publications were included, which may have excluded relevant evidence published in other languages.

## Conclusion

This review illustrates that hardship in SSA is a context-driven, historically rooted, and multidimensional notion. Therefore, there is need to move beyond the narrow definitions and embrace inclusive frameworks that capture objective indicators and subjective realities. The typology developed in this review lays the groundwork for geospatial characterisation of hardship, bridging definitional gaps and informing transparent, effective, and equitable policy responses. However, the challenge is not solely technical; progress in addressing issues in hardship areas also depends on cross-sectoral collaboration, structural reforms, and commitment to amplifying the voices of those most affected.

## Supplementary Information

Below is the link to the electronic supplementary material.

Supplementary Material 1: File name: Additional file 1. File format: Doc (Microsoft word). Title of data: PRISMA-ScR checklist.. Description: Completed PRISMA Extension for Scoping Reviews (PRISMA-ScR) checklist used to ensure transparent reporting of the review process

Supplementary Material 2: File name: Additional file 2. File format: Doc (Microsoft word). Title of data: Supplementary Table 2: Deviations from the protocol.. Description: Summary of any deviations from the register scoping review protocol, including justification

Supplementary Material 3: File name: Additional file 3. File format: Doc (Microsoft word). Title of data: Search strings. Description: Complete search strategies used across the five databases (Medline, Scopus, CINAHL, Embase, and Global Health)

Supplementary Material 4: File name: Additional file 4. File format: Doc (Microsoft word). Title of data: Supplementary figure 1: Development of thematic categories for defining hardship areas. Description: Diagram illustrating the process used to develop thematic categories for defining hardship areas

Supplementary Material 5: File name: Additional file 5. File format: Doc (Microsoft word). Title of data: Supplementary Table 3: Overview of Key Characteristics of included studies. Description: Summary table presenting key characteristics of the studies included in the scoping review, including author, year, country, study design, data sources, and definitions or indicators of hardship areas

Supplementary Material 6: File name: Additional file 6. File format: Doc (Microsoft word). Title of data: Supplementary Table 4: Number of studies conducted in specific countries and regions. Description: Table summarizing the distribution of included studies by country and region within SSA, highlighting areas with high or low representation

Supplementary Material 7: File name: Additional file 7. File format: Doc (Microsoft word). Title of data: Supplementary Figure 2: Word cloud showing the features of hardship areas in Sub-Saharan Africa. Description: Visual representation of the frequently mentioned features of hardship areas in SSA, generated from the extracted data to highlight commonly occurring concepts

## Data Availability

The supplementary files, in-text table, and figures provided contain all the data.

## Abbreviations

SDGs: Sustainable Development Goals
UHC: Universal Health Coverage
SSA: Sub Saharan Africa
WHO: World Health Organization
OSF: Open Science Framework
