# Supplementary material for: Conceptualising hardship areas in Sub-Saharan Africa: a scoping review
Source: Int J Equity Health. 2025 Nov 21;24:326. doi: 10.1186/s12939-025-02694-x (PMC12639685; doi:10.1186/s12939-025-02694-x)
Supplement: Supplementary file 2 — Supplementary Material 2: File name: Additional file 2. File format: Doc (Microsoft word). Title of data: Supplementary Table 2: Deviations from the protocol.. Description: Summary of any deviations from the register scoping review protocol, including justification [file 12939_2025_2694_MOESM2_ESM.docx]

| **Section** | **Original Protocol** | **Deviation from protocol** | **Rationale** |
| --- | --- | --- | --- |
| Study selection | Full text review will be conducted by first and second authors. | Full text review was conducted by one reviewer, while the second reviewed only 20% | The increased workload and more engaging tasks prevented both authors from reviewing every article. To address this, we adhered to the guidelines provided by Mak & Thomas (2022), which recommend that if it is not possible for each article to be reviewed by two reviewers, one reviewer can lead the review while the second reviewer assesses a subset of the studies. This approach ensures that at least 20% of the articles are reviewed by both reviewers, with a minimum agreement rate of 90%. |
| Data extraction/ Charting of the data | The first author will extract and chart data, with the second and third authors checking a 10% sample of the data extraction, to look for systematic errors. | CM led the data extraction process, with PK extracting 20% of the data. Senior reviewers EAO, JO, and ME validated the charted data. | We adjusted the initial workload due to workload constraints. The situation necessitated a redistribution of tasks to ensure timely completion and maintain the quality of the review process. |
| Search strategy | The original approach was to have one concept, which is different terms to mean hardship areas | We added a third concept “Health” in Scopus and Ovid Embase | The reason behind this was to refine the search results and make them more manageable. |

**Supplementary Table 2: Deviations from the protocol**
