# Supplementary material for: Conceptualising hardship areas in Sub-Saharan Africa: a scoping review
Source: Int J Equity Health. 2025 Nov 21;24:326. doi: 10.1186/s12939-025-02694-x (PMC12639685; doi:10.1186/s12939-025-02694-x)
Supplement: Supplementary file 3 — Supplementary Material 3: File name: Additional file 3. File format: Doc (Microsoft word). Title of data: Search strings. Description: Complete search strategies used across the five databases (Medline, Scopus, CINAHL, Embase, and Global Health) [file 12939_2025_2694_MOESM3_ESM.docx]

**Additional file 3: Search strings**

## **Ovid Medline**

1 (rural and (Incentive* or reward or Allowance or retention or recruitment or motivation or underserved or marginalized))

2 ("resource-limited setting*" and (underserved or marginalized*))

3 ((poverty or impoverished) and (area* or setting* or location* or region or underserved or marginalized))

4 ("low income setting*" and (underserved or marginalized* or marginalised*))

5 (remote and (Incentive* or underserved or marginalized or reward or Allowance or retention or recruitment or motivation))

6 (refugee* or migrant* or "infectious disease outbreak*" or "armed conflict*" or "exposure to conflict" or warfare or "urban slum*" or "informal settlement*" or "underserved area*" or famine or "fragile state*" or "small island*" or landslide* or "conflict zone*" or "hard-to-reach area*" or "disadvantaged area*" or earthquake* or "deprived area*" or "resource constrained area*" or "access-to-social service*" or "geographical isolation" or "extreme heat" or "remote island*" or "poor resource setting*" or "arid area*" or "war torn" or terrorism or "marginalized area*" or "war exposure" or "isolated setting*" or "difficult-to-access area*" or "violent crime*" or "under-served area*" or "low-access area*" or "rural remote area*" or "under serviced area*" or "under-developed area*" or "flood-prone area*" or hurricane* or "access-to- social amenities" or "out-of-reach area*" or non-metropolitan or "less* developed area*" or "under developed area*" or "drought prone area*" or "unreliable rainfall" or "remote underserviced area*" or "geographic* isolated location*" or "hard-to-staff" or "secluded area*" or "hard-to-access area*" or "geographically isolated area*" or nonmetropolitan or non-metropolitan or bushfire* or "semi-arid area*" or "Inhospitable area*" or "post conflict zone*" or Tsunami* or Tornado* or typhoon* or "marine disaster*" or insecurit*)

7 "food insecurity"

8 6 not 7

9 1 or 2 or 3 or 4 or 5 or 8

10 ("Africa South of Sahara" or "Sub-Saharan Africa" or "Subsaharan Africa" or Angola or Benin or Botswana or "Burkina Faso" or Burundi or "Cabo Verde" or Cameroon or "Central African Republic" or Chad or Comoros or "Republic of Congo" or "Democratic Republic of Congo" or Djibouti or "Cote d$Ivoire" or "Equatorial Guinea" or Eritrea or Eswatini or Ethiopia or Gabon or "The Gambia" or Ghana or "Guinea-Bissau" or Kenya or Lesotho or Liberia or Madagascar or Malawi or Mali or Mauritania or Mauritius or Mozambique or Namibia or Niger or Nigeria or Rwanda or "Sao Tome and Principe" or Senegal or Seychelles or "Sierra Leone" or Somalia or "South Africa" or "South Sudan" or Sudan or Tanzania or Togo or Uganda or Zambia or Zimbabwe or Guinea)

11 ("papua new guinea" or "guinea pig*")

12 10 not 11

13 exp "Africa south of the Sahara"/

14 12 or 13

15 9 and 14

16 limit 15 to (english language and yr="2010 -Current")

**Ovid Global Health**

1 (rural and (Incentive* or reward or Allowance or retention or recruitment or motivation or underserved or marginalized)).ti,ab. 4997

2 ("resource-limited setting*" and (underserved or marginalized*)).ti,ab. 31

3 ((poverty or impoverished) and (area* or setting* or location* or region or underserved or marginalized)).ti,ab. 8435

4 ("low income setting*" and (underserved or marginalized* or marginalised*)).ti,ab. 10

5 (remote and (Incentive* or underserved or marginalized or reward or Allowance or retention or recruitment or motivation)).ti,ab. 1008

6 (refugee* or migrant* or "infectious disease outbreak*" or "armed conflict*" or "exposure to conflict" or warfare or "urban slum*" or "informal settlement*" or "underserved area*" or famine or "fragile state*" or "small island*" or landslide* or "conflict zone*" or "hard-to-reach area*" or "disadvantaged area*" or earthquake* or "deprived area*" or "resource constrained area*" or "access-to-social service*" or "geographical isolation" or "extreme heat" or "remote island*" or "poor resource setting*" or "arid area*" or "war torn" or terrorism or "marginalized area*" or "war exposure" or "isolated setting*" or "difficult-to-access area*" or "violent crime*" or "under-served area*" or "low-access area*" or "rural remote area*" or "under serviced area*" or "under?developed area*" or "flood-prone area*" or hurricane* or "access-to- social amenities" or "out-of-reach area*" or non-metropolitan or "less* developed area*" or "under developed area*" or "drought prone area*" or "unreliable rainfall" or "remote underserviced area*" or "geographic* isolated location*" or "hard-to-staff" or "secluded area*" or "hard-to-access area*" or "geographically isolated area*" or nonmetropolitan or non-metropolitan or bushfire* or "semi-arid area*" or "Inhospitable area*" or "post conflict zone*" or Tsunami* or Tornado* or typhoon* or "marine disaster*" or insecurit*).ti,ab. 47115

7 "food insecurity".ti,ab. 8459

8 6 not 7 38656

9 1 or 2 or 3 or 4 or 5 or 8 51246

10 ("Africa South of Sahara" or "Sub-Saharan Africa" or "Subsaharan Africa" or Angola or Benin or Botswana or "Burkina Faso" or Burundi or "Cabo Verde" or Cameroon or "Central African Republic" or Chad or Comoros or "Republic of Congo" or "Democratic Republic of Congo" or Djibouti or "Cote d$Ivoire" or "Equatorial Guinea" or Eritrea or Eswatini or Ethiopia or Gabon or "The Gambia" or Ghana or "Guinea-Bissau" or Kenya or Lesotho or Liberia or Madagascar or Malawi or Mali or Mauritania or Mauritius or Mozambique or Namibia or Niger or Nigeria or Rwanda or "Sao Tome and Principe" or Senegal or Seychelles or "Sierra Leone" or Somalia or "South Africa" or "South Sudan" or Sudan or Tanzania or Togo or Uganda or Zambia or Zimbabwe or Guinea).ti,ab. 233179

11 ("papua new guinea" or "guinea pig*").ti,ab. 10501

12 10 not 11 222678

13 exp "africa south of sahara"/ 237783

14 12 or 13 261111

15 9 and 14 9068

16 15 9068

17 limit 16 to (english language and yr="2010 -Current") 6660

**Ovid Embase**

1 (rural and (Incentive* or reward or Allowance or retention or recruitment or motivation or underserved or marginalized)).ti,ab,kf. 12344

2 ("resource-limited setting*" and (underserved or marginalized*)).ti,ab,kf. 112

3 ((poverty or impoverished) and (area* or setting* or location* or region or underserved or marginalized)).ti,ab,kf. 17275

4 ("low income setting*" and (underserved or marginalized* or marginalised*)).ti,ab,kf. 27

5 (remote and (Incentive* or underserved or marginalized or reward or Allowance or retention or recruitment or motivation)).ti,ab,kf. 6004

6 (refugee* or migrant* or "infectious disease outbreak*" or "armed conflict*" or "exposure to conflict" or warfare or "urban slum*" or "informal settlement*" or "underserved area*" or famine or "fragile state*" or "small island*" or landslide* or "conflict zone*" or "hard-to-reach area*" or "disadvantaged area*" or earthquake* or "deprived area*" or "resource constrained area*" or "access-to-social service*" or "geographical isolation" or "extreme heat" or "remote island*" or "poor resource setting*" or "arid area*" or "war torn" or terrorism or "marginalized area*" or "war exposure" or "isolated setting*" or "difficult-to-access area*" or "violent crime*" or "under-served area*" or "low-access area*" or "rural remote area*" or "under serviced area*" or "under?developed area*" or "flood-prone area*" or hurricane* or "access-to- social amenities" or "out-of-reach area*" or non-metropolitan or "less* developed area*" or "under developed area*" or "drought prone area*" or "unreliable rainfall" or "remote underserviced area*" or "geographic* isolated location*" or "hard-to-staff" or "secluded area*" or "hard-to-access area*" or "geographically isolated area*" or nonmetropolitan or non-metropolitan or bushfire* or "semi-arid area*" or "Inhospitable area*" or "post conflict zone*" or Tsunami* or Tornado* or typhoon* or "marine disaster*" or insecurit*).ti,ab,kf. 129508

7 "food insecurity".ti,ab,kf. 11854

8 6 not 7 117654

9 1 or 2 or 3 or 4 or 5 or 8 148570

10 ("Africa South of Sahara" or "Sub-Saharan Africa" or "Subsaharan Africa" or Angola or Benin or Botswana or "Burkina Faso" or Burundi or "Cabo Verde" or Cameroon or "Central African Republic" or Chad or Comoros or "Republic of Congo" or "Democratic Republic of Congo" or Djibouti or "Cote d$Ivoire" or "Equatorial Guinea" or Eritrea or Eswatini or Ethiopia or Gabon or "The Gambia" or Ghana or "Guinea-Bissau" or Kenya or Lesotho or Liberia or Madagascar or Malawi or Mali or Mauritania or Mauritius or Mozambique or Namibia or Niger or Nigeria or Rwanda or "Sao Tome and Principe" or Senegal or Seychelles or "Sierra Leone" or Somalia or "South Africa" or "South Sudan" or Sudan or Tanzania or Togo or Uganda or Zambia or Zimbabwe or Guinea).ti,ab,kf. 460577

11 ("papua new guinea" or "guinea pig*").ti,ab,kf. 100024

12 10 not 11 360553

13 exp "Africa south of the Sahara"/ 331787

14 12 or 13 421899

15 health*.mp. 7109020

16 9 and 14 and 15 8669

17 limit 16 to (english language and yr="2010 -Current") 7251

**SCOPUS**

( ( ( ( TITLE-ABS-KEY ( ( refugee* OR migrant* OR "infectious disease outbreak*" OR "armed conflict*" OR "exposure to conflict" OR warfare OR "urban slum*" OR "informal settlement*" OR "underserved area*" OR famine OR "fragile state*" OR "small island*" OR landslide* OR "conflict zone*" OR "hard-to-reach area*" OR "disadvantaged area*" OR earthquake* OR "deprived area*" OR "resource constrained area*" OR "access-to-social service*" OR "geographical isolation" OR "extreme heat" OR "remote island*" OR "poor resource setting*" OR "arid area*" OR "war torn" OR terrorism OR "marginalized area*" OR "war exposure" OR "isolated setting*" OR "difficult-to-access area*" OR "violent crime*" OR "under-served area*" OR "low-access area*" OR "rural remote area*" OR "under serviced area*" OR "under?developed area*" OR "flood-prone area*" OR hurricane* OR "access-to- social amenities" OR "out-of-reach area*" OR non-metropolitan OR "less* developed area*" OR "under developed area*" OR "drought prone area*" OR "unreliable rainfall" OR "remote underserviced area*" OR "geographic* isolated location*" OR "hard-to-staff" OR "secluded area*" OR "hard-to-access area*" OR "geographically isolated area*" OR nonmetropolitan OR non-metropolitan OR bushfire* OR "semi-arid area*" OR "Inhospitable area*" OR "post conflict zone*" OR tsunami* OR tornado* OR typhoon* OR "marine disaster*" OR insecurit* ) ) ) AND NOT ( TITLE-ABS-KEY ( "food insecurity" ) ) ) OR ( TITLE-ABS-KEY ( ( remote AND ( incentive* OR underserved OR marginalized OR reward OR allowance OR retention OR recruitment OR motivation ) ) ) ) OR ( TITLE-ABS-KEY ( ( "low income setting*" AND ( underserved OR marginalized* OR marginalised* ) ) ) ) OR ( TITLE-ABS-KEY ( ( ( poverty OR impoverished ) AND ( area* OR setting* OR location* OR region OR underserved OR marginalized ) ) ) ) OR ( TITLE-ABS-KEY ( ( "resource-limited setting*" AND ( underserved OR marginalized* ) ) ) ) OR ( TITLE-ABS-KEY ( rural AND ( incentive* OR reward OR allowance OR retention OR recruitment OR motivation OR underserved OR marginalized ) ) ) ) AND ( ( TITLE-ABS-KEY ( "Africa South of Sahara" OR "Sub-Saharan Africa" OR "Subsaharan Africa" OR angola OR benin OR botswana OR "Burkina Faso" OR burundi OR "Cabo Verde" OR cameroon OR "Central African Republic" OR chad OR comoros OR "Republic of Congo" OR "Democratic Republic of Congo" OR djibouti OR "Cote d$Ivoire" OR "Equatorial Guinea" OR eritrea OR eswatini OR ethiopia OR gabon OR "The Gambia" OR ghana OR "Guinea-Bissau" OR kenya OR lesotho OR liberia OR madagascar OR malawi OR mali OR mauritania OR mauritius OR mozambique OR namibia OR niger OR nigeria OR rwanda OR "Sao Tome and Principe" OR senegal OR seychelles OR "Sierra Leone" OR somalia OR "South Africa" OR "South Sudan" OR sudan OR tanzania OR togo OR uganda OR zambia OR zimbabwe OR guinea ) ) AND NOT ( TITLE-ABS-KEY ( "papua new guinea" OR "guinea pig*" ) ) ) ) AND ( TITLE-ABS-KEY ( health* ) ) AND PUBYEAR > 2009 AND PUBYEAR < 2025 AND ( LIMIT-TO ( LANGUAGE , "English" ) ) AND ( LIMIT-TO ( EXACTKEYWORD , "Human" ) OR LIMIT-TO ( EXACTKEYWORD , "Humans" ) )

**CINAHL via EBSCOhost**

| **#** | **Query** | **Results** |
| --- | --- | --- |
| S1 | TI ( rural and (Incentive* or reward or Allowance or retention or recruitment or motivation or underserved or marginalized) ) OR AB ( rural and (Incentive* or reward or Allowance or retention or recruitment or motivation or underserved or marginalized) ) | 4,782 |
| S2 | TI ( ("resource-limited setting*" and (underserved or marginalized*)) ) OR AB ( ("resource-limited setting*" and (underserved or marginalized*)) ) | 21 |
| S3 | TI ( ((poverty or impoverished) and (area* or setting* or location* or region or underserved or marginalized)) ) OR AB ( ((poverty or impoverished) and (area* or setting* or location* or region or underserved or marginalized)) ) | 5,313 |
| S4 | TI ( ("low income setting*" and (underserved or marginalized* or marginalised*)) ) OR AB ( ("low income setting*" and (underserved or marginalized* or marginalised*)) ) | 10 |
| S5 | TI ( (remote and (Incentive* or underserved or marginalized or reward or Allowance or retention or recruitment or motivation)) ) OR AB ( (remote and (Incentive* or underserved or marginalized or reward or Allowance or retention or recruitment or motivation)) ) | 1,346 |
| S6 | TI ( (refugee* or migrant* or "infectious disease outbreak*" or "armed conflict*" or "exposure to conflict" or warfare or "urban slum*" or "informal settlement*" or "underserved area*" or famine or "fragile state*" or "small island*" or landslide* or "conflict zone*" or "hard-to-reach area*" or "disadvantaged area*" or earthquake* or "deprived area*" or "resource constrained area*" or "access-to-social service*" or "geographical isolation" or "extreme heat" or "remote island*" or "poor resource setting*" or "arid area*" or "war torn" or terrorism or "marginalized area*" or "war exposure" or "isolated setting*" or "difficult-to-access area*" or "violent crime*" or "under-served area*" or "low-access area*" or "rural remote area*" or "under serviced area*" or "under?developed area*" or "flood-prone area*" or hurricane* or "access-to- social amenities" or "out-of-reach area*" or non-metropolitan or "less* developed area*" or "under developed area*" or "drought prone area*" or "unreliable rainfall" or "remote underserviced area*" or "geographic* isolated location*" or "hard-to-staff" or "secluded area*" or "hard-to-access area*" or "geographically isolated area*" or nonmetropolitan or non-metropolitan or bushfire* or "semi-arid area*" or "Inhospitable area*" or "post conflict zone*" or Tsunami* or Tornado* or typhoon* or "marine disaster*" or insecurit*) ) OR AB ( (refugee* or migrant* or "infectious disease outbreak*" or "armed conflict*" or "exposure to conflict" or warfare or "urban slum*" or "informal settlement*" or "underserved area*" or famine or "fragile state*" or "small island*" or landslide* or "conflict zone*" or "hard-to-reach area*" or "disadvantaged area*" or earthquake* or "deprived area*" or "resource constrained area*" or "access-to-social service*" or "geographical isolation" or "extreme heat" or "remote island*" or "poor resource setting*" or "arid area*" or "war torn" or terrorism or "marginalized area*" or "war exposure" or "isolated setting*" or "difficult-to-access area*" or "violent crime*" or "under-served area*" or "low-access area*" or "rural remote area*" or "under serviced area*" or "under?developed area*" or "flood-prone area*" or hurricane* or "access-to- social amenities" or "out-of-reach area*" or non-metropolitan or "less* developed area*" or "under developed area*" or "drought prone area*" or "unreliable rainfall" or "remote underserviced area*" or "geographic* isolated location*" or "hard-to-staff" or "secluded area*" or "hard-to-access area*" or "geographically isolated area*" or nonmetropolitan or non-metropolitan or bushfire* or "semi-arid area*" or "Inhospitable area*" or "post conflict zone*" or Tsunami* or Tornado* or typhoon* or "marine disaster*" or insecurit*) ) | 44,626 |
| S7 | S1 OR S2 OR S3 OR S4 OR S5 OR S6 | 54,178 |
| S8 | TI ( "Africa South of Sahara" or "Sub-Saharan Africa" or "Subsaharan Africa" or Angola or Benin or Botswana or "Burkina Faso" or Burundi or "Cabo Verde" or Cameroon or "Central African Republic" or Chad or Comoros or "Republic of Congo" or "Democratic Republic of Congo" or Djibouti or "Cote d$Ivoire" or "Equatorial Guinea" or Eritrea or Eswatini or Ethiopia or Gabon or "The Gambia" or Ghana or "Guinea-Bissau" or Kenya or Lesotho or Liberia or Madagascar or Malawi or Mali or Mauritania or Mauritius or Mozambique or Namibia or Niger or Nigeria or Rwanda or "Sao Tome and Principe" or Senegal or Seychelles or "Sierra Leone" or Somalia or "South Africa" or "South Sudan" or Sudan or Tanzania or Togo or Uganda or Zambia or Zimbabwe or Guinea ) OR AB ( "Africa South of Sahara" or "Sub-Saharan Africa" or "Subsaharan Africa" or Angola or Benin or Botswana or "Burkina Faso" or Burundi or "Cabo Verde" or Cameroon or "Central African Republic" or Chad or Comoros or "Republic of Congo" or "Democratic Republic of Congo" or Djibouti or "Cote d$Ivoire" or "Equatorial Guinea" or Eritrea or Eswatini or Ethiopia or Gabon or "The Gambia" or Ghana or "Guinea-Bissau" or Kenya or Lesotho or Liberia or Madagascar or Malawi or Mali or Mauritania or Mauritius or Mozambique or Namibia or Niger or Nigeria or Rwanda or "Sao Tome and Principe" or Senegal or Seychelles or "Sierra Leone" or Somalia or "South Africa" or "South Sudan" or Sudan or Tanzania or Togo or Uganda or Zambia or Zimbabwe or Guinea ) | 83,953 |
| S9 | S7 AND S8 | 4,035 |
| S10 | S7 AND S8 | 3,550 |
| S11 | S7 AND S8 | 3,548 |
