# Supplementary figures and images for: Conceptualising hardship areas in Sub-Saharan Africa: a scoping review
Source: Int J Equity Health. 2025 Nov 21;24:326. doi: 10.1186/s12939-025-02694-x (PMC12639685; doi:10.1186/s12939-025-02694-x)

**Supplementary figure 1: Development of thematic categories for defining hardship areas**

**
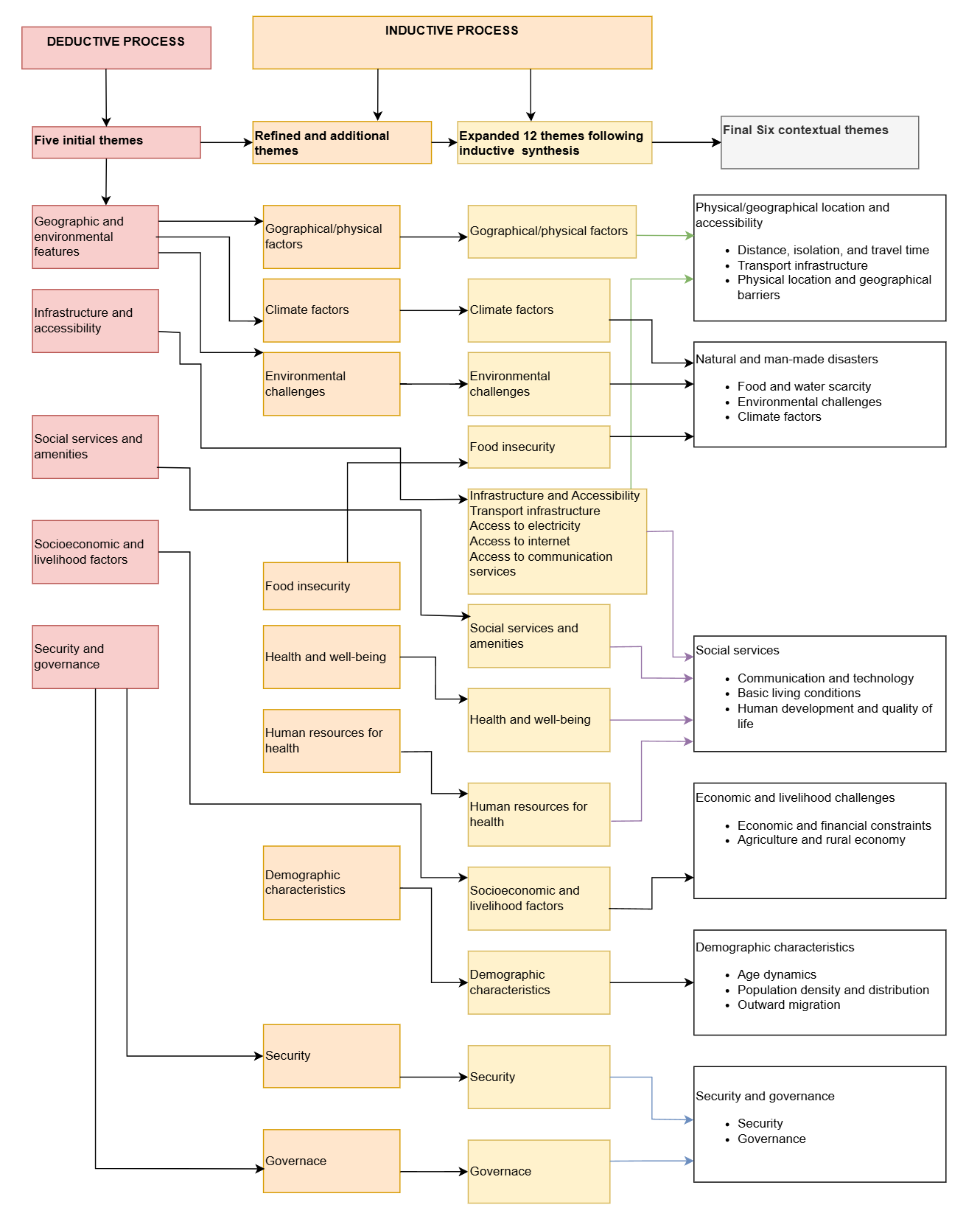
**

Supplement: Supplementary file 4 — Supplementary Material 4: File name: Additional file 4. File format: Doc (Microsoft word). Title of data: Supplementary figure 1: Development of thematic categories for defining hardship areas. Description: Diagram illustrating the process used to develop thematic categories for defining hardship areas [file 12939_2025_2694_MOESM4_ESM.docx]
