# Supplementary material for: Conceptualising hardship areas in Sub-Saharan Africa: a scoping review
Source: Int J Equity Health. 2025 Nov 21;24:326. doi: 10.1186/s12939-025-02694-x (PMC12639685; doi:10.1186/s12939-025-02694-x)
Supplement: Supplementary file 5 — Supplementary Material 5: File name: Additional file 5. File format: Doc (Microsoft word). Title of data: Supplementary Table 3: Overview of Key Characteristics of included studies. Description: Summary table presenting key characteristics of the studies included in the scoping review, including author, year, country, study design, data sources, and definitions or indicators of hardship areas [file 12939_2025_2694_MOESM5_ESM.docx]

**Supplementary Table 3: Overview of Key Characteristics of included studies**

| **Title** | **Type of Publication** | **Region/Country of Study** | **Sub-national area** | **Aim of the study/objective** | **Definition of "Hardship" concept** | **Characteristics of "hardship areas"** |
| --- | --- | --- | --- | --- | --- | --- |
| The good life in rural and urban Senegal: A qualitative and quantitative study. ^1^ | Journal article | Senegal | Dakar and Tessekere | To extend this qualitative work to rural Senegal and combine it with a comparative quantitative study between rural and urban areas | In this rural area of the Sahel, living conditions are harsh. There is no running water or electricity. Ecological conditions greatly limit the crops that can be grown. The first hospital is a two-hour drive away by motorized transport and no one in the area has one except the Great Green Wall Forest rangers. This brief description of the commune of TeÂ´sseÂ´keÂ´reÂ´ and its population illustrates why it can still be described as rural and isolated | Harsh climatic conditions (sahel climate) Unfavourable ecological conditions No Motorized transport No running water No electricity Rural and isolated Limited access to healthcare (2hour drive away) |
| Characteristics of rural areas and their effects on teaching and learning dynamics.^2^ | Journal article | Namibia | Omusati region | The purpose of this study was to establish the factors that characterised rural areas and how these factors influenced teaching and learning activities | To conceptualise ‘rural’ as areas that are geographically distant from towns and cities, making access to essential goods and services difficult. This distance creates adverse living and working conditions, often associated with poverty, poor infrastructure, and social isolation. Rural environments are marked by limited transport due to poor road networks and difficult terrain, restricted access to health, police, and financial services, and inadequate internet connectivity. Social and recreational opportunities are scarce, contributing to low quality of life and challenges for teachers, especially those from urban backgrounds. Rural schools face additional hardships, including long commutes for teachers, lack of staff housing, vulnerability to extreme weather, and limited resources. Overall, rural communities experience harsh conditions, low economic potential, and deprivation of basic services such as clean water, electricity, and proper housing. | Remote and isolated: (distant from town and urban centres) Adverse working and living conditions (absence of essential goods and services clean water, electricity, proper housing and roads infrastructure) Transportation challenges (due to the areas being rocky, sandy and bushy ) Poor road network Adverse working and living conditions (absence of essential goods and services) Aging population Unemployment Poverty (a lack of resources with which to acquire basic goods and services) Low economic potential Limited banking services Limited internet access Extreme weather conditions: Floods  Limited entertainment, sports, and recreational opportunities  Absence of high-level of education |
| Node selection for the integrated sustainable rural development programme in South Africa.^3^ | Journal article | South Africa | South Africa | This paper evaluates the location of the 13 nodes to determine whether the municipalities in these nodes have high levels of poverty and are in need of development. | ISRD Nodes-Population predominantly rural (96.5 per cent). . Low levels of income (per-capita income R114.10 per month). . High population densities (57 people per km2). . High dependency rates (858 youths below 14 years per 1000 people in age category 15 to 65). . High unemployment (only 0.54 per cent of total number employed). . Low level of formal education (only 1.5 per cent have more than 12 years of education). . Only 1.1 per cent receiving an income of more than R51 201 per year | ISRD-Nodes Predominantly rural, low-income levels, high population densities, high dependency rates, high unemployment, low levels of formal education, |
| Equity in public services in Tanzania and Uganda.^4^ | Report-World bank group | Uganda | Uganda | The Note looks in detail at the nature of the problem as it affects education and health services, assesses measures already in place to  tackle inequity, and makes recommendations to address the problem in the immediate as well as the long-term | Government of Uganda has defined HTRS areas as remote or insecure districts that are unable to attract and retain sufficient numbers of motivated staff because of difficult living conditions.  These districts are characterized by: Remoteness caused by natural factors such as rough terrain, mountains or water bodies - or by less developed infrastructure such as a poor road networks or lack of affordable transport. Remoteness in conjunction with other factors may result in intermittent and costly food supplies; poor access to electricity, communications and housing; long distances to work-stations and a lack of social amenities (e.g. safe water, adequate schooling, adequate health facilities, etc.) Insecurity, caused by insurgency or war, limiting the mobility of people, goods and services. Insecure areas are also often characterized by higher prices (including those for transportation) and dilapidated infrastructure. | Remote (Caused by natural factors, rough terrain, mountains or water bodies) Less developed infrastructure (poor road networks or lack of affordable transport Intermittent and costly food supplies Poor access to electricity,  Poor communication and housing Long distances to work-stations Lack of social amenities (e.g. safe water, adequate schooling, adequate health facilities, etc.) Insecurity (insurgency, war) |
| OECD Regional Development Studies Applying the Degree of Urbanisation A Methodological Manual to Define Cities, Towns and Rural Areas for International Comparisons: A Methodological Manual to Define Cities, Towns and Rural Areas for International Comparison.^5^ | OECD report | - | - | - | Research and empirical evidence show that rural areas are characterized by slow dynamics of farm productivity, widespread  income inequality and volatility of agricultural income; considerable outward migration flows to urban areas that result in depopulation of rural areas; a lack of efficient physical, technological and information technology (IT) infrastructures: public and private services that are more costly to provide and more difficult to access than in urban area Some of the main challenges facing rural areas include malnutrition, food insecurity, poverty, limited adequate health and education services, a lack of access to other basic infrastructure and the under-utilisation of labour. Rural areas have particular characteristics that present unique challenges. These include, among others: the dispersion of rural populations; topographical features (terrain and landscapes) that may act as a barrier for the efficient provision of infrastructure; an (over) reliance on the agricultural sector; ensuring that natural resources and environmental quality are protected | Slow farm productivity dynamics  Widespread income inequality and volatility of agricultural income  Outward migration to urban areas  Lack of efficient physical IT infrastructure  Costly and difficult access to public and private services  Malnutrition, food insecurity, and poverty  Limited adequate health and education services  Under-utilisation of labor  Population dispersion  Topographical barriers (terrain and landscape) Over-reliance on agriculture |
| Guidelines on Defining Rural Areas and Compiling Indicators for Development Policy.^6^ | FAO publication | - | - | - | definitions most usually address one or more of three dimensions that characterize differences between urban and rural areas and among rural areas. Sparse settlement reflects the idea that urban areas are those that have the most people and that are most densely settled, while rural areas are more sparsely populated and settled. Land cover is the physical cover on the land including vegetation (either planted or naturally occurring) and any buildings or features constructed by humans. Land cover reflects and determines land use, which is related to the human activities that take place there.  Remoteness affects the opportunities people have to gain access to markets and to public services. It is most often represented by the difficulty of physical travel to places where markets and services are more available | Sparsely populated and settled Remoteness (represented by the difficulty of physical travel to place where markets and services are more available) Land cover (Agriculture) Limited access to markets and services |
| Policy interventions for attraction and retention of female teachers in rural secondary schools: Perspectives of rural educators in Makueni County, Kenya.^7^ | Journal article | Kenya | Makueni | The paper presents findings from the qualitative component of the study. | The shortage is more pronounced in schools in hardship areas. These are poor, remote and geographically isolated rural areas with harsh climatic conditions and poor infrastructure and bereft of any viable economic resources.Evidence suggests that the hardships associated with Arid and Semi-arid Lands makes recruitment, deployment and retention of teachers difficult. Most of the houses in rural markets are of very low quality…… small rental rooms …Available houses were in poor condition … with no electricity and shared amenities… | Poverty Poor infrastructure (Houses in poor condition, with no electricity and shared amenities) Lack of viable economic opportunities Remote Geographical isolated rural Harsh climatic conditions (Arid and semi-arid) |
| Tanzania human resource capacity project. Multisectoral criteria for defining underserved areas.^8^ | Report | Tanzania | Tanzania | - | Physical-geographical  location factors Hard to reach due to natural and physical  obstacles to transport and  communication  Marginal or peripheral location from  district, regional, or national capital (remoteness) High vulnerability to natural calamities  such as floods, droughts, famine,  predators, vermin, and epidemics Basic social services- related factors Lack of reliable and quality health  services and lack of educational facilities (nursery, primary, and secondary)  Lack of reliable public transport   Lack of appropriate housing, electricity,  and safe water supply Lack of banking services, telephone  services, and Internet Inadequate recreational facilities and  amenities | Hard to reach (due to natural and physical obstacles to transport and communication) Remote (marginal or peripheral location from district, regional or national capital) High vulnerability to natural calamities such as floods and drought Limited infrastructure (lack of reliable transport, lack of appropriate housing, electricity, internet, telephone services, banking services) Lack of social services and amenities (inadequate recreational facilities, reliable and quality health services and educational facilities) |
| WHO Guideline on Health Workforce Development, Attraction, Recruitment and Retention in Rural and Remote Areas.^9^ | WHO-report | - | - | - | The definition of a rural area and urban area is generally based on the national characteristics that distinguish them, such as population size and density, administrative criteria and economic structures and features. | Sparse population (small population size and density) Limited economic structures and features (agriculture dependence, small-scale business, high levels of unemployment, seasonal unemployment and underemployment) |
| Increasing access to health workers in remote and rural areas through improved retention: global policy recommendations.^10^ | WHO-report | - | - | - | Each country's own definition for these terms generally takes into account two main elements: the settlement profile (population density, availability of economic structures) and the accessibility from an urban area (distance in kilometres or hour's drive). For the purpose of these recommendations, rural areas are areas that are not urban in nature | Sparse settlement (low population count and density) Limited economic structures (agriculture dependence, small-scale business, high levels of unemployment, seasonal unemployment and underemployment) Distant from urban centres. |
| Reimaging Primary Health Care Workforce in Rural and Underserved Settings.^11^ | Discussion paper (world bank) | LMICs | LMICs | To presents a â€œstart localâ€ health service delivery model, health system design framework and financing models intended to ensure high quality local comprehensive PHC is available and accessible to all. | Rural regions are characterized by low population density  and relatively small communities separated by relatively large distances and local economies that feature farming, fishing, resource extraction including forestry and mining, and/or tourism. Compared to large cities, rural communities typically have limited access to health care with insufficient health workforce to address the health needs of the local population | Low population density Small dispersed communities (small communities dispersed by relatively large distances) Local economy based on agriculture and natural resources (farming, fishing, forestry, mining, tourism) Limited access to healthcare with insufficient health workforce |
| The impact of providing rapid diagnostic malaria tests on fever management in the private retail sector in Ghana: A cluster randomized trial.^12^ | Journal article | Ghana | Dangme West | To examine the impact of providing rapid diagnostic tests for malaria on fever management in private drug retail shops where most poor rural people with fever present, with the aim of reducing current massive overdiagnosis and overtreatment of malaria | The trial was carried out in Dangme West, a rural district with widespread poverty where people live in scattered small communities and are mostly subsistence farmers or fishermen. Vehicular transport is unavailable in many parts of the district making access to formal care difficult | Poo rural Widespread poverty scattered small communities Reliance on subsistence farming and fishing Lack of vehicular transport Difficulty accessing formal healthcare |
| 'You must carry your wheelchair' - barriers to accessing healthcare in a South African rural area.^13^ | Journal article | South Africa | Madwaleni | To explore the challenges faced by people with disabilities in accessing healthcare in Madwaleni, a poor rural Xhosa community in South Africa | Madwaleni is a rural area characterized by rugged hills, rivers, forests, unpaved gravel roads, free running animals, and grass-thatched huts scattered sporadically over the hills. There is a scarcity of sewage systems, running water, and electricity supply to the general Madwaleni community, as these are limited to the hospital and the local hotels. Populations living in rural areas are largely the very young and the elderly, with the employable men and women finding work in the cities | Topographical barriers (rugged hills, rivers, and forests) Poor road condition (unpaved gravel roads dusty during dry season and muddy during rainy season) grass-thatched huts scattered sporadically Largely very young and elderly population Scarcity of sewage systems, running water, and electricity supply in the community only restricted to hospitals and local hotels. Free running animals |
| Access to health in city slum dwellers: The case of Sodom and Gomorrah in Accra, Ghana.^14^ | Journal Article | Ghana | Sodom and Gomorrah | The purpose of the study was to examine health issues in Sodom and Gomorrah in Accra, Ghana, and factors that may be associated with accessing healthcare facilities in the last year. | Sodom and Gomorrah is characterized by poor housing, dirt and squalor, overcrowding and inadequate access to safe and clean water, sanitation and other infrastructure. | Poor quality housing (poor shacks primarily built or held together with old  roofing sheets, plywood and/or cardboard papers.) Dirt and squalor (open gutters are choked with debris, resulting in dirty stagnant ponds and flooding during the rainy season) Inadequate access to safe clean water (no pipe-borne water) Inadequate sanitation (sanitation is generally poor- no well-constructed gutters or drainage systems to allow easy flow  of water) Overcrowding |
| A Public Health Response to a Mudslide in Freetown, Sierra Leone, 2017: Lessons Learnt.^15^ | Journal article | Sierra Leone | Freetown | This concept paper presents an analysis of the events following the mudslide, the response, and the lessons learnt from the response. | On August 14, 2017, a 6-kilometer mudslide occurred in Regent Area, Western Area District of Sierra Leone following a torrential downpour that lasted 3 days. | Mudslide. |
| Addressing fragility through community-based health programmes: Insights from two qualitative case study evaluations in South Sudan and Haiti.^16^ | Journal article | South Sudan | Mayendit county | The aims of the case studies were (1) to deepen the SRCâ€™s understanding of fragility by identifying key factors, dynamics and actors driving overall fragility in the two case study contexts, and (2) to assess the interactions between the health programmes and the fragility context in each case study context to gauge the SRCâ€™s scope of working on fragility, and to provide input for a more holistic SRC health strategy and policy. | Fragile context/situation: Describes a context, which is characterised by weak or unstable institutions, poverty, violence, corruption and political arbitrariness | Weak or unstable institutions, poverty, violence, corruption, political arbitrariness |
| Mozambique's response to cyclone Idai: How collaboration and surveillance with water, sanitation and hygiene (WASH) interventions were used to control a cholera epidemic.^17^ | Journal article | Mozambique | Sofala Province | To examine the strategies implemented to manage a cholera outbreak following Cyclone Idai in Mozambique. | Mozambique ranks among the top three African countries most vulnerable to weather-related events, since the cyclones events in 2019. Cyclone Idai, a category 4 cyclone, struck Mozambique on 14 March 2019 affecting five provinces with heavy rains, severe flooding, and devastating winds leading to cholera epidemic | Cyclone Heavy rains Severe flooding Devastating winds |
| Rural Exposure During Medical Education And Student Preference For Future Practice Location - A Case Of Botswana.^18^ | Journal article | Bostwana | Maun and Mahalapye | To explore the impact of rural training on students’ attitudes towards rural practice | Rural was defined as any location outside of the two largest population centres in Botswana (Gaborone, and Francistown). These two towns represent the major urban centres and are the only places where tertiary healthcare is available and more frequently provided by specialists. This definition was based on a definition by Couper that suggests rural in the health care context relates to the provision of health services to areas outside metropolitan centres where there is not ready access to specialist, intensive and/or high technology care and where resources, both human and material are lacking | Located outside large population centres No ready access to specialist, intensive and high technology care Lack of resource (both human and material) |
| The role of community-based knowledge and local institutions in managing landslides on the slopes of Mount Elgon, Uganda.^19^ | Journal article | Uganda | Nametsi Parish-Bududa District | To explore the forms of community-based knowledge (CBK) and local institutions for disaster preparedness and management in Nametsi Parish, in Bukalasi Sub-County and to assess the extent to which CBK and these institutions can be effective in practice. | The study area has experienced innumerable landslides since 1933. Whereas a few of the landslides have not been disastrous, several others have had far-reaching effects on the livelihoods of people living in the area. This is attributed partly to the geographical setting, which makes the area difficult to access, and partly to socio-economic factors such as a high population growth rate and widespread poverty | Frequent landslides (due to high population growth placing pressure on limited but fertile soils) Difficult to access (due to poorly developed transport network) Widespread poverty (makes access to basic necessities like formal education and healthcare difficult) |
| Midwives' Experiences of Rural Maternal - Newborn Care in Ghana: A Phenomenological Inquiry.^20^ | Dissertation | Ghana | Shai-Osudoku District | This study aimed to understand, unveil the meanings and articulate the experiences of midwives who practice in rural settings in rural Ghana | Rural communities in Ghana are sparsely populated; people farm for both economic and domestic purposes and primarily depend on natural resources such as lakes and rivers for subsistence living. Poor road conditions worsened by the rainy season make travel difficult and hinder emergency referrals. | Sparsely populated Agriculture-based livelihoods (people farm for both economic and domestic purposes) Dependence on natural resources (lakes and rivers for subsistence living) Limited transportation options; Trotro (shared minibuses) Poor road conditions (Bumpy roads with potholes) Weather-related accessibility (poor roads become impassable during the rainy season) Healthcare Access Barriers :Difficult transportation complicates emergency referrals, especially for neonatal and obstetric cases |
| Beyond Crop-Raiding: Unravelling the Broader Impacts of Human-Wildlife Conflict on Rural Communities.^21^ | Journal article | Ghana | Kakum Conservation Area | To examine the impacts of human-wildlife conflict (HWC) in the Kakum Conservation Area (KCA), Ghana. | Human-wildlife conflict (HWC), primarily driven by crop-raiding elephants. Farmers in these rural areas experience significant economic losses, with their livelihoods threatened by the destruction of staple crops leading to food insecurity. | Human-wildlife conflict (occurs when wildlife inflict harm on agriculture, livestock, farm-raised game, and fisheries. This frequently prompts intentional or retaliatory harm to species prioritized for conservation, instigated by individuals within and beyond the boundaries of protected areas Food insecurity |
| Does irrigation enhance and food deficits discourage fertilizer adoption in a risky environment? Evidence from Tigray, Ethiopia.^22^ | Journal article | Ethiopia | Tigray | To investigate the effect of rainfall risk on fertilizer use | The northern Ethiopian highland in general and the Tigray region in particular is a drought prone area where agricultural production risk is prevalent. | Drought-prone area  High agricultural production risk |
| Access to climate change information and support services by the vulnerable groups in semi-arid Kenya for adaptive capacity development.^23^ | Journal article | Kenya | Marigat Division | Identify the dissemination pathways that the vulnerable people in a semi-arid environment perceive most useful for delivering climate information and support services to them | Marigat Division, a semi-arid environment in Kenya experiencing frequent exposure to climate variability-rainfall is highly variable, prolonged droughts with frequent flooding -outbreaks of human and livestock diseases | Semi-arid environment, prolonged droughts, flooding during rains, disease outbreaks. |
| Sugar for the tea: assistance and the state of pastoralism in the Horn of Africa.^24^ | Commentary | Horn of Africa | Kenya, Ethiopia, Somalia | To discusses some of the realities faced by the 20 million pastoralists and the international agencies that deliver emergency and development assistance in the pastoral arc of the Horn of Africa (HOA). | Isolation, poor roads (not many kilometres of all-weather roads), few banks, and outside larger towns,no schools, spotty access to telecommunications, and insecurity make it difficult for donor agencies and non-governmental organizations (NGOs) to implement projects in the horn of Africa which has arid and semi-arid climate. | Isolation Poor roads (Just a few kilometres of all-weather roads which does not cover the entire district) spotty access to telecommunications Insecurity Arid and semi-arid climate |
| How does additional education affect willingness to work in rural remote areas in low-income contexts? An application on health workers in Tanzania.^25^ | Journal article | Tanzania | Tanzania | To shed a light on a potential effect of offering educational opportunities as a strategy to recruit health workers to rural areas. | As a follow-up, they were asked if this place was located more than a three-hour bus ride from the district headquarters, which is our definition of a rural remote area. | Distant from district headquarters ( 3 hour bus ride) |
| Traditional coping strategies to famine among the Keiyo people living in Kerio Valley, Kenya.^26^ | Journal article | Kenya | Kerio Valley | To investigate the perception of famine and traditional coping strategies to famine among the Keiyo people living in Kerio Valley. | Kerio Valley is a semi-arid region that is affected by occasional famine and food shortage | Semi-arid Famine-food shortage |
| Vulnerability to flood-induced public health risks in Sudan.^27^ | Journal article | Sudan | Kassala state | To analyze flood vulnerability vis-a-vis flood-induced health risks and understand the relationship between them to suggest measures to reduce health risks in Sudan. | The study area is part of Gash Delta and the main economic activity is cultivation. The state faces malaria outbreaks, dengue fever and diarrhea with high vectors’ density. Flood in the area is annual with five years frequency of significant damaging events. It results from an overflow of the Gash River during the rainy season. | Agriculture based economy Riverine flood during rainy season |
| Participatory analysis of vulnerability to drought in three agro-pastoral communities in the West African Sahel.^28^ | Journal article | Niger | Fakara, Zermou, Gabi | The specific objective of this study was to assess the strength and limitation of a participatory vulnerability approach using a case study | The region experiences a tropical semi-arid climate, with a single rainy season from June to October, making it highly vulnerable to drought and food insecurity. | Tropical semi-arid climate Drought prone Food insecurity |
| Spatial variability of malnutrition and predictions based on climate change and other causal factors: a case study of North Rift ASAL counties of Kenya.^29^ | Journal article | Kenya | ASAL counties | To determine and model various causal factors of malnutrition in ASAL areas of North Rift Kenya. | Several factors are involved in causation of malnutrition with Arid and Semi-Arid lands greatly affected as a result of erratic weather patterns, droughts, conflicts, poor access to health services and food among others. | Erratic weather patterns Droughts Conflicts Poor access to health services Food insecurity High prevalence of malnutrition |
| A comparative study of community perceptions regarding the role of roads as a poverty alleviation strategy in rural areas.^30^ | Journal article | South Africa | Njane, Ntshaseni | To explore the influence of rural roads on the socio-economic conditions of rural communities. | The Ntshaseni rural community is very isolated and located very far from main roads. The areas terrain is very steep, making it very difficult to travel along the earth roads, which are in very poor condition. Due to the location of the community, rural dwellers have to travel for hours to reach schools and healthcare facilities (IDP, 2014). Furthermore, the areas proximity to the main road makes public transportation expensive and many rural dwellers walk long distances to find transport. A lack of access and mobility has increased the levels of poverty in the area, as rural dwellers do not have access to social and economic opportunities | Very isolated (located very far from main roads)  Steep terrain (making it difficult to travel along earth roads in poor condition)  Poor roads conditions (earth roads impassable during the rainy season)  Limited access to education and healthcare (rural dwellers travel for hours to reach schools and healthcare facilities)  Economic and social exclusion (Limited mobility prevents residents from accessing markets, jobs, and trade opportunities, reinforcing poverty) |
| Environmental associated emotional distress and the dangers of climate change for pastoralist mental health.^31^ | Journal article | Ethiopia | Dullasa Woreda | To present a formative study into the relations between emotion, wellbeing and water security among pastoralist communities in Afar, Ethiopia. | The study was undertaken in Dullassa Woreda within Administrative Zone 3 (Gabi rasu) in the Afar Region, Ethiopia. The region is characterised as semi-arid subjecting the local population to harsh, water-stressed conditions with extreme temperatures exceeding 50 Â°C and receiving less than 200 mm rainfall per annum per year in some areas (Davies and Bennett, 2007). Drought is a frequent occurrence with high variability in rainfall with one main rain season every year between June and September with short showers experienced in December and during March-April. | Semi-arid climate Harsh water-stressed conditions Extreme temperatures (> 50 degrees Celsius) Frequent drought Variable rainfall |
| Trends in extreme temperature and rainfall indices in the semi-arid areas of Western Tigray, Ethiopia.^32^ | Journal article | Ethiopia | Western Tigray | The aim of this study was to analyze the trends in extreme temperature and rainfall in major sesame producing areas in western Tigray using RClimDex software. | The area is characterized as hot to warm semi-arid agro ecology with high rainfall variability ranging 300 mm to 800 mm (MOA 1998), and short length of growing period, erratic rainfall and high daily maximum and minimum temperature variability. The maximum temperature of the study area reaches up to 42°C from April to June, and declines 25-35°C from late June to February | Semi-arid climate  High rainfall variability  Short growing period  Erratic rainfall  Temperature extremes (42 degrees Celsius) |
| Abuja slums: development, causes, waste-related health challenges, government response and way-forward.^33^ | Journal article | Nigeria | Abuja | To ascertain the slums in Abuja and their characteristics, the actual causes of slum development and growth in Abuja, and the waste generation and management situation. | Slum dwellers are often of low socio-economic status. Many of them face high rates of unemployment due to lack of skills and education as well as competitive job markets. Limited job opportunities compel many of them to employ themselves in the informal economy, inside the slum or in developed urban areas near the slum. With poverty and informal economy, slums do not generate tax revenues for the government and therefore tend to get minimal or slow government attention. | Informal economy (high unemployment compels them to employ themselves within slums) Low tax Revenue (the low socioeconomic status and informal economy slums do not generate tax revenues leading to government neglect) |
| Living on the margins: socio-spatial characterization of residential and water deprivations in Lagos informal settlements, Nigeria.^34^ | Journal article | Nigeria | Lagos | To describe the residential and water deprivations of 15 selected slum comÂ­munities in Lagos Nigeria | The Lagos slums are characterised by lack of adequate water infrastructure and sanitation facilities, and poor drainage and road networks.  These settlements are unplanned with clustered buildings, poorly ventilated dwellings and lack of open space, high population, contentious tenure and poorly serviced built environment | Lack of adequate water infrastructure and sanitation facilities Poor drainage, Poor road networks High population Unplanned with clustered buildings, Poor housing (poorly ventilated dwellings),  Lack of open space Poor population contentious tenure  Poorly serviced built environment |
| An assessment of the energy poverty and gender nexus towards clean energy adoption in rural South Africa.^35^ | Journal article | South Africa | Uneletrified rural Limpopo, Mpumalanga, and KwaZulu-Natal. | To study the challenges of gendered energy poverty (GEP) in rural South Africa | Rural households and informal settlements without electricity access are often challenged with the effects of indoor air pollution (IAP) and with the resulting risks to human health and the environment. | Lack of electricity access  Indoor air pollution Gendered energy poverty (effects of energy poverty on women and girls because of their traditional responsibility of providing and using unclean energy fuels for their households) |
| An exploratory analysis of the spatial variation of malaria cases and associated household socio-economic factors in flood-prone areas of Mbire district, Zimbabwe.^36^ | Journal article | Zimbabwe | Mbire district | To examine the spatial variation of malaria and associated socio-demographic factors in flood-prone areas of Mbire district, Zimbabwe | Traditionally flood-prone areas like Mbire District in Zimbabwe, hardship is characterized by exposure to recurrent riverine floods caused by convectional rains, tropical cyclones, and backflows from rivers. The area is entirely rural and drought-prone, with high levels of poverty. Additionally, 79.6% of the population lacks water and sanitation facilities, and 82.6% live in pole and dagga huts, making them highly vulnerable to environmental hazards. Floods not only destroy homes and livelihoods but also contribute to increased malaria outbreaks by creating habitats for vector mosquitoes, exacerbating public health challenges in this malaria-endemic region. Limited economic opportunities, reliance on smallholder farming. | High poverty indices poverty Local economy based on smallholder farming Recurrent riverine floods (caused by convectional rains, tropical cyclones, and backflows from rivers) Drought-prone Malaria endemic 79.6% of the population lacks water and sanitation facilities, increasing health risks. Poor housing (82.6% of the population lives in pole and dagga huts, making them highly vulnerable to extreme weather conditions) |
| Retention and job satisfaction among rural primary school teachers in Malawi.^37^ | Journal article | Malawi | Mzuzu and Lilongwe district | To investigate the predictors of teacher retention and job satisfaction in rural primary schools in Malawi, using Mason and Matas’ four-capital model of teacher retention. | In the Malawian context, a rural community is operationally defined as a geographical area with a population of less than 5000 people. Globally, such communities are synonymous with a lack of essential services, such electricity, hospitals and schools, and limited economic opportunities | Population less than 5000 people Lack of essential services (such as electricity, hospitals, and schools, and limited economic opportunities) |
| Four years trends of malaria admissions in rural and urban Kandi health facilities in northeast of Benin Republic.^38^ | Journal article | Benin. | Kandi, northeast of Benin. | To assess trend of malaria admissions between 2009 and 2012 in rural and urban health facilities of the district of Kandi, a very arid area with particularly severe drought lasting up to 6 months | In North Benin, the district of Kandi is a very arid area with particularly severe drought lasting 6 months during every year which significant cases of malaria are detected in health facilities. | Arid climate Very high temperatures (45 degrees Celsius) Prolonged severe droughts High malaria burden |
| Transforming a local food system to address food and nutrition insecurity in an urban informal settlement area: a study in Umlazi township in Durban, South Africa.^39^ | Journal article | South Africa | Umlazi-Durban | To contribute to the food security narrative by determining factors that can support the transformation of a food production system within the demarcated geographical setting | Informal settlements in South Africa are facing diverse challenges such as land inaccessibility for food production, poverty, unemployment, malnutrition, and climate change (flooding) attributing to food insecurity. | Land inaccessibility for food production Malnutrition Climate change (flooding) Poverty Unemployment Food insecurity-climate change induced |
| Rapid response mechanism in conflict-affected settings of Cameroon: lessons learned from a multisector intervention for internally displaced persons.^40^ | Journal article | Cameroon | Ekondo Titi | To reflect on our experience and lessons learned for future rapid response mechanism interventions in humanitarian settings | Ekondo Titi is a health district in Ndian Division, the closest accessible town to Ekondo Titi is 60 kilometers away. It is one of the districts highly affected by the armed conflict and with extremely bad roads (Ekondo Titi has always had seasonal roads even prior to the crisis. In the rainy seasons the access is difficult with transportation cost going as high as $9 USD and took like three to seven hours to travel from Kumba to Ekondo Titi. In the dry season the roads were maintained regularly, and the transportation cost was at $5 USD and usually took an hour to get to Ekondo titi) limited communication networks, limited access to electricity, low literacy levels and high presence of non-state armed groups | Remote (closet town is 60 kilometres away) Seasonal roads(In the rainy seasons the access is difficult with transportation cost going as high as $9 USD and takes like three to seven hours to reach closest town) Limited communication networks Limited access to electricity  Low literacy levels  High presence of non-state armed groups |
| The effect of the anglophone crisis on youth sexual and reproductive health in the northwest region of Cameroon: a qualitative study.^41^ | Journal article | Cameroon | Bamenda-Northwest | To describe the effects of the crisis on adolescent SRH and identify stakeholder recommendations for strategies to address the SRH of youth in their crisis-impacted communities. | The ongoing armed conflict in the two English-speaking regions of Cameroon, named the Anglophone crisis has had a deleterious impact on the health and wellbeing of the populations inhabiting these regions and led to internal dispalcements | Armed conflict Conflict induced population displacement Disruption of health services (negative impact on health and wellbeing) |
| Effects of climate change on food production in semi-arid areas: a case study of Uzumba Maramba Pfungwe District, Zimbabwe.^42^ | Journal article | Zimbabwe | Uzumba Maramba Pfungwe | The study therefore sought to provide evidence on the impacts of climate shocks on agricultural food production systems and their intersection with food security. | Uzumba Maramba Pfungwe is a semi-arid district that generally receives low rainfall with the bulk of the district lying in agro-ecological region. The district is not spared from climatic hazards such as droughts, dry spells and extreme temperatures. | Semi-arid climate Low rainfall Climate hazard (drought, dry spells, extreme temperatures) |
| Climate change in semi-arid Malawi: Perceptions, adaptation strategies and water governance.^43^ | Journal article | Malawi | Mphampha-Chikwawa | To assess community’s perception of a changing climate against empirical evidence, determine their local adaptive measures, evaluate the potential of irrigated agriculture as an adaptive measure in household food security and challenges over access to available water resources. | The climate of Mphampha Village is characterized by hot, dry conditions with low annual rainfall and number of rainy days. The normal rainy season lasts from November to March. However, part of the rainy season is characterised by very strong winds which destroy crops and houses | Hot and dry conditions Low annual rainfall Strong winds which damage crops and houses. |
| Can timely vector control interventions triggered by atypical environmental conditions prevent malaria epidemics? A case-study from Wajir County, Kenya.^44^ | Journal article | Kenya | Wajir county | To describe in Wajir town the environmental conditions, the scope and timing of vector-control interventions and the associated resulting burden of malaria at two time periods (1996â€“1998 and 2005â€“2007 | Wajir county ranges from very arid in the north, to semi-arid in the south. The county experiences the Sahel climate characterized by long dry spells and two rainy seasons. These desert fringe conditions create seasonal malaria transmission during, and immediately following, the two rainy seasons | Arid and semi-arid Sahel climate (long dry spells and two rainy seasons) Seasonal malaria transmission |
| Preliminary studies on membrane filtration for the production of potable water: a case of Tshaanda rural village in South Africa.^45^ | Journal article | South Africa | Tshaanda | To ascertain the use of ultrafiltration membrane for the provision of potable water in Tshaanda. | In Tshaanda, people get water from an unprotected spring, which is the only source of water supply in the area. The village is impoverished and excluded from the mainstream water network in Vhembe and it is unlikely that the village will be connected to the mainstream water infrastructure in the future because the mountains that are surrounding it make Tshaanda difficult to access | Difficult to access (area is surrounded by mountains) Lack of safe water infrastructure (unprotected spring as the main source of water supply) Poverty |
| A Retrospective Cross Sectional Study of the Effectiveness of a Project in Improving Infant Health in Bwindi, South Western Uganda.^46^ | Journal article | Uganda | Bwindi-Southwestern | The aim of this study was to determine whether the inputs from the HEAL project, the nurse visits antenatally and/or postnatally, combined with CHV visits to households, had an effect on the health (as defined by nutrition status, completion of immunizations by 9 months and where the child was delivered) or mortality of infants born in those households within the three sub counties of Kayonza, Kanyantorogo and Mpungu in Kanungu district, Uganda, between January 2015 and December 2016 | Hard to reach households were defined as being in a village more than 5 km from a health facility, whose access was up the side of a ridge over 500 m high, and which could not be accessed using a motorcycle because of the lack of a passable track | Limited access to healthcare- Villages are located more than 5 km from a health facility. Difficult Terrain (access was up the side of a ridge over 500 m high) Poor Infrastructure (no passable track for motorcycles, causing inaccessibility.) |
| Improving prompt access to malaria diagnostics and treatment in rural remote areas using financial benefit for community health workers in Kilosa district, Tanzania.^47^ | Journal article | Tanzania | Kilosa distrcit | To test the effectiveness of a financial benefit approach to motivate CHWs to improve prompt access to malaria treatment. | Rural remote areas are communities located 5 km from a nearby health facility or a drug shop. | Remote > 5km from nearby health facility |
| Guiding developments in flood-prone areas: Challenges and opportunities in Dire Dawa city, Ethiopia.^48^ | Journal article | Ethiopia | Dire Dawa | To assess guidance of developments in Dire Dawa town in Ethiopia, specifically on describing the severity of floods, determining the benefits, identifying the key challenges and determining the opportunities related to guiding developments in flood-prone areas | Floods in Dire Dawa are occurring more frequently than before and this is attributed to climate change, among other reasons. | Flooding |
| Seasonal malaria chemoprevention: successes and missed opportunities.^49^ | Opinion-Malaria Journal | Sahel | Sahel | - | Hard-to-reach areas, both because of poor infrastructure and because of political instability and access is difficult especially during the rainy season. | Hard to reach Poor infrastructure (poor road/transport network) Political instability Weather related inaccessibility |
| Citizen feedback in a fragile setting: social accountability interventions in the primary healthcare sector in Sierra Leone.^50^ | Journal article | Sierra Leone | Sierra Leone | To explore the utility of social accountability interventions in fragile environments. | Fragile and conflict-affected states are frequently characterised by their inability to fulfil three core governance functions: provision of security, effective delivery of basic public goods and services, and managing political participation and accountability | Insecurity Lack of political participation and accountability Inability to deliver basic public goods and services |
| Epidemiology of soil transmitted helminth and Strongyloides stercoralis infections in remote rural villages of Ranomafana National Park, Madagascar.^51^ | Journal article | Madagascar | Ifanadiana | The objective of this study is to provide a much-needed report on the prevalence rates of STH and S. stercoralis infections and to identify the risk factors among a neglected population living in remote villages neighboring RNP in Madagascar | The participating villages were found to be traditional, rural, and heavily reliant on subsistence agriculture. Village sanitation is poor, with lack of water infrastructure and commonly practiced indiscriminate defecation. All twelve villages cannot be reached by motor vehicle travel since there are no roads or paved paths | Traditional and rural Heavy reliance on subsistence agriculture Poor sanitation (commonly practice indiscriminate defecation) Lack of water infrastructure (no access to clean water, rather utilized freshwater rivers for their basic needs) No roads or paved paths |
| Rainfall-Induced Landslide Prediction Using Machine Learning Models: The Case of Ngororero District, Rwanda.^52^ | Journal article | Rwanda | Ngororero District | To improve the performance of landslide prediction models and minimise incorrect predictions and develop early warning systems using machine learning techniques | Landslides and floods are the common natural disasters that strike the northwestern provinces of Rwanda due to its topographical, geological features and climatic profile. 42% of areas are classified as moderate to very high susceptible areas to landslides. | Steep slope Landslides Floods |
| No safety net in the face of climate change: The case of pastoralists in Kunene Region, Namibia.^53^ | Journal article | Namibia | Kunene | This study aims to collect this information in order to explore the use of EbA to help pastoralists adapt to climate change | In the Kunene region, particularly in Epupa Constituency, is characterized by severe climatic challenges (sporadic rainfall, arid conditions, dry spells, and frequent droughts), difficult terrain (semi-arid land transitioning to desert), and climate change impacts (persistent droughts and livestock loss). The region is economically dependent on farming, with high rates of poverty (51% severely poor) and underdevelopment (Epupa is the least developed constituency in the area). The literacy rate is low (29%), and education is severely limited, with 70% of children never attending school. | Climatic challenges (sporadic rainfall, arid conditions, dry spells, and frequent droughts) Difficult terrain (semi-arid transitioning to desert) Economically dependent on farming High poverty rates 51% (failure to support own livelihood) Underdeveloped Low literacy levels (29%) Severely limited education(70% of children never attend school) |
| Community participation in health research: an ethnography from rural Swaziland.^54^ | Journal article | Swaziland | Rural Swazi | Our aim is to enhance knowledge about participation in research by detailing what community co- researchers actually did in PHR in rural Swaziland, and discussing, with reference to related articles reporting the outcomes of the PHR process, how their participation influenced empowerment and research rigour. | The community in which we conducted our study was isolated by mountainous terrain and poor transport infrastructure, and marginalized by poverty, lack of basic infrastructure and limited income-generating opportunities | Isolation due to mountainous terrain and poor transport infrastructure Poverty Lack of basic infrastructure (lack of water) Limited income-generating opportunities |
| Seasonal Variation of Household Food Insecurity and Household Dietary Diversity on Wasting and Stunting among Young Children in A Drought Prone Area in South Ethiopia: A Cohort Study.^55^ | Journal article | Ethiopia | Sidama | To evaluate seasonal patterns of household food insecurity, dietary diversity, and household  characteristics on wasting and stunting among children in households followed for 1 year in the drought-prone areas of Sidama, Ethiopia | The study took place in an area of ecological degradation, high population pressure, and repeated droughts and recurrent food shortages | Ecological degradation High population pressure Repeated droughts  Recurrent food shortages |
| Spatial Variation of Child Stunting and Maternal Malnutrition after Controlling for Known Risk Factors in a Drought-Prone Rural Community in Southern Ethiopia.^56^ | Journal article | Ethiopia | Boricha | The aim of this study was to assess whether child stunting and maternal malnutrition were spatially clustered in drought-prone areas after controlling for previously known risk factors of malnutrition | Boricha district is a drought prone area with more than 90% of the population living in rural areas and most people make their living directly from subsistence farming and livestock rearing. Malnutrition is the major health problem | Drought prone High malnutrition prevalence Economically dependent on farming and livestock rearing 90% of the population lives in rural areas |
| The Effects of Conflicts and Self-Reported Insecurity on Maternal Healthcare Utilisation and Children Health Outcomes in the Democratic Republic of Congo (DRC).^57^ | Journal article | Congo (DRC) | Congo (DRC) | To explore the relationship between self-reported insecurity of mothers and maternal health-seeking behaviours and diseases in children in the DRC | The Democratic Republic of Congo (DRC) has experienced political unrest, civil insecurity, and military disputes, resulting in extreme poverty and a severely impaired healthcare system | Political unrest Civil insecurity Military disputes Extreme poverty Impaired healthcare system |
| Climate change impacts on water sustainability of South African crop production.^58^ | Journal article | South Africa | South Africa | To evaluate the sustainability of water, use for crop production in South Africa by analysing current and future water consumption patterns under different climate change scenarios. | Challenges faced by rural farming communities due to climatic, environmental, and economic stressors that limit their ability to produce food and maintain livelihoods. This includes water scarcity, unsustainable irrigation, low agricultural yields, and vulnerability to climate change. | Climate change- induced water scarcity Unsustainable irrigation Low agricultural yields Soil moisture depletion |
| Destruction, disruption and disaster: Sudan's health system amidst armed conflict.^59^ | Grey-comment | Sudan | Khartoum, North Kordofan, Darfur and River Nile states. | To examine the destruction, disruption, and disastrous consequences inflicted upon Sudan’s health system. | Eruption of armed clashes on April 15th between the Sudanese Armed Forces (SAF) and the Rapid Support Forces (RSF) in the capital city of Khartoum, North Kordofan, Darfur and River Nile states. This has caused destruction of health infrastructure and disruption of service delivery | Armed conflict Destruction of health infrastructure Disruption of service delivery |
| Tungiasis Stigma and Control Practices in a Hyperendemic Region in Northeastern Uganda.^60^ | Journal article | Uganda | Napak District | To investigate local attitudes and control practices regarding tungiasis, with a focus on stigma. | Karamoja has a long history of animal raids and ethnic conflict and has long been a marginalized region in Uganda with widespread poverty. Houses in the study area were predominantly made of sticks with grass roofs and earthen floors, which were sometimes smeared with cow dung to harden and smoothen the surface. Living conditions in the study area are generally very poor, and hunger and malnourishment are common. Access to water was limited, as the few existing boreholes and shared water taps were located at distances up to 3 km from people's homes and were prone to breaking. The local population had very limited access to formal medical care, as health units were understaffed and located far away from the villages. | Limited access to clean water (few existing boreholes and shared water taps were located at distances up to 3 km from people's homes) Substandard housing (houses made of sticks with grass roofs and earthen floors, sometimes smeared with cow dung to harden and smoothen the surface) Limited access to formal medical care (health units were understaffed and located far away from the villages) Insecurity (animal raids and ethnic conflict) Hunger Widespread poverty (living conditions were generally very poor) Tungiasis endemic |
| An integrated geotechnical and geophysical investigation of landslide in Chira town, Ethiopia.^61^ | Journal article | Ethiopia | Oromia region-Chira | The study aimed to conduct an integrated geotechnical and geophysical investigation of landslides in Chira town, Oromia region of Ethiopia, to provide a more comprehensive understanding of the factors contributing to landslides in the region. | The study area is characterized by a rugged volcanic mountainous terrain comprising of high to low relief hills making it prone to landslides. | Rugged volcanic mountainous terrain High and low relief hills Landslides |
| Factors promoting and hindering sporting success among South African former Olympians from historically disadvantaged areas.^62^ | Journal article | South Africa | Xaba and Malindi | To investigate the enablers and/or barriers to sporting success among South African former Olympians from HDAs using the SPLISS framework. | Historically disadvantaged areas (HDAs) in this study referred to areas that were underfunded and underdeveloped during the apartheid era, whereby the legacy of underdevelopment persists to date. These areas include townships, farms, and rural areas (as well as villages) Xaba and Malindi highlight HDAs as historical settlements characterised by poor socioeconomic conditions, including poor infrastructure development, and most were designated as living areas for black people. | Underfunded and underdeveloped Poor socioeconomic conditions Poor infrastructure development (Lack of transport system, sports infrastructure recreational facilities are non-existent or inadequate) Segregated settlement (designated as living areas for black people) |
| Community participation and childhood immunization coverage: A comparative study of rural and urban communities of Bayelsa State, south-south Nigeria.^63^ | Journal article | Nigeria | Ondewari | This study was to assess the possible effects of greater community participation on immunization coverage, by comparing the immunization coverage in a rural community with a functional community health committee, with an urban community, with no distinct community structure. | The study was carried out in Ondewari, a rural, riverine community, Rural and riverine communities like Ondewari are difficult to live and work in, because of the near absence of social amenities, | Rural community  Riverine setting  Difficult living and working conditions (Near absence of social amenities) |
| A system dynamics approach for understanding community resilience to disaster risk.^64^ | Journal article | South Africa | Western Cape | To identify the mechanisms influencing the level of understanding of Community Resilience and to use these as a basis for recommendations for reducing disaster Risk. | The Western Cape is a dynamic province that is disaster-prone, particularly the vulnerable urban communities in and around its environs. Such communities are more vulnerable to wildfire, flooding, pandemic, natural and human-made hazards because of poverty and, consequently, poor living conditions such as overcrowding and non-understanding of community resilience. The Phola Park community is exposed to flood, wildfire and other DRs. This area consists totally of shacks (houses built by hand with available materials) that are frequently prone to disasters because of inadequate drainage system, the lack of housing and general facilities such as toilets and water taps and overcrowding. | High exposure to multiple hazards such as wildfire, flooding Poverty Overcrowding Lack of housing (shacks-houses built by hand with available materials) Lack of general facilities such as toilets and water taps Inadequate drainage system (making it frequently prone to disasters) |
| Mobile consulting as an option for delivering healthcare services in low-resource settings in low- and middle-income countries: A mixed-methods study.^65^ | Journal article | Tanzania, Kenya, Nigeria | Ulanga district | To review current evidence for mConsulting in LMIC contexts; and engaging with people living and providing healthcare in low-resource settings in Pakistan, Tanzania, Kenya, Nigeria and Bangladesh | Remote rural site TZ1, Ulanga District, Tanzania: Area size (24,460 km2), population (265,203),71 area density (11/km2), predominantly rural population (90%). Public sector health facilities in the district include one district hospital (serving villages up to 80 km away), two health centres (serving villages up to 40 km away) and 16 dispensaries. There are approximately six private dispensaries and clinics within the district. The district hospital is located in one of the study villages. One of the study villages has no health facility but is 6 km from the district hospital/40 km from the nearest health centre. The district has unpaved roads, and residents mostly use bicycles, motorbikes and public transport. Housing is mixed, comprising brick (47%) and mud (24%) structures, with most using iron sheet roofing (74%) and earth/sand flooring (67%) or cement (31%). Three-quarters of all households/88% of rural households have no access to electricity. Three-quarters have access to clean piped water, while the remaining 25% get water from wells and rivers. More than half the population is 18 years or younger, while 6% are aged above 60 years. Overall literacy is 72% (men 76%, women 69%). Almost all (98%) of the working population is self-employed and economically reliant on subsistence farming, fishing and mining.  urban slum Access to clean water is limited and sanitation is poor, leading to frequent outbreaks of cholera and other infectious diseases. Access to electricity is poor with some dwellings having unregulated connections from the main national grid | Remote rural (one district hospital serving villages up to 80 km away) Sparsely populated area  Limited access to healthcare (a single hospital serving people located 80 km away) Limited access to electricity (88% of households have no electricity)  Urban slum Limited access to clean water and sanitation leading to frequent cholera outbreaks Unregulated electricity connections from the main grid |
| Water Insecurity, Water Borrowing, and Psychosocial Stress Among Daasanach Pastoralists in Northern Kenya.^66^ | Journal article | Kenya | Daasanach | This paper aims to fill these gaps by examining the water insecurity experiences, water-sharing practices and psychosocial stress of Daasanach pastoralists  in northern Kenya. | In this article, hardship is defined by resource scarcity, restricted mobility, conflict over resources, environmental stressors, and external socio-political changes that affect the livelihood and well-being of the Daasanach people in Northern Kenya | Climate change induced -Resource scarcity Resource-based conflict (increased conflict over limited resources) External socio-political changes (restricting mobility) |
| Adaptive capacity to reduce disaster risks in informal settlements.^67^ | Journal article | South Africa | eMalahleni Local Municipality | To recommend strategies to enhance adaptive capacity and reduce disaster risks in informal settlements in the eLM. | These settlements are marked by overcrowding, looming threat of eviction, inadequate infrastructure including poorly constructed roads and insecure  housing lacking access to essential services such as water and sanitation, thus exacerbating residents ‘vulnerability to life-threatening diseases  These informal settlements are exposed to a number of hazards, including underground fires, air and water pollution, sinkholes, abandoned mining areas and acid  mining drainage | Exposure to hazards such as underground fires, air and water pollution, sinkholes, abandoned mining areas and acid mining drainage Looming threat to eviction Poorly constructed roads Increased vulnerability to life-threatening diseases. |
| Hunger in the shadow of conflict: analyzing malnutrition and humanitarian challenges in Sudan.^68^ | Journal article | Sudan | Khartoum | Commentary on the effects of conflict on malnutrition and other humanitarian challenges in Sudan | Amidst ongoing conflict, Sudan faces a severe economic crisis characterized by hyperinflation, a weakening currency, and disrupted trade routes, which have compounded pre-existing challenges. These economic disruptions have significantly impaired food production and distribution systems, leading to widespread food insecurity | Conflict Hyperinflation Weakening currency Disrupted trade routes Food insecurity |
| Refugee settlements are highly exposed to extreme weather conditions.^69^ | Journal article | Kenya, Uganda, Tanzania, Rwanda, Sudan, Ethiopia | location of refugee camps | To the study the exposure of the 20 largest refugee settlements worldwide to extreme variations in climatic conditions. | Within refugee-hosting countries, refugee settlements are frequently located in isolated and remote areas, characterized by poor-quality land and harsh climatic conditions. | Isolated and remote Poor-quality land Harsh climatic conditions (high temperature and low levels of rainfall compared to national averages. |
| Armed conflicts have an impact on the spread of tuberculosis: the case of the Somali Regional State of Ethiopia.^70^ | Journal article | Ethiopia | Jigjiga,Shinile zones of Somali Region State | To examine the role of conflict in a regional TB epidemic. | The region is characterized by longstanding conflict between government forces and local armed rebel forces, armed conflicts stall disease control programs through distraction of health system, interruption of patients’ ability to seek health care, and the diversion of economic resources to military ends rather than health needs As a result, people in the region are not only exceedingly poor but also bear a disproportionately high incidence of TB | Conflict and insecurity Restricted movement Distraction of health system High TB incidence |
| Environmental justice in South Africa: the dilemma of informal settlement residents.^71^ | Journal article | South Africa | Kosmos informal settlement | To reveal human experiences, and their individual opinions in relation to the identified and targeted human activities. | Persistent socio-environmental disadvantage experienced by marginalized communities in informal settlements, characterized by limited access to clean water, sanitation, waste management, and energy, compounded by pollution, environmental degradation, and exclusion from decision-making processes affecting their living conditions | Proximity-based environmental injustice Forced relocation and legal struggles Segregated waste management services Social exclusion in decision-making Firewood dependency and deforestation in an urban Setting |
| Structure and management of tuberculosis control programs in fragile states--Afghanistan, DR Congo, Haiti, Somalia.^72^ | Journal article | Congo (DRC), Somalia | None described (National level assessment) | To share lessons learned on public health and TB control programs in fragile states | Health care delivery is particularly problematic in fragile states often connected with increased incidence of communicable diseases, among them tuberculosis. | Fragile Collapse of services thus high TB burden |
| The experiences of people living with HIV/AIDS and of their direct informal caregivers in a resource-poor setting.^73^ | Journal article | South Africa | Eastern cape | To explore and describe the challenges faced by PLHIV and their DICGs in two remote South African villages, and their ability to cope with the illness given the scarce social and economic resources within their communities | The isolated rural townships of the Eastern Cape, South Africa, experience severe economic and health hardships, characterized by high poverty rates, unemployment, and a high prevalence of HIV/AIDS. The province has the highest poverty rate in the country (68.7%), with rural poverty reaching 82.2%, compared to 42.1% in urban areas. | Isolated rural High poverty rate Unemployment High prevalence of HIV/AIDS |
| Improving health workforce recruitment and retention in rural and remote regions of Nigeria.^74^ | Personal View | Nigeria | Nigeria | To analyse the factors hindering the recruitment and retention of health workers in rural and remote areas of Nigeria and propose sustainable strategies to improve the rural health workforce. | Spartan living conditions in rural and remote areas include open defecation, severely limited access to electricity, primitive social amenities, chronic poverty, poor quality educational or communication facilities, fragile health systems and the inadequacy of potable water characterise the rural regions of Nigeria16 | Open defecation primitive social amenities severely limited access to electricity,  chronic poverty Poor quality educational and communication facilities fragile health systems Inadequate portable water |
| East Africa's pastoralist emergency: Is climate change the straw that breaks the camel's back?^75^ | Journal article | Kenya | Turkana | To examine the livelihood strategies of the Turkana and several poverty reduction programme | Vulnerability experienced by pastoralist communities due to environmental degradation, climate change-induced droughts, resource scarcity, economic marginalization, and violent conflict over diminishing resources, leading to food insecurity, displacement, and dependency on humanitarian aid. | Climate change - induced resource scarcity Drought Displacement Resource-based conflict Dependency on aid Food insecurity Insecurity (cattle raiding) |
| Willingness to work in rural areas and the role of intrinsic versus extrinsic professional motivations - a survey of medical students in Ghana.^76^ | Journal article | Ghana | Ghana | This paper analyzes the effect of extrinsic versus intrinsic motivational factors on stated willingness to accept postings to rural under-served areas in Ghana. | We defined deprived area as a rural area that is distant from the big cities with few social amenities such as schools, roads, pipe-borne water, etc.as per Ministry of Health definition. | Rural area Distant from big cities Few social amenities such as schools, roads, pipe-borne water |
| Seasonal and geographic differences in treatment-seeking and household cost of febrile illness among children in Malawi.^77^ | Journal article | Malawi | Chikhwawa | The aim was to understand how physical barriers for access to health care and the consequent expenditure incurred by households influence utilization of health facilities. | The Malawi government has sought to bring care nearer to patients by establishing a community Health Worker (CHW) scheme in some villages that have been defined as hard to reach (HTR) either because they are more than 8 km from a public health facility or have reduced accessibility due to rivers or hills. | Hard to reach  Distant to health facilities (>8km) Reduced accessibility Limited access to health facilities |
| Factors associated with utilization of community health workers in improving access to malaria treatment among children in Kenya.^78^ | Journal article | Kenya | Lamu, Malindi | To examine factors associated with utilization of CHWs in improving access to malaria treatment among children under five years of age by women caregivers in two malaria endemic districts in Kenya. | The study was conducted in Malindi and Lamu districts in the Coast Province of Kenya. The region is characterized by a low level of access to health facilities for much of the population due to difficult geographical barriers, semi-arid climate and prevailing high level of poverty. Many villages are located far from main access roads with terrain that is impassable by motor vehicles, including some areas located across rivers or swamps. Lamu district includes several islands. During the rainy season, widespread flooding and wild animals from neighbouring game parks further aggravate inaccessibility | Geographical barriers: difficult terrain, impassable roads, and village far from main access Climate challenges: semi-arid and seasonal flooding that worsen accessibility Poverty impacting service access Inaccessibility: Some areas are across rivers, swamps, or islands Presence of wild animals |
| Using incentives to attract nurses to remote areas of Tanzania: a contingent valuation study.^79^ | Journal article | Tanzania | Tanzania | To analyze how financial incentives (salary top-ups) and non-financial incentives (housing and education) affect nurses' willingness to work in remote areas of Tanzania and how the magnitude of the incentives needed to attract health workers varies with the nurses' geographic origin and their intrinsic motivation. | Place: More than 500km away from Dar es Salaam, more than 200km away from the nearest regional capital and ~50-100km away from the nearest district headquarter. Availability of schools: School available, but only public with shortage of essential inputs. Transport and communication facilities: Not reliable Utilities (e.g. clean tap water and electricity) Water available, but electricity not guaranteed Recreational facilities: Mostly not available, and if available they are of very poor quality | Distant to service centers Poor educational facilities (shortage of essential inputs in schools) Unreliable transport and communication Limited electricity access Poor quality recreational facilities. |
| Lessons learned from setting up the Nahuche Health and Demographic Surveillance System in the resource-constrained context of northern Nigeria.^80^ | Journal article | Nigeria | Zamfara | This paper describes the steps needed to establish a population health surveillance site in order to share the lessons learned from our experience launching the Nahuche Health and Demographic Surveillance System (HDSS) in a relatively isolated, rural district in Zamfara | In the Nahuche HDSS study site has a typical Sahelian climate with temperature reaching a high 38 degrees celcius, farming is the most common economic and subsistence activity. High unemployment catalyst for temporary labour migration among men. The standard of living is quite low, with hardly any families owning cars or televisions, standard signs of improved living standards. Infrastructure remains deficient with no access to an electricity grid and dependence on local generators. There is no community sanitation system, and most families are depending on latrines and wells or boreholes for their water | Rural and isolated (the site is 32 km from the state capital)  Reliance on subsistence farming as a major economic activity High unemployment Low living standards (hardly any families own cars or televisions-standard signs of improved living standards) No access to electricity grid Lack of community sanitation system |
| Impact of an interprofessional education program on developing skilled graduates well-equipped to practise in rural and underserved areas.^81^ | Journal article | South Africa | South Africa | To investigate the important components of supervision, such as supervision ratios and relationships, feedback response and time/attention allocated to students. | The divide between rural and urban South Africa is indicated by research as characterised by poverty, limited access to resources and a lack of infrastructure in rural areas | Poverty Limited access to resources  Lack of infrastructure (computers and internet) |
| Factors affecting motivation and retention of primary health care workers in three disparate regions in Kenya.^82^ | Journal article | Kenya | Turkana | To investigated factors influencing motivation and retention of HCWs at primary health care facilities in three different settings in Kenya - the remote area of Turkana, the relatively accessible region of Machakos, and the disadvantaged informal urban settlement of Kibera in Nairobi. | The remote and arid Turkana is a county located in the north of Kenya and a hardship area, whereby hardship area is defined to mean a location where living is relatively harder, for example due to being a very dry region and hence inadequate water, or a region of conflict implying restricted movement. | Very dry Remote Inadequate water  conflict Restricted movement |
| The Etiology, risk factors, and interactions of enteric infections and malnutrition and the consequences for child health and development study (MAL-ED): Description of the Tanzanian site.^83^ | Journal article | Tanzania | Haydom (North-central Tanzania) | To contextualize the Haydom, Tanzania (TZH) site, report on how the site was selected, describe the data that informed the cohort recruitment strategy, and describe features of this rural sub-Saharan Africa | Haydom is situated 300km from Arusha, the nearest urban centre. It is a remote rural area with poor road infrastructure in a highland setting. The population of approximately 20,000 inhabitants is primarily dependent on subsistence agriculture. Socioeconomic indicators are poor, with essentially no household having access to electricity, piped water, or improved sanitary facilities (compared with 14%, 7%, and 12%, respectively, reported nationally) | Remote (300 km away from nearest urban centre) Poor road infrastructure in a highland setting Dependent on subsistence agriculture  No household has access to electricity, piped water or improved sanitary facilities |
| The effect of enhanced public -private partnerships on Maternal, Newborn and child Health Services and outcomes in Nairobi-Kenya: The PAMANECH quasi-experimental research protocol.^84^ | Journal article | Kenya | Nairobi | The aim of the project is to determine whether strengthening the private sector with support from the public sector for the delivery of quality health services for slum residents has an impact on Maternal, Newborn, and Child Health (MNCH) outcomes. | Viwandani and Korogocho settlements are together home to approximately 70 000 residents and, like other slums, are characterised by poverty, poor coverage of social services and poor MNCH outcomes | Poverty Poor coverage of social services such as healthcare , water and sanitation Poor maternal, neonatal, and child health outcomes |
| Female health workers at the doorstep: a pilot of community-based maternal, newborn, and child health service delivery in northern Nigeria.^85^ | Journal article | Nigeria | Kadawawa | To explore the feasibility of deploying resident female Community Health Extension Workers (CHEWs) to rural areas to provide essential maternal, newborn, and child health services. | Located approximately 36 km from the LGA headquarters, Kadawawa is a remote, rural community with a population of just under 16,000 people spread out across several hamlets. The terrain of the community is rough and barely accessible during the rainy season. Commercial vehicles and motorbikes get to Kadawawa once a week on market days. When health workers have to travel to and from the community, this is feasible only on market days, which fall on Fridays. | Remote (> 36km from LGA headquarters) Sparse population Rough terrain inaccessible during rainy season Unreliable transport (commercial vehicles and motorbikes get to the area once a week on market days) |
| Incentives for non-physician health professionals to work in the rural and remote areas of Mozambique--a discrete choice experiment for eliciting job preferences.^86^ | Journal article | Mozambique | Maputo City, Maputo Province, Sofala and Nampula | To provide quantitative information on the job preferences of non-physician health professionals in Mozambique, examining how different aspects of jobs are valued and how health professionals might respond to policy options that would post them to district hospitals in rural areas. | Rural and underserved areas in Mozambique are characterized by lack of basic infrastructure (drinking water, sanitation facilities, electricity), limited road access during the rainy season, and difficulty attracting and retaining health workers. | Rural and underserved Lack of drinking water, sanitation facilities, and electricity Difficulty attracting and retaining health workers Limited road access during rainy season |
| Feasibility and acceptability of introducing malaria rapid diagnostic tests (MRDTS) and prereferral rectal artesunate (RA) into community case management in Mchinji, Malawi.^87^ | Journal article | Malawi | Mchinji district | This paper reports on findings from a pilot study whose aim was to assess the feasibility, acceptability and effects of integrating RDTs and rectal artesunate into the Malawi iCCM. | Hard-to-reach areas in Malawi are defined as areas that render difficulties in communication and access with the nearest health facilities. There are a variety of factors that come into play for an area to be described as hard-to-reach such as distance from nearest health facility of not less than 8 km, existence of a barrier such as a mountain between the village and the health facility, poor road that is inaccessible or difficult to access during the rainy seasons between the health facility and the village (area) | Difficulties in communication and access with the nearest health facilities Distance from nearest health facility of not less than 8 km Existence of a barrier such as a mountain between the village and the health facility Poor road that is inaccessible or difficult to access during the rainy season |
| Patient adherence to prescribed artemisinin-based combination therapy in Garissa County, Kenya, after three years of health care in a conflict setting.^88^ | Journal article | Kenya | Garissa | to measure current day patient adherence to the three-day AL treatment regimen and determine any changes in adherence since the 2010 study. | The climate is semi-arid with a range in temperature from 21Â°C to 39Â°C and an annual average bimodal rainfall. Malaria transmission is strongly seasonal; low during most of the year but epidemic prone during the rainy season. | Semi-arid climate Seasonal malaria transmission (epidemic prone during rainy season) |
| The impact of flooding on people living with HIV: a case study from the Ohangwena Region, Namibia.^89^ | Journal article | Namibia | Ohangwena region | This study aims at providing a deeper understanding of the impact that flooding has on people living with HIV (PLWHIV) as well as on HIV service providers in the Ohangwena region, Namibia. | Ohangwena is a predominantly rural region where people rely heavily on agriculture. It faces multiple challenges, including widespread poverty, high unemployment, poor access to clean water and sanitation, weak infrastructure, and limited access to health facilities and services. The region's location within the Cuvelai-Etosha and Okavango basins, along a floodplain, exposes it to seasonal flooding and drought due to its semi-arid climate with variable rainfall (480-600 mm annually). | Predominantly rural  Reliance on natural resources and subsistence farming Widespread poverty  High unemployment  Poor access to clean water and sanitation (during floods) Weak infrastructure  Limited access to health facilities and services Environmental vulnerability - located along a floodplain Semi-arid climate  Seasonal flooding and drought |
| Retention of health workers in rural Sierra Leone: findings from life histories.^90^ | Journal article | Sierra Leone | Bonthe, Koinadugu | To investigate the importance of different motivation factors in rural, including hard-to-reach, areas in Sierra Leone and thus to contribute to better decision financial and non-financial incentive packages, here and in similar context | Rural health workers face challenges, some of which stem from the difficult terrain (Bonthe-riverine; Koinadugu-mountainous), which add to common disadvantages of rural living poor social amenities, lack of electricity, and running water supply. | Difficult terrain (presence of rivers and mountains) Poor social amenities (dilapidated buildings -infested with rats) Lack of electricity and running water supply |
| Uptake of cervical cancer screening and associated factors among women in rural Uganda: A cross sectional study^91^ | Journal article | Uganda | Bugiri and Maguye districts, Eastern Uganda | To assess uptake of cervical cancer screening and associated factors among women in rural Uganda. | The largest burden of disease falls mostly on underserved populations in rural areas, where health care access is characterised by transport challenges, long distances to health centres, ill equipped health facilities and lack of information access. Being predominantly rural districts, most residents engage in subsistence farming, and a few operate small businesses in trading centres | Transport challenges (lack of transport and cost of transportation) Long distances to health centres,  Ill-equipped health facilities Lack of information access Reliance on subsistence farming and operation of small businesses |
| Polio eradication efforts in regions of geopolitical strife: The Boko Haram threat to efforts in sub-Saharan Africa.^92^ | Journal article | Cameroon and Nigeria | North Cameroon and Northern Nigeria | The aim of this paper is to present how Boko Haram activities may impact the plan to eliminate polio and to propose some solutions to avoid outbreaks of polio during and after armed conflicts | Cameroon and Nigeria have been countries affected by the terrorist and war activities of the Islamist sect Boko Haram. The effects of Boko Haram in these zones include violence against polio workers, disruption of immunization campaigns, and reduced access to healthcare and to immunization | Insurgency (Boko Haram) Terrorism War Reduced access to healthcare (disruption of immunization campaigns) |
| Rural Health Care Access and Policy in Developing Countries.^93^ | Journal article | SSA | SSA | To explores the situation in SSA in depth as an example of the limitations of rural health care access and policy in developing countries. | Rural will be defined according to the predominantly rural region classification within OECD’s regional typology: Predominantly rural regions have population densities of fewer than 150 people per square kilometre and more than 50% of the population lives in areas classified as rural communities | Low population density (150 people per square km) Majorly rural population (50%) |
| Health in a fragile state: A five-year review of mortality patterns and trends at Somalia's Banadir Hospital^94^ | Journal article | Somalia | Mogadishu | To explore morbidity and mortality patterns of the presenting conditions in the different units at the hospital such that an overview of Somalia can be inferred. | Mogadishu has been a haven for armed gangs, Islamic militants, and pirates, and for more than two decades, the country has been characterized by insecurity and outbreaks of hunger and inadequate access to basic needs and services such as sanitation facilities, safe water and healthcare. | Insecurity Hunger outbreaks Inadequate access to basic needs and services such as sanitation facilities, safe water and healthcare. |
| Improving Human Resources for Health means Retaining Health-Workers: Application of the WHO-Recommendations for the Retention of Health-Workers in Rural Northern-Nigeria.^95^ | Journal article | Nigeria | Katsina, Jigawa, and Zamfara | To describe the process used by three states in Northern Nigeria to adapt the WHO recommendations in order to establish state- specific incentive bundles to attract, recruit, and retain midwives in rural areas. | The dispersed rural settlements in Northern Nigeria lack desirable social amenities and infrastructure, and in some cases are very hard to reach | Dispersed settlements Lack of desirable social amenities and infrastructure (sanitation, electricity, telecom, schools and accomodation) Hard to reach |
| Access to health care for persons with disabilities in rural South Africa.^96^ | Journal article | South Africa | Rural Madwaleni | To explore issues of access to health care for persons with disabilities in an impoverished rural area in South Africa | According to Rowland & Lyons (1989, cited in Schur & Franco) [29] (p. 25), some key characteristics for rural areas are: 1) poorly developed and fragile health infrastructures. 2) high prevalence rates for chronic illness and disability. 3) socioeconomic hardships; and 4) physical barriers such as distance and availability of transportation, including a lack of public transportation  Madwaleni is a deeply rural and impoverished area defined by poor infrastructure, lack of basic service provision, low levels of literacy, high levels of unemployment, limited access to health care and education, high incidence of communicable diseases and high mortality rate | Poorly developed and fragile health infrastructures. High prevalence rates for chronic illness and disability High incidence of communicable diseases Deeply Rural Impoverished Socioeconomic hardships; low levels of literacy, high levels of unemployment Lack of basic service provision Physical barriers such as distance and availability of transportation, including a lack of public transportation |
| An exploratory study assessing psychological distress of indigents in Burkina Faso: a step forward in understanding mental health needs in West Africa.^97^ | Journal article | Burkina Faso | DiÃ©bougou, Gourcy, Kaya and Ouargaye | To assess mental health needs and psychological distress among the poorest in rural settings in Burkina Faso where food security and access to water, electricity, schooling, and healthcare are limited. | Rural settings in Burkina Faso where food security and access to water, electricity, schooling, and healthcare are limited. | Food insecurity  Limited access to water, electricity, schooling Limited access to healthcare |
| Is maternal education a social vaccine for childhood malaria infection? A cross-sectional study from war-torn Democratic Republic of Congo.^98^ | Journal article | Congo (DRC) | Butembo, Beni, and Goma(North Kivu) | To investigate whether maternal education acts as a social vaccine/modulator for childhood malaria infection. | The DRC is ranked among the least operationally and technically feasible countries for malaria elimination due to the intensity of malaria transmission, armed conflict, and political instability,gender-based violence, large-scale population displacement, and disruption of education for girls and women.Decimated civil and health care infrastructure, as well as social instability, after many years of civil and international conflict | Fragile state-political instability Decimated health care infrastructure-thus high malaria transmission Disrupted education for girls and women Socila instability - armed conflict,  High malaria transmission  Social instability Large scale population displacement |
| Family planning and the Samburu: A qualitative study exploring the thoughts of men on a population health and environment programme in rural Kenya.^99^ | Journal article | Kenya | Samburu | To investigates menâ€™s views on a PHE family planning (FP) programme delivered among the pastoral Samburu tribe in rural northern Kenya. | The Samburu region experiences frequent droughts, limiting water and food availability. The Samburu face several major barriers to accessing health services. The first is remoteness: villages included in this study were located between 15 and 50 km to the nearest health facility, and the Samburu travel across difficult terrain to access it. Some health services also cost money, and some families were too poor to afford them. | Drought Water and food insecurity Remote(villages are 15-50km from nearest health facility) Difficult terrain Limited healthcare access (due to distance, the services cost money and some families are too poor to afford them) |
| Factors influencing the decision of GHANAIAN optometry students to practice in rural areas after graduation.^100^ | Journal article | Ghana | Ghana | The aim of the study was to investigate factors that will influence Ghanaian Optometry studentsâ€™ decision to work in rural areas after completion of their training. | For the purpose of this study, a rural area was defined as an area with a population size cut off of less than 5000 with its inhabitants mainly engaged in primary (agricultural) activities. | Population size of less than 5000 Inhabitants mainly engaged in agricultural activities |
| Supporting healthcare professionals in a remote rural area of Tanzania.^101^ | Journal article | Tanzania | Rukwa-Sumbawanga | To explore how leadership coaching, educational consultancy, and professional support from UK colleagues could enhance job satisfaction among healthcare professionals in rural Tanzania, potentially improving staff recruitment and retention. | As one of the more remote, rural areas in Tanzania, Rukwa region has problems attracting and retaining healthcare professionals. This is partly because of its distance from big cities and universities. It is a 1 h flight from Dar es Salaam to Mbeya and then a dusty, 6 h bus journey to reach Sumbawanga. | Remote (distant from big cities and universities) |
| Implementing infection prevention and control capacity building strategies within the context of Ebola outbreak in a "Hard-to-Reach" area of Liberia.^102^ | Journal article | Liberia | Gbarpolu county | To highlight the combination strategies that were used to build local capacity and improve IPC practice in the health facilities and among the communities in Gbarpolu County. | Hard-to-reach areas are those places with geographical, physical,  communication, security, social, and economic barriers that make  them receive a level of public service that is relatively inequitable  and below the national benchmark.  The County has five health districts and six political districts and has dense forests, poor basic infrastructure, and a poor road network, with no paved roads and the majority of the roads, are tertiary roads or trails. Only 36% of the population lives within 5 kilometres of a health facility, which is the lowest in Liberia. Most villages and towns are separated by dense forests with track roads and wooden bridges the majority of which are in a grave state of disrepair. During the rainy season, many of the roads frequently become unpassable because of complete disconnection, destroyed bridges and sometimes by rising water levels. Some areas are encircled by rivers making access difficult during the rainy season. This terrain often discourages many partners and health care workers to work in the County. | Geographic isolation (dense forests, rivers, wooden bridges in grave state) Poor basic infrastructure  Poor road networks (no paved roads, majority of roads are tertiary roads or trails, impassable during the rainy season) Limited access to healthcare (distant health facilities > 5km away) |
| A model for predicting utilization of mHealth interventions in low-resource settings: case of maternal and newborn care in Kenya.^103^ | Journal article | Kenya | Kirirnyaga | To investigate the predictive power of TIPFit model | In low-resource settings, there are numerous socioeconomic challenges such as long distance to health facilities, poverty, inadequate health facilities, poor infrastructure, shortage of skilled health workers, illiteracy, limited budgets, and cultural barriers that contribute to high maternal and newborn deaths | Remote (long distance to health facilities) Poverty Poor infrastructure ( limited access to computers, power, and internet) Limited access to healthcare ( due to inadequate health facilities and shortage of skilled health workers) Illiteracy Limited budgets |
| Conduct of vaccination in hard-to-reach areas to address potential polio reservoir areas, 2014-2015.^104^ | Journal article | Nigeria | Borno, Bauchi, Kano, Yobe | To document the process of conducting integrated mobile vaccination in these hard-to-reach areas and the impact on immunization outcomes | The project operationally defined hard-to-reach areas as geographically difficult terrain, with any of the following criteria: having inter-ward,inter-Local Government Area (LGA), or interstate borders, scattered households, a nomadic population, or a waterlogged/riverine area, with no easy access to healthcare facilities and insecurity | Geographically difficult terrain with inter-ward, inter-Local Government Area (LGA), or interstate borders, scattered households, a nomadic population, or a waterlogged/riverine area, with  no easy access to healthcare facilities, insecurity |
| Satellite images and machine learning can identify remote communities to facilitate access to health services.^105^ | Journal article | Liberia | Rivercess County area | To determine whether a machine learning analyses of satellite imagery can be used to map remote communities to facilitate service delivery and planning. | For remote communities (defined as communities that are >5 km from the nearest health facility) | Remote (distant from health facility(>5km). Limited access to health services. |
| For more than money: willingness of health professionals to stay in remote Senegal.^106^ | Journal article | Senegal | Senegal | To explore the impact of contract type on rural retention and to estimate value of attributes in terms of willingness to stay (WTS) in current rural post | The Human Resources Department of the Senegalese Health Ministry has a working definition of difficult regions which includes geographical areas that constrain professional, personal and family growth and are characterised by a set of geographical, security, infrastructure and social service criteria | Geographical constraints Limited professional , personal and family growth Insecurity  Poor infrastructure (Housing) Insufficient social services |
| Door - to - door immunization strategy for improving access and utilization of immunization Services in Hard-to-Reach Areas: a case of Migori County, Kenya.^107^ | Journal article | Kenya | Migori | To determines the effect of an enhanced door-to-door immunization strategy on improving immunization coverage in hard-to-reach areas of Migori. | 43% of the population live below the poverty line. The main economic activities include agriculture, fishing, manufacturing, and small scale mining. Poor road network within the county limits access & utilization of health services especially during rainy seasons | Poverty (43% of the population is below the poverty line) Local economy is based on natural resources( agriculture, fishing, manufacturing, small-scale mining) Poor road network (limiting accessing during the rainy season) Limited access and utilization of health services (more pronounced during the rainy season) |
| Unawareness of health insurance expiration status among women of reproductive age in Northern Ghana: implications for achieving universal health coverage.^108^ | Journal article | Ghana | Upper East Region | To establish the relationship between low coverage of the NHIS and clientsâ€™ occasional unawareness of their membership's status | The UER environment is primarily arid savanna grassland with a climate comprised of an anuual May to September rainy season. About 70% of all UER residents are engaged in subsistence rain-fed agriculture. However, over-cropping and increasingly erratic rainfall have diminished agricultural productivity, exacerbating pervasive poverty and accelerating migration to the cities of southern Ghana. As a consequence of these circumstances, the UER ranks among Ghana's three most impoverished regions with a poverty prevalence of 55% and 40% of the population have no formal education | Arid Savanna grassland Low education levels (40% of the population have no formal education) Pervasive poverty-linked to reduced agricultural output Reliance on subsistence rain-fed agriculture Diminishing agricultural output (over-cropping and erratic rainfall) Rural-urban migration-linked to poverty-reduced agricultural output |
| Access to Functional Handwashing Facilities and Associated Factors among South Sudanese Refugees in Rhino Camp Settlement, Northwestern Uganda.^109^ | Journal article | Uganda | Rhino Camp-Northwestern Uganda | To assess access to functional handwashing facilities and associated factors among refugee households in Rhino settlement camp in Northwestern Uganda | Refugee settings are characterised by overcrowding and inadequate access to water and hygiene facilities which favour proliferation of faecal-oral diseases | Overcrowding Inadequate water and hygiene facilities-high prevalence of faecal-oral diseases |
| Emerging climate change-related public health challenges in Africa: A case study of the heat-health vulnerability of informal settlement residents in Dar es Salaam, Tanzania.^110^ | Journal Article | Tanzania | Dar es Salaam | To analyze the heat-health vulnerability of informal settlement residents in Dar es Salaam, Tanzania | These settlements are characterized by densely-packed housing, poor building materials, limited vegetation and lack of access to public services and amenities making their populations particularly vulnerable to heat. | Poor housing (densely packed housing, poor building materials, poorly ventilated) Limited vegetation Lack of access to public services and amenities (limited water access) Increased vulnerability to heat. |
| Respiratory health and eruptions of the Nyiragongo and Nyamulagira volcanoes in the Democratic Republic of Congo: A time-series analysis.^111^ | Journal article | Congo (DRC) | Nyiragongo and Nyamulagira near Goma,North Kivu | We investigated the possible spatiotemporal relationships between volcanic degassing represented by eruptive emissions of SO2 that occurred between 2000 and 2010, and the incidence of acute respiratory symptoms (ARS) in populations living in areas up to more than 100 km from the volcanoes | Nyiragongo and Nyamulagira, located in the Virunga National Park are among the most active African volcanoes and more than one million people living in the eastern Democratic Republic of Congo (DRC) are potentially exposed to their hazardous effects (suphur dioxide-leading to acute or chronic respiratory disorders). | Natural disaster-volcanic eruptions Exposure to hazardous effects (suphur dioxide-leading to acute or chronic respiratory disorders) |
| Access, demand, and utilization of childhood immunization services: A cross-sectional household survey in Western Area Urban district, Sierra Leone, 2019.^112^ | Journal article | Sierra Leone | WAU district | The objective was to provide actionable data to guide the Sierra Leone Ministry of Health and Sanitation (MoHS) to improve access, demand, and utilization of immunization services by tailoring and strengthening immunization services in Freetown and other similar urban settings in the country. | Slums were defined by the United Nations Development Program as having lack of basic services, sub-standard housing or illegal and inadequate building structures, overcrowding and high density, unhealthy living conditions and hazardous locations, insecure  tenure, irregular and informal settlements | Lack of basic services (safe water, sanitation facilities, rain water drainage, street lighting, electricity supply) Sub-standard housing or illegal and inadequate building structures Overcrowding (low space per person-5-12 persons sharing one bedroom unit)  High population density Unhealthy living conditions and hazardous locations(found on marginalised land-often along the seashore, on hillsides and basin of rivers) Insecure tenure, Irregular and informal settlements |
| Utilizing mobile health and community informants to collect real-time health care data in extremely low resource environments^113^ | Journal article | Malawi | Chitipa District -Northern region | The objective of this study was to test the efficacy and accuracy of data collected by community informants in extreme low-resource environments using electronic surveys and mobile phones | The Northern region is less densely populated, contains more mountainous terrain, and is home to a segment of the population with less access to education and employment. This region experiences frequent power outages (often in excess of 8 hours per day) and seasonal rains that make travel difficult and isolate areas within the region for months at a time.. Despite these geographic  challenges, inhabitants of the Northern Region of Malawi have limited but increasing access to cellular phones and data networks. | Low population density  Mountainous terrain Frequent power outages (often in excess of 8 hours per day) Limited access to education and employment Prolonged isolation (due to seasonal rains that make travel difficult) Limited access to internet (WiFi was unavailable in the most remote study areas and uploads were complete in a neardby area) |
| Mobile Device Usage by Gender Among High-Risk HIV Individuals in a Rural, Resource-Limited Setting^114^ | Journal article | South Africa | Masinga-KwaZulu-Natal | To characterize the usage of mobile technology among individuals undergoing community-based HIV testing while living in rural KwaZulu-Natal, South Africa to tailor mHealth interventions to engage PLWH. | Rural areas, often more impoverished, have limited broadband and energy coverage. Msinga is among the poorest subdistricts in the country, characterized by high unemployment (*85%), poor access to piped water (11%), and high HIV antenatal prevalence (30%) | Poverty  Limited broadband and energy coverage High unemployment Poor access to piped water High HIV antenatal prevalence |
| Universal WASH coverage; What it takes for fragile states. Case of Jariban district in Somalia.^115^ | Journal article | Somalia | Jariban district | To assess the types of water supply and sanitation technologies including behaviours underpin- ning WASH infrastructure development, access and use. | WASH access in fragile areas remains low,resulting in health-related risks, including diarrhoeal disease. Diarrhoeal diseases mostly affect children under five resulting in high mortality rates worldwide. Poor access also contributes to childhood undernutrition, maternal mortality neglected tropical diseases and respiratory infections | Political instability Limited access to clean water and sanitation facilities High prevalence of diarrhoeal diseases and mortality High mortality rates |
| Evaluating equity and coverage in mass drug administration for soil-transmitted helminth infections among school-age children in the hard-to-reach setting of southern Ethiopia.^116^ | Journal article | Ethiopia | South Omo Zone | This study thus aimed at assessing equity and coverage in MDA and identifying factors associated with drug coverage for STH infections among SAC in the hard-to-reach setting of southern Ethiopia | There are transportation problems in the entire district, and it is very far from regional and zone towns. The absence of roads for four-wheel vehicles and motorcycles makes for hard-to-reach data-collection sites. As such, the study area is defined as hard-to-reach setting | Distant from regional and zone towns Transportation problems -absence of roads for four-wheel vehicles and motorcycles. |
| Engineering environmental resilience: A matched cohort study of the community benefits of trailbridges in rural Rwanda.^117^ | Journal article | Rwanda | Rwanda | In this paper, we present a cohort study evaluating the potential community benefits of rural trailbridges - including economic, health and social outcomes for Rwandan communities experiencing environmental change. | Rural isolation can limit access to basic services and income-generating opportunities. Among some communities, rainfall induced flooding can cause increased uncertainty where first-mile transportation infrastructure is limited. In Rwanda, this challenge is apparent, where 90% of the population below the poverty line live in rural areas that are typically mountainous with frequent flooding - events that may be increasing in frequency and severity as the climate changes. | Mountainous terrain Frequent flooding High poverty levels (90% of the population are below the poverty line) |
| Beyond HIV prevalence: Identifying people living with HIV within underserved areas in South Africa.^118^ | Journal article | South Africa | KwaZulu-Natal province | To generate high-resolution, spatial predictions of HIV epidemiological measures for men and women (HIV) prevalence and density of PLHIV) in South Africa. | Underserved areas, defined as a greater than 30 min travel time to the nearest healthcare facility | Greater than 30 min travel to nearest health facility |
| Perspectives of local community leaders, health care workers, volunteers, policy makers and academia on climate change related health risks in mukuru informal settlement in nairobi, kenya-a qualitative study.^119^ | Journal article | Kenya | Mukuru | To explore knowledge and perspectives on climate change and health-related issues, with a particular focus on non-communicable diseases, in the informal settlement (urban slum) of Mukuru in Nairobi, Kenya | Mukuru's proximity to the Ngong River, its lower elevation than the average elevation in Nairobi and black clay soil, combined with many unpaved roads, narrow footpaths and poor waste management are all factors contributing to increased flood risk and frequent flooding in Mukuru. | Increased flood risk and fequent flooding Many unpaved roads and narrow footpaths Poor waste management |
| From Insecurity to Health Service Delivery: Pathways and System Response Strategies in the Eastern Democratic Republic of the Congo^120^ | Journal article | Congo (DRC) | Kivu | To enhance understanding of health system resilience strategies to inform health care providers at national and international levels designing response plans in similar conflict-affected settings | The provinces of North and South Kivu in eastern Congo (DRC) have experienced insecurity since the early 1990s. Despite a peace agreement in 2002 and elections in 2006, unrest continued with more than 140 armed groups in the region fighting for the control of natural resources and land leading to disruption of health services delivery. | Armed conflict |
| A contextual exploration of healthcare service use in urban slums in Nigeria.^121^ | Journal article | Nigeria | Oyo and Lagos state | To explore the patterns of healthcare utilization in these deprived neighborhoods by studying three such settlements in Nigeria | Migrant slum/community in Ibadan city of Oyo State: Structures are well-spaced, mostly permanent with variable energy-access, poor sanitation and refuse-filled drains. Indigenous slum in Ibadan city of Oyo States centrally located in the historical area of the city, along an old, tarred road. Mostly permanent but run-down structures, poor sanitation and refuse-filled drains characterize the space. The area is poorly planned with a minimal road network many health facilities are not easily accessible during emergencies. Cosmopolitan slum in Lagos State: Residents are mostly educated and employed, structures are mostly temporary, sanitation and basic services are limited, and the site has a higher crime rate than the others described above. | Run-down structures Variable energy access Poor sanitation and refuse-filled drains characterise the space. Poverty (household spending of less than $1.90 per day) Poorly planned area Minimal road network(few roads connecting key areas-poor accessibility) Limited sanitation and basic services High crime rate. |
| Sexual Assault, Pregnancy and HIV Infection among Young Girls in the Transkei Region of South Africa. Case Reports.^122^ | Journal article | South Africa | Transkei | To highlight the problem of sexual assault, pregnancy, and HIV infection among young girls in Transkei region of South Africa. | Transkei region of the Eastern Cape can best be described as deeply rural with bad roads, unclean water supply, limited electricity, few telephone connections, very limited access to transport and health services. Violence including sexual assaults | Deeply rural Bad roads (Paved roads with extensive defects requiring immediate rehabilitation or reconstruction. Unpaved roads needing reconstruction and major drainage works) Limited electricity  Few telephone connections  Unclean water supply  Very limited access to transport access and health services  Violence |
| Optimising scale and deployment of community health workers in Sierra Leone: A geospatial analysis.^123^ | Journal article | Sierra Leone | Sierra Leone | To inform implementation of the new CHW policy and  future MOHS planning. | The CHW policy 2021-2025 The MOHS-defined HTR areas as areas beyond 5 km from a health facility or between 3 and 5km of a health facility and in an area with difficult terrain. The MOHS did not define a difficult terrain. We defined a difficult terrain a as beyond 60min walking of a health facility.  In 2016-2020 policy the MOHS did not mention "difficult terrain". | Remote (distant to health facility (>5km)  OR  3-5km of a health facility with difficult terrain(beyond 60min walking of a health facility  Limited access to healthcare (due to difficult terrain and distance to health facility) |
| Economic evaluation of integrated services for non-communicable diseases and HIV: Costs and client outcomes in rural Malawi.^124^ | Journal article | Malawi | Neno District | To evaluate the costs and client outcomes associated with integrating screening and treatment for non-communicable diseases (NCDs) into HIV services in a  rural and remote part in southeastern Africa | Neno District is one of the most rural and impoverished districts in Malawi. Less than 5% of the population has electricity, and the average income is significantly below the country median. | Poor access to electricity (only 5% of the population has access) Low average income below the country median |
| Facilitators and barriers to implementation of integrated community case management of childhood illness: a qualitative case study of Kapiri Mposhi District.^125^ | Journal article | Zambia | Kapiri Mposhi District | To explore facilitators and barriers to implementation of ICCM in the health system in Kapiri Mposhi District, Zambia. | Rural/hard-to-reach are areas with limited socioeconomic facilities characterized by limited/absence of infrastructure such as roads, health facilities, piped water and electricity. Only 50% of the population in rural areas is within 5 km of a health facility. | Limited socioeconomic facilities  Limited/absence roads,health facilities, piped water and electricity Limited proximity to health facilities (Only 50% of the population in rural areas is within 5 km of a health facility) |
| Epidemiology of paediatric schistosomiasis in hard-to-reach areas and populations: a scoping review^126^ | Journal article | SSA | SSA | To document the epidemiology of pediatric schistosomiasis in hard-to- reach areas and populations, providing critical evidence on the need for targeted inclusion of this population when designing the expansion of preventive chemotherapy and schistosomiasis control activities. | We defined hard-to-reach areas and populations following Shaghaghi et al. [15], as (i) migrants/island and fishing communities/nomads, (ii) those living in remote physical and geographical locations, and (iii) those living in vulnerable social and economic situations such as minority groups, undocumented persons, socially excluded groups due to language and religious barriers. | Hard to reach -Remote physical/geographical locations |
| A social network analysis model approach to understand tuberculosis transmission in remote rural Madagascar.^127^ | Journal article | Madagascar | Androrangavola - Ifanadiana | To predict long-term TB burden within these communities with and without specific interventions. | The Androrangavola commune of the Ifanadiana district in southeastern Madagascar is an isolated mountainous region that exemplifies Madagascar’s rural demographics and living conditions including young age, large families living in shared households, and high community proximity. Access to healthcare facilities is limited because TB diagnosis occurs at a centralized facility serving approximately 200,000 people. | Limited access to healthcare Isolated (due to being mountainous) |
| Exploring Disability as a Determinant of Girl Child Marriage in Fragile States: A Multicountry Analysis.^128^ | Journal article | Mali, Uganda | Mali, Uganda | To assess the prevalence and associations of disability with GCM and IPV among currently married/cohabiting women (aged 20e24 years) in fragile states | Fragile states are countries characterized by poverty, conflict, political instability, insecurity, and natural disaster | Poverty, conflict, political instability, insecurity, and disaster |
| A community-based child health and parenting intervention to improve child HIV testing, health, and development in rural Lesotho (Early Morning Star): a cluster-randomised, controlled trial^129^ | Journal article | Lesotho | Mokhotlong district | To test the effectiveness of a community-based child health and parenting intervention to improve child HIV testing, health, and development in rural Lesotho | Villages are situated within a remote, mountainous terrain with poor transport and road facilities. | Remoteness Mountainous terrain Poor transport and road facilities |
| Empowerment Transformation Training Reduces Rape Among Girls and Young Women in South Sudan and the Kakuma Refugee Camp.^130^ | Journal article | South Sudan, Kenya | Kakuma refugee camp | To examine outcomes of Empowerment Transformation Training (ETT) (an adapted empowerment self-defense program; empowerment self-defense) among female participants in South Sudan and the Kakuma refugee camp | Rape occurs at high rates in South Sudan and Kakuma refugee camps, a region characterized by armed conflict, gender inequity, and economic crisis. To date, we know little about how to prevent rape in this region of the world. | Armed conflict Economic crisis Gender inequity |
| Socio-ecological impacts of extreme weather events in two informal settlements in Nairobi, Kenya.^131^ | Journal article | Kenya | Mathare and Kibera | This study aims to explore the multidimensional impacts of EWEs on the daily lives of women in informal settlements through the lens of socio-ecological theory | In Kenya, specifically, the government has described informal settlements as human settlement[s] characterized by dilapidated housing structures (structures made of galvanized iron sheets, mud, wood, and concrete built very close to one another and narrow, unpaved paths between them with little vegetation. all factors that can exacerbate ecological sensitivity such as ambient temperatures), overcrowding, abject poverty and unemployment, high insecurity incidences, insecure land tenure, exclusion of physical development, inadequate infrastructural services and often located in an unsustainable environment | Poor housing(dilapidated structures made of galvanized iron sheets, mud, wood and concrete built close to one another, narrow unpaved paths between them with little vegetation)-high ecological sensitivity Overcrowding High ecological sensitivity Abject poverty and unemployment High insecurity incidences Insecure land tenure, Exclusion of physical development Inadequate infrastructural services (water sanitation and electricity Located in an unsustainable environment |
| Effect of seasonal variability on the increased malaria positivity rate in drought-prone malaria endemic areas of Ethiopia.^132^ | Journal article | Ethiopia | Oromia | To assess the impact of seasonal and climate variability on the burden of malaria in one of drought-prone areas of north central Ethiopia | The study site was selected due to its drought prone nature, and almost all districts were designated as malarious | Drought prone High prevalence of malaria |
| Assessing the health consequences of northern Ethiopian armed conflict, 2022.^133^ | Journal article | Ethiopia | Nothern Ethiopia | To assess the impact of the northern Ethiopian armed conflict on population health | The conflicts in Northern Ethiopia have had a substantial impact on the communities health, both directly through conflict-related causalities and indirectly through the disintegration of the health system and health-supporting structures, which has severely hampered the supply and utilization of health services. | Conflict and political instability Disintegration of the health system and health-supporting structures |
| COVID-19 vaccination campaigns in fragile and conflict-affected settings, Somalia.^134^ | Report | Somalia | Somalia | - | Somalia is a fragile and conflict- affected country. the prolonged humanitarian crisis has resulted in a weakened fragmented and under-funded health system | Fragility and conflict Humanitarian crisis Weakened health system Under-funded health system |
| The consideration of public petition No. 57 of 2023 regardsing the gazettement of Nyatike West, Nyatike North and Nyatike South as hardship areas.^135^ | Report | Kenya | Nyatike West, Nyatike North and Nyatike South | The consideration of public petition No. 57 of 2023 regardsing the gazettement of Nyatike West, Nyatike North and Nyatike South as hardship areas | Non/limited availability and accessibility of food Non availability /limited access to portable water Non/inadequate transport and communication services Non/limited availability of social services and amenities Harsh climate and terrain ( area dries with erratic rainfall, area gets flooding during rainy season, region is hot and dry, region is semi-arid, difficult terrain-mountainous with deep gullies). Security and high possibility of security threat ( human-wildlife conflict, banditry/cattle rustling) Poverty index | Non/limited availability and accessibility of food Non availability /limited access to portable water Non/inadequate transport and communication services Non/limited availability of social services and amenities Harsh climate (Area dries with erratic rainfall, area floods during rainy season, region is hot and dry, region is semi-arid) Terrain (Difficult terrain-mountainous with deep gullies) Insecurity, Poverty |
| **Tuesday, 30 January 2018** Parliament met at 2.24 p.m. in Parliament House, Kampala. ^136^ | Report | Uganda | Uganda | To present a statement on the hard-to-reach framework on the Floor of Parliament | By definition the hard to reach or stay is a remote or insecure area that is unable to attract and retain sufficient numbers of motivated staff because of difficult living conditions. The hard to reach/ stay areas are called hard to work in areas. | Lack of transport facilities (incl. poor road network)  Lack of accommodation  Lack of social amenities (bank, grid electricity, education facilities)  Insecurity and hostility of locals  Others: (Poor climate, poor terrain, national parks and water bodies) |
| Rural Health Fact Sheet 2015^137^ | Report | South Africa | South Africa | To provide a summary of information on the state of rural health. | There are a number of factors that could be included in the definition:  • Population density: rural populations tend to have lower population densities than urban populations and are often more dispersed  • Demographic factors: fertility, age, sex, morbidity and mortality profiles of communities often differ between rural and urban settings • Economic factors: rural populations tend to be poorer Introduction and have less access to economic opportunities than their urban counterparts. Economic activity in rural areas is based around labour on farms and mines, subsistence farming and informal sectors  • Settlement patterns: rural settlements tend to consist of small towns, villages and dispersed homesteads  • Land use: rural land tends to be used for agriculture, tourism and industry such as forestry and mining while urban land is used for manufacturing, commerce, and retail  • Access to services: rural communities globally tend to have less access to basic services such as healthcare, education, water and sanitation  • Migration patterns and linkages: rural (and urban) populations are not static. People migrate from and to rural areas. These migration patterns are often circular with people leaving at particular times (e.g. for employment) and returning at others (e.g. to retire or when they become ill). | Lower population density Impoverished Less access to economic opportunities Small town towns, villages, and dispersed homesteads Less access to basic services such as healthcare, education, water and sanitation |

References

1. Duboz P, Macia E, Diallo AH, Cohen E, Bergouignan A, Seck SM. The good life in rural and urban Senegal: A qualitative and quantitative study. PLoS One. 2021;16(5):e0252134.

2. Shikalepo EE. Characteristics of rural areas and their effects on teaching and learning dynamics. International Journal of Social Sciences and Management Review. 2019;2(4):20–36.

3. Harmse A. Node selection for the integrated sustainable rural development programme in South Africa. Development Southern Africa. 2010;27(3):429–45.

4. Equity in public services in Tanzania and Uganda: policy note (English). Washington D.C.: The WorldBank.; 2011. Report No.: 56511-AFR.

5. The European Commission. OECD Regional Development Studies Applying the Degree of Urbanisation: A Methodological Manual to Define Cities, Towns and Rural Areas for International Comparisons: OECD Publishing; 2021.

6. FAO. Guidelines on Defining Rural Areas and Compiling Indicators for Development Policy. 2018;

7. Kamere IM, Makatiani MI, Nzau AK. Policy interventions for attraction and retention of female teachers in rural secondary schools: Perspectives of rural educators in Makueni County, Kenya. Msingi Journal. 2019;1(2):50–9.

8. Nkya O. Multisectoral criteria for defining underserved areas: A basis for developing an incentive package. Dar es Salaam: Tanzania Human Resource Capacity Project, Intra-Health International; 2012.

9. WHO. WHO guideline on health workforce development, attraction, recruitment and retention in rural and remote areas. World Health Organization; 2021.

10. World Health Organization. Increasing access to health workers in remote and rural areas through improved retention: global policy recommendations. 2010 [cited 2024 Dec 12];71. Available from: https://iris.who.int/handle/10665/44369

11. Strasser R, Strasser S. Reimaging Primary Health Care Workforce in Rural and Underserved Settings [Internet]. World Bank; 2020 [cited 2024 Dec 12]. Available from: https://hdl.handle.net/10986/34906

12. Ansah EK, Narh-Bana S, Affran-Bonful H, Bart-Plange C, Cundill B, Gyapong M, et al. The impact of providing rapid diagnostic malaria tests on fever management in the private retail sector in Ghana: A cluster randomized trial. BMJ (Online). 2015;350.

13. Vergunst R, Swartz L, Mji G, MacLachlan M, Mannan H. “You must carry your wheelchair” - barriers to accessing healthcare in a South African rural area. Global Health Action. 2015;8(1).

14. Owusu-Ansah FE, Tagbor H, Togbe MA. Access to health in city slum dwellers: The case of Sodom and Gomorrah in Accra, Ghana. Afr J Prim Health Care Fam Med. 2016;8(1).

15. Musoke R, Chimbaru A, Jambai A, Njuguna C, Kayita J, Bunn J, et al. A Public Health Response to a Mudslide in Freetown, Sierra Leone, 2017: Lessons Learnt. Disaster Med Public Health Preparedness. 2019;14(2):256–64.

16. Erismann S, Gürler S, Wieland V, Prytherch H, Künzli N, Utzinger J, et al. Addressing fragility through community-based health programmes: Insights from two qualitative case study evaluations in South Sudan and Haiti. Health Res Policy Syst. 2019;17(1).

17. Lequechane JD, Mahumane A, Chale F, Nhabomba C, Salomão C, Lameira C, et al. Mozambique’s response to cyclone Idai: How collaboration and surveillance with water, sanitation and hygiene (WASH) interventions were used to control a cholera epidemic. Infect Dis Pover. 2020;9(1).

18. Arscott-Mills T, Kebaabetswe P, Tawana G, Mbuka DO, Makgabana-Dintwa O, Sebina K, et al. Rural Exposure During Medical Education And Student Preference For Future Practice Location - A Case Of Botswana. Afr J Prim Health Care Fam Med. 2021;8(1).

19. Misanya D, Øyhus AO. The role of community-based knowledge and local institutions in managing landslides on the slopes of Mount Elgon, Uganda. International Journal of Emergency Management. 2015;11(2):89–104.

20. Ani-Amponsah M, Richter S. Midwives’ Experiences of Rural Maternal - Newborn Care in Ghana: A Phenomenological Inquiry. Online Journal of Rural Nursing & Health Care. 2021;21(2):84–116.

21. Galley W, Anthony BP. Beyond Crop-Raiding: Unravelling the Broader Impacts of Human-Wildlife Conflict on Rural Communities. Environmental Management [Internet]. 2024;74(3):590–608. Available from: https://search.ebscohost.com/login.aspx?direct=true&AuthType=ip,shib&db=cin20&AN=178878193&site=ehost-live&authtype=ip,uid

22. Gebregziabher G, Holden S. Does irrigation enhance and food deficits discourage fertilizer adoption in a risky environment? Evidence from Tigray, Ethiopia. Journal of Development and Agricultural Economics [Internet]. 2011;3(10):514–28. Available from: http://www.academicjournals.org/JDAE/abstracts/abstracts/abstracts2011/Sept/26%20September/Gebregziabher%20and%20Holden.htm

23. Cherotich VK, Saidu O, Bebe BO. Access to climate change information and support services by the vulnerable groups in semi-arid Kenya for adaptive capacity development. African Crop Science Journal. 2012;20(Suppl. 2):169–80.

24. Stockton G. Sugar for the tea: assistance and the state of pastoralism in the Horn of Africa. Pastoralism: Research, Policy and Practice. 2012;2:6.

25. Kolstad JR. How does additional education affect willingness to work in rural remote areas in low-income contexts? An application on health workers in Tanzania. Journal of Development Studies. 2013;49(2):301–14.

26. Saina CK, Cheserek GJ, Owino JO, Murgor FA. Traditional coping strategies to famine among the Keiyo people living in Kerio Valley, Kenya. Journal of Emerging Trends in Economics and Management Sciences. 2013;4(2):163–9.

27. Abbas HB, Routray JK. Vulnerability to flood-induced public health risks in Sudan. Disaster Prevention & Management [Internet]. 2014;23(4):395–419. Available from: http://www.emeraldinsight.com/info/journals/dpm/dpm.jsp

28. Ayantunde AA, Turner MD, Kalilou A. Participatory analysis of vulnerability to drought in three agro-pastoral communities in the West African Sahel. Pastoralism: Research, Policy and Practice. 2015;5(13).

29. Harison K, Mark B, Imwatis A. Spatial variability of malnutrition and predictions based on climate change and other causal factors: a case study of North Rift ASAL counties of Kenya. Journal of Earth Science & Climatic Change. 2017;8(10):1000416.

30. Sewell SJ, Desai SA, Mutsaa E, Lottering RT. A comparative study of community perceptions regarding the role of roads as a poverty alleviation strategy in rural areas. Journal of Rural Studies. 2019;71:73–84.

31. Cooper S, Hutchings P, Butterworth J, Solome Joseph SJ, Abinet Kebede AK, Parker A, et al. Environmental associated emotional distress and the dangers of climate change for pastoralist mental health. Global Environmental Change. 2019;59:101994.

32. Berhane A, Hadgu G, Worku W, Abrha B. Trends in extreme temperature and rainfall indices in the semi-arid areas of Western Tigray, Ethiopia. Environmental Systems Research. 2020;9(3).

33. Eneh OC. Abuja slums: development, causes, waste-related health challenges, government response and way-forward. Environment, Development and Sustainability. 2020;23(6):9379–96.

34. Aliu IR, Akoteyon IS, Soladoye O. Living on the margins: socio-spatial characterization of residential and water deprivations in Lagos informal settlements, Nigeria. Habitat International. 2021;107.

35. Longe OM. An assessment of the energy poverty and gender nexus towards clean energy adoption in rural South Africa. Energies [Internet]. 2021;14(12). Available from: https://www.mdpi.com/1996-1073/14/12/3708

36. Manyangadze T, Mavhura E, Mudavanhu C, Pedzisai E. An exploratory analysis of the spatial variation of malaria cases and associated household socio-economic factors in flood-prone areas of Mbire district, Zimbabwe. GeoJournal. 2021;87(6):4439–54.

37. Opoku MP, Jiya AN, Kanyinji RC, Nketsia W. Retention and job satisfaction among rural primary school teachers in Malawi. Rural Society. 2022;31(2):101–14.

38. Govoetchan R, Osse R, Sovi A, Akogbeto M. Four years trends of malaria admissions in rural and urban Kandi health facilities in northeast of Benin Republic. International Journal of Mosquito Research. 2022;9(6 Part B):156–62.

39. Mkhize X, Mthembu BE, Napier C. Transforming a local food system to address food and nutrition insecurity in an urban informal settlement area: a study in Umlazi township in Durban, South Africa. Journal of Agriculture and Food Research. 2023;12.

40. Omam LA, Metuge A. Rapid response mechanism in conflict-affected settings of Cameroon: lessons learned from a multisector intervention for internally displaced persons. Journal of Global Health Reports. 2023;7(e2023057).

41. Fonkwo J, Besong M, Ngong J, Glazier A, Talawa D, Alio A. The effect of the anglophone crisis on youth sexual and reproductive health in the northwest region of Cameroon: a qualitative study. Journal of Global Health Reports. 2023;7(e2023040).

42. Gwenzi J, Mafongoya PL, Mashonjowa E. Effects of Climate Change on Food Production in Semi-Arid Areas: A Case Study of Uzumba Maramba Pfungwe District, Zimbabwe. In: Leal Filho W, Djekic I, Smetana S, Kovaleva M, editors. Handbook of Climate Change Across the Food Supply Chain. Cham: Springer International Publishing; 2022. p. 451–65.

43. Joshua MK, Ngongondo C, Chipungu F, Monjerezi M, Liwenga E, Majule AE, et al. Climate change in semi-arid Malawi: Perceptions, adaptation strategies and water governance. Jamba (Potchefstroom, South Africa). 2016;8(3):255.

44. Maes P, Harries AD, Van den Bergh R, Noor A, Snow RW, Tayler-Smith K, et al. Can timely vector control interventions triggered by atypical environmental conditions prevent malaria epidemics? A case-study from Wajir County, Kenya. PloS one. 2014;9(4):e92386.

45. Molelekwa GF, Mukhola MS, Van der Bruggen B, Luis P. Preliminary studies on membrane filtration for the production of potable water: a case of Tshaanda rural village in South Africa. PloS one. 2014;9(8):e105057.

46. Kamugisha SR, Dobson AE, Stewart AG, Haven N, Mutahunga B, Wilkinson E. A Retrospective Cross Sectional Study of the Effectiveness of a Project in Improving Infant Health in Bwindi, South Western Uganda. Frontiers in public health. 2018;6(101616579):290.

47. Simba DO, Kakoko D, Nyamhanga T, Mrango Z, Mujinja P. Improving prompt access to malaria diagnostics and treatment in rural remote areas using financial benefit for community health workers in Kilosa district, Tanzania. Research and reports in tropical medicine. 2018;9(101562656):137–46.

48. Akola J, Binala J, Ochwo J. Guiding developments in flood-prone areas: Challenges and opportunities in Dire Dawa city, Ethiopia. Jamba (Potchefstroom, South Africa). 2019;11(3):704.

49. Coldiron ME, Von Seidlein L, Grais RF. Seasonal malaria chemoprevention: successes and missed opportunities. Malaria journal. 2017;16(1):481.

50. Pieterse P. Citizen feedback in a fragile setting: social accountability interventions in the primary healthcare sector in Sierra Leone. Disasters. 2019;43 Suppl 2(7702072, b4i):S132–50.

51. Hakami L, Castle PM, Kiernan J, Choi K, Rahantamalala A, Rakotomalala E, et al. Epidemiology of soil transmitted helminth and Strongyloides stercoralis infections in remote rural villages of Ranomafana National Park, Madagascar. Pathogens and global health. 2019;113(2):94–100.

52. Kuradusenge M, Kumaran S, Zennaro M. Rainfall-Induced Landslide Prediction Using Machine Learning Models: The Case of Ngororero District, Rwanda. International journal of environmental research and public health. 2020;17(11).

53. Inman EN, Hobbs RJ, Tsvuura Z. No safety net in the face of climate change: The case of pastoralists in Kunene Region, Namibia. PloS one. 2020;15(9):e0238982.

54. Brear MR, Hammarberg K, Fisher J. Community participation in health research: an ethnography from rural Swaziland. Health promotion international. 2020;35(1):e59–69.

55. Belayneh M, Loha E, Lindtjorn B. Seasonal Variation of Household Food Insecurity and Household Dietary Diversity on Wasting and Stunting among Young Children in A Drought Prone Area in South Ethiopia: A Cohort Study. Ecology of food and nutrition. 2021;60(1):44–69.

56. Belayneh M, Loha E, Lindtjorn B. Spatial Variation of Child Stunting and Maternal Malnutrition after Controlling for Known Risk Factors in a Drought-Prone Rural Community in Southern Ethiopia. Annals of global health. 2021;87(1):85.

57. Zhang T, Qi X, He Q, Hee J, Takesue R, Yan Y, et al. The Effects of Conflicts and Self-Reported Insecurity on Maternal Healthcare Utilisation and Children Health Outcomes in the Democratic Republic of Congo (DRC). Healthcare (Basel, Switzerland). 2021;9(7).

58. Bonetti S, Sutanudjaja EH, Mabhaudhi T, Slotow R, Dalin C. Climate change impacts on water sustainability of South African crop production. Environmental research letters : ERL [Web site]. 2022;17(8):084017.

59. Dafallah A, Elmahi OKO, Ibrahim ME, Elsheikh RE, Blanchet K. Destruction, disruption and disaster: Sudan’s health system amidst armed conflict. Conflict and health. 2023;17(1):43.

60. McNeilly H, Thielecke M, Mutebi F, Banalyaki M, Reichert F, Wiese S, et al. Tungiasis Stigma and Control Practices in a Hyperendemic Region in Northeastern Uganda. Tropical medicine and infectious disease. 2023;8(4).

61. Kabeta WF, Tamiru M, Tsige D, Ware H. An integrated geotechnical and geophysical investigation of landslide in Chira town, Ethiopia. Heliyon. 2023;9(7):e17620.

62. Mthombeni S, Coopoo Y, Noorbhai H. Factors promoting and hindering sporting success among South African former Olympians from historically disadvantaged areas. South African journal of sports medicine. 2023;35(1):v35i1a15068.

63. Itimi K, Dienye PO, Ordinioha B. Community participation and childhood immunization coverage: A comparative study of rural and urban communities of Bayelsa State, south-south Nigeria. Nigerian medical journal. 2012;53(1):21–5.

64. Onyeagoziri OJ, Shaw C, Ryan T. A system dynamics approach for understanding community resilience to disaster risk. Jamba (Potchefstroom, South Africa). 2021;13(1):1037.

65. Harris B, Ajisola M, Alam RM, Watkins JA, Arvanitis TN, Bakibinga P, et al. Mobile consulting as an option for delivering healthcare services in low-resource settings in low- and middle-income countries: A mixed-methods study. Digital health. 2021;7(101690863):20552076211033425.

66. Ford L, Bethancourt HJ, Swanson Z, Nzunza R, Wutich A, Brewis A, et al. Water Insecurity, Water Borrowing, and Psychosocial Stress Among Daasanach Pastoralists in Northern Kenya. Water international. 2023;48(1):63–86.

67. Ndabezitha KE, Mubangizi BC, John SF. Adaptive capacity to reduce disaster risks in informal settlements. Jamba (Potchefstroom, South Africa). 2024;16(1):1488.

68. Mohamed A, Homeida A. Hunger in the shadow of conflict: analyzing malnutrition and humanitarian challenges in Sudan. Conflict and health. 2024;18(1):50.

69. Fransen S, Werntges A, Hunns A, Sirenko M, Comes T. Refugee settlements are highly exposed to extreme weather conditions. Proceedings of the National Academy of Sciences of the United States of America. 2023;120(0):e2206189120.

70. Gele AA, Bjune GA. Armed conflicts have an impact on the spread of tuberculosis: the case of the Somali Regional State of Ethiopia. Conflict and health. 2010;4(101286573):1.

71. Kekana HN, Ruhiiga TM, Ndou NN, Palamuleni LG. Environmental justice in South Africa: the dilemma of informal settlement residents. GeoJournal. 2023;(101084445):1–17.

72. Mauch V, Weil D, Munim A, Boillot F, Coninx R, Huseynova S, et al. Structure and management of tuberculosis control programs in fragile states--Afghanistan, DR Congo, Haiti, Somalia. Health policy (Amsterdam, Netherlands). 2010;96(2):118–27.

73. Majumdar B, Mazaleni N. The experiences of people living with HIV/AIDS and of their direct informal caregivers in a resource-poor setting. Journal of the International AIDS Society. 2010;13(1):20.

74. Awofeso N. Improving health workforce recruitment and retention in rural and remote regions of Nigeria. Rural and remote Health. 2010;10(1):162–71.

75. Blackwell PJ. East Africa’s pastoralist emergency: Is climate change the straw that breaks the camel’s back? Third World Quarterly. 2010;31(8):1321–38.

76. Agyei-Baffour P, Kotha SR, Johnson JC, Gyakobo M, Asabir K, Kwansah J, et al. Willingness to work in rural areas and the role of intrinsic versus extrinsic professional motivations-a survey of medical students in Ghana. BMC Medical Education. 2011;11(1):56.

77. Ewing VL, Lalloo DG, Phiri KS, Roca-Feltrer A, Mangham L. J, Sanjoaquin M. A. Seasonal and geographic differences in treatment-seeking and household cost of febrile illness among children in Malawi. Malaria Journal. 2011;10:32.

78. Kisia J, Nelima F, Otieno DO, Kiilu K, Emmanuel W, Sohani S, et al. Factors associated with utilization of community health workers in improving access to malaria treatment among children in Kenya. Malaria Journal. 2012;11:1–7.

79. Munga MA, Torsvik G, Maestad O. Using incentives to attract nurses to remote areas of Tanzania: a contingent valuation study. Health policy and planning. 2014;29(2):227–36.

80. Alabi O, Doctor HV, Afenyadu GY, Findley SE. Lessons learned from setting up the Nahuche Health and Demographic Surveillance System in the resource-constrained context of northern Nigeria. Global health action. 2014;7(1):23368.

81. Mpofu R, Daniels PS, Adonis TA, Karuguti WM. Impact of an interprofessional education program on developing skilled graduates well-equipped to practise in rural and underserved areas. Rural and Remote Health. 2014;14(3):84–94.

82. Ojakaa D, Olango S, Jarvis J. Factors affecting motivation and retention of primary health care workers in three disparate regions in Kenya. Human resources for health. 2014;12:1–13.

83. Mduma ER, Gratz J, Patil C, Matson K, Dakay M, Liu S, et al. The etiology, risk factors, and interactions of enteric infections and malnutrition and the consequences for child health and development study (MAL-ED): description of the Tanzanian site. Clinical infectious diseases : an official publication of the Infectious Diseases Society of America. 2014;59 Suppl 4(a4j, 9203213):S325-30.

84. Bakibinga P, Ettarh R, Ziraba AK, Kyobutungi C, Kamande E, Ngomi N, et al. The effect of enhanced public–private partnerships on Maternal, Newborn and child Health Services and outcomes in Nairobi–Kenya: the PAMANECH quasi-experimental research protocol. BMJ open. 2014;4(10):e006608.

85. Uzondu CA, Doctor HV, Findley SE, Afenyadu GY, Ager A. Female health workers at the doorstep: a pilot of community-based maternal, newborn, and child health service delivery in northern Nigeria. Global Health: Science and Practice. 2015;3(1):97–108.

86. Honda A, Vio F. Incentives for non-physician health professionals to work in the rural and remote areas of Mozambique—a discrete choice experiment for eliciting job preferences. Human resources for health. 2015;13:1–12.

87. Phiri T, Kaunda B, Bauleni A, Melody D, George J, Chimuna T, et al. Feasibility and acceptability of introducing malaria rapid diagnostic tests (MRDTS) and pre-referral rectal artesunate (RA) into community case management in Mchinji, Malawi. American Journal of Tropical Medicine and Hygiene. 2015;93(4):255–255.

88. Gore-Langton GR, Alenwi N, Mungai J, Erupe NI, Eves K, Kimwana FN, et al. Patient adherence to prescribed artemisinin-based combination therapy in Garissa County, Kenya, after three years of health care in a conflict setting. Malaria journal. 2015;14:1–10.

89. Anthonj C, Nkongolo O. T, Schmitz P, Hango J. N, Kistemann T. The impact of flooding on people living with HIV: a case study from the Ohangwena Region, Namibia. Global health action. 2015;8((Anthonj) Institute for Hygiene and Public Health, WHO CC for Health Promoting Water Management and Risk Communication, University of Bonn, Bonn, Germany;(Nkongolo, Hango) Directorate of Special Programmes, Ministry of Health and Social Services (Ohangwen):26441.

90. Wurie HR, Samai M, Witter S. Retention of health workers in rural Sierra Leone: findings from life histories. Human resources for health. 2016;14:1–15.

91. Ndejjo R, Mukama T, Musabyimana A, Musoke D. Uptake of cervical cancer screening and associated factors among women in rural Uganda: a cross sectional study. PloS one. 2016;11(2):e0149696.

92. Bigna J.J.R. Polio eradication efforts in regions of geopolitical strife: The Boko Haram threat to efforts in sub-Saharan Africa. African Health Sciences. 2016;16(2):584–7.

93. Strasser R, Kam SM, Regalado SM. Rural health care access and policy in developing countries. Annual review of public health. 2016;37(1):395–412.

94. Kulane A, Sematimba D, Mohamed LM, Ali AH, Lu X. Health in a fragile state: a five-year review of mortality patterns and trends at Somalia’s Banadir Hospital. International journal of general medicine. 2016;303–10.

95. Afenyadu G.Y., Adegoke A.A., Findley S. Improving Human Resources for Health means Retaining Health-Workers: Application of the WHO-Recommendations for the Retention of Health-Workers in Rural Northern-Nigeria. Journal of health care for the poor and underserved. 2017;28(3):1066–86.

96. Vergunst R., Swartz L., Hem K.-G., Eide A.H., Mannan H., MacLachlan M., et al. Access to health care for persons with disabilities in rural South Africa. BMC health services research. 2017;17(1):741.

97. Pigeon-Gagne E, Hassan G, Yaogo M, Ridde V. An exploratory study assessing psychological distress of indigents in Burkina Faso: a step forward in understanding mental health needs in West Africa. International journal for equity in health. 2017;16(1):143.

98. Ma C, Claude K. M, Kibendelwa Z. T, Brooks H, Zheng X, Hawkes M. Is maternal education a social vaccine for childhood malaria infection? A cross-sectional study from war-torn Democratic Republic of Congo. Pathogens and Global Health [Internet]. 2017;111(2):98–106. Available from: http://www.tandfonline.com/loi/ypgh#.VwHiPU1f1Qs

99. Kock L, Prost A. Family planning and the Samburu: A qualitative study exploring the thoughts of men on a population health and environment programme in rural Kenya. International Journal of Environmental Research and Public Health. 2017;14(5):528.

100. Boadi-Kusi S. B, Kyei S, Okyere V. B, Abu S. L. Factors influencing the decision of GHANAIAN optometry students to practice in rural areas after graduation. BMC medical education. 2018;18(1):188.

101. Berney S, Halpern H. Supporting healthcare professionals in a remote rural area of Tanzania. London Journal of Primary Care. 2018;10(4):89–92.

102. Oji MO, Haile M, Baller A, Trembley N, Mahmoud N, Gasasira A, et al. Implementing infection prevention and control capacity building strategies within the context of Ebola outbreak in a" Hard-to-Reach" area of Liberia. Pan African Medical Journal. 2018;31(1).

103. Mburu S, Oboko R. A model for predicting utilization of mHealth interventions in low-resource settings: case of maternal and newborn care in Kenya. BMC medical informatics and decision making. 2018;18(1):67.

104. Bawa S, Shuaib F, Saidu M, Ningi A, Abdullahi S, Abba B, et al. Conduct of vaccination in hard-to-reach areas to address potential polio reservoir areas, 2014–2015. BMC Public Health. 2018;18:113–20.

105. Bruzelius E, Le M, Kenny A, Downey J, Danieletto M, Baum A, et al. Satellite images and machine learning can identify remote communities to facilitate access to health services. Journal of the American Medical Informatics Association [Internet]. 2019;26(8–9):806–12. Available from: http://jamia.oxfordjournals.org/content/22/e1

106. Honda A, Krucien N, Ryan M, Diouf I. S.N, Salla M, Nagai M, et al. For more than money: willingness of health professionals to stay in remote Senegal. Human resources for health. 2019;17(1):28.

107. Shikuku DN, Muganda M, Amunga SO, Obwanda EO, Muga A, Matete T, et al. Door–to–door immunization strategy for improving access and utilization of immunization Services in Hard-to-Reach Areas: a case of Migori County, Kenya. BMC Public Health. 2019;19:1–11.

108. Kanmiki E.W., Bawah A.A., Akazili J., Agorinyah I., Awoonor-Williams J.K., Phillips J.F., et al. Unawareness of health insurance expiration status among women of reproductive age in Northern Ghana: implications for achieving universal health coverage. Journal of health, population, and nutrition. 2019;38(1):34.

109. Namara F, Mendoza H, Tumukunde G, Wafula S. T. Access to Functional Handwashing Facilities and Associated Factors among South Sudanese Refugees in Rhino Camp Settlement, Northwestern Uganda. Journal of Environmental and Public Health [Internet]. 2020;2020((Namara) Department of Disaster Risk Management, Uganda Red Cross Society, Kampala, Uganda(Namara) Faculty of Health Science, Uganda Martyrs University, Kampala, Uganda(Mendoza, Tumukunde, Wafula) Department of Disease Control and Environmental Health, Sc):3089063. Available from: http://www.hindawi.com/journals/jeph/

110. Pasquini L, van Aardenne L, Godsmark C. N, Lee J, Jack C. Emerging climate change-related public health challenges in Africa: A case study of the heat-health vulnerability of informal settlement residents in Dar es Salaam, Tanzania. Science of the Total Environment [Internet]. 2020;747((Pasquini, van Aardenne, Lee, Jack) Climate System Analysis Group, University of Cape Town, Private Bag X3, Rondebosch 7701, South Africa(Pasquini) African Climate and Development Initiative, University of Cape Town, Private Bag X3, Rondebosch 7701, South):141355. Available from: https://www.elsevier.com/locate/scitotenv

111. Michellier C, Katoto P. D.M.C, Dramaix M, Nemery B, Kervyn F. Respiratory health and eruptions of the Nyiragongo and Nyamulagira volcanoes in the Democratic Republic of Congo: A time-series analysis. Environmental Health: A Global Access Science Source [Internet]. 2020;19(1):62. Available from: http://www.ehjournal.net/home/

112. Feldstein LR, Sutton R, Jalloh MF, Parmley L, Lahuerta M, Akinjeji A, et al. Access, demand, and utilization of childhood immunization services: A cross-sectional household survey in Western Area Urban district, Sierra Leone, 2019. Journal of global health. 2020;10(1):010420.

113. Ebner PJ, Friedricks NM, Chilenga L, Bandawe T, Tolomiczenko G, Alswang JM, et al. Utilizing mobile health and community informants to collect real-time health care data in extremely low resource environments. Journal of Global Health. 2020;10(2):020411.

114. Campbell BR, Choi K, Neils MG, Canan C, Moll A, Dillingham R, et al. Mobile Device Usage by Gender Among High-Risk HIV Individuals in a Rural, Resource-Limited Setting. Telemedicine and e-Health. 2021 June 1;27(6):615–24.

115. Mafuta W, Zuwarimwe J, Mwale M. Universal WASH coverage; What it takes for fragile states. Case of Jariban district in Somalia. PLoS ONE [Internet]. 2021;16(2 February):e0247417. Available from: https://journals.plos.org/plosone/article/file?id=10.1371/journal.pone.0247417&type=printable

116. Asfaw MA, Hailu C, Beyene TJ. Evaluating equity and coverage in mass drug administration for soil-transmitted helminth infections among school-age children in the hard-to-reach setting of Southern Ethiopia. Pediatric health, medicine and therapeutics. 2021;325–33.

117. Thomas E, Bradshaw A, Mugabo L, MacDonald L, Brooks W, Dickinson K, et al. Engineering environmental resilience: A matched cohort study of the community benefits of trailbridges in rural Rwanda. Science of the Total Environment [Internet]. 2021;771((Thomas, Bradshaw, MacDonald) Mortenson Center in Global Engineering, University of Colorado Boulder, Boulder, CO 80303, United States(Mugabo) Amazi Yego Ltd, Kigali, Rwanda(Brooks) Department of Economics, Arizona State University, Tempe, AZ 85281, Unite):145275. Available from: https://www.elsevier.com/locate/scitotenv

118. Kim H, Tanser F, Tomita A, Vandormael A, Cuadros DF. Beyond HIV prevalence: identifying people living with HIV within underserved areas in South Africa. BMJ global health. 2021;6(4):e004089.

119. Andersen J. G, Karekezi C, Ali Z, Yonga G, Kallestrup P, Kraef C. Perspectives of local community leaders, health care workers, volunteers, policy makers and academia on climate change related health risks in mukuru informal settlement in nairobi, kenya-a qualitative study. International Journal of Environmental Research and Public Health [Internet]. 2021;18(22):12241. Available from: https://www.mdpi.com/1660-4601/18/22/12241/pdf

120. Altare C, Castelgrande V, Tosha M, Malembaka E. B, Spiegel P. From Insecurity to Health Service Delivery: Pathways and System Response Strategies in the Eastern Democratic Republic of the Congo. Global health, science and practice. 2021;9(4):915–27.

121. Fayehun O, Ajisola M, Uthman O, Oyebode O, Oladejo A, Owoaje E, et al. A contextual exploration of healthcare service use in urban slums in Nigeria. Plos one. 2022;17(2):e0264725.

122. Meel B. Sexual Assault, Pregnancy and HIV Infection among Young Girls in the Transkei Region of South Africa. Case Reports. Indian Journal of Forensic Medicine and Toxicology. 2022;16(1):1503–6.

123. Oliphant N. P, Ray N, Curtis A, Musa E, Sesay M, Kandeh J, et al. Optimising scale and deployment of community health workers in Sierra Leone: A geospatial analysis. BMJ Global Health [Internet]. 2022;7(5):e008141. Available from: https://gh.bmj.com/

124. Wroe E. B, Mailosi B, Price N, Kachimanga C, Shah A, Kalanga N, et al. Economic evaluation of integrated services for non-communicable diseases and HIV: Costs and client outcomes in rural Malawi. BMJ Open [Internet]. 2022;12(11):e063701. Available from: http://bmjopen.bmj.com/content/early/by/section

125. Riri J. V, Silumbwe A, Mweemba C, Zulu J. M. Facilitators and barriers to implementation of integrated community case management of childhood illness: a qualitative case study of Kapiri Mposhi District. BMC health services research. 2022;22(1):497.

126. Isaiah PM, Sólveig Palmeirim M, Steinmann P. Epidemiology of pediatric schistosomiasis in hard-to-reach areas and populations: a scoping review. Infect Dis Poverty. 2023 Apr 17;12(1):37.

127. Pando C, Hazel A, Tsang L. Y, Razafindrina K, Andriamiadanarivo A, Rabetombosoa R. M, et al. A social network analysis model approach to understand tuberculosis transmission in remote rural Madagascar. BMC public health. 2023;23(1):1511.

128. Kanselaar S, Zhang C, Grace K. T, Lindley L. L, Zaidi J, Gupta J. Exploring Disability as a Determinant of Girl Child Marriage in Fragile States: A Multicountry Analysis. Journal of Adolescent Health [Internet]. 2023;73(6):1101–9. Available from: https://www.elsevier.com/locate/jadohea

129. Tomlinson M, Marlow M, Stewart J, Makhetha M, Sekotlo T, Mohale S, et al. A community-based child health and parenting intervention to improve child HIV testing, health, and development in rural Lesotho (Early Morning Star): a cluster-randomised, controlled trial. The Lancet HIV. 2024;11(1):e42–51.

130. Wheeler LA, Edwards KM, Omondi B, Kaeke B, Ndirangu M, Sinclair J, et al. Empowerment transformation training reduces rape among girls and young women in South Sudan and the Kakuma Refugee Camp. Journal of Adolescent Health. 2024;74(4):820–7.

131. Balakrishnan AK, Otieno S, Dzombo M, Plaxico L, Ukoh E, Obara LM, et al. Socio-ecological impacts of extreme weather events in two informal settlements in Nairobi, Kenya. Front Public Health. 2024;12:1389054.

132. Tefera S, Bekele T, Ketema T. Effect of seasonal variability on the increased malaria positivity rate in drought-prone malaria endemic areas of Ethiopia. Journal of Parasitic Diseases. 2024;1–12.

133. Arage MW, Kumsa H, Asfaw MS, Kassaw AT, Mebratu E, Tunta A, et al. Assessing the health consequences of northern Ethiopian armed conflict, 2022. Journal of public health policy. 2024;45(1):43.

134. Farid M., Ibrahim A., Mohammad H., Hassan Q., Omar M.A., Ismael M.A., et al. COVID-19 vaccination campaigns in fragile and conflict-affected settings, Somalia. Bulletin of the World Health Organization. 2024;102(9):674–80.

135. Report on the Consideration of Public Petition No. 57 of 2023 regarding the Gazettement of Nyakite West, Nyatike North and Nyatike South as Hardship Areas. Parliament of the Republic of Kenya; 2023.

136. Ministerial statement on the implementation of the Hard-to-Reach Framework in the Uganda Public Service. Parliament of the Republic of Uganda; 2018.

137. RURAL HEALTH FACT SHEET 2015 [Internet]. [cited 2025 July 10]. Available from: https://rhap.org.za/wp-content/uploads/2022/11/2015-Rural-Health-Fact-Sheet-1.pdf#page=92.17
