# Supplementary material for: Conceptualising hardship areas in Sub-Saharan Africa: a scoping review
Source: Int J Equity Health. 2025 Nov 21;24:326. doi: 10.1186/s12939-025-02694-x (PMC12639685; doi:10.1186/s12939-025-02694-x)
Supplement: Supplementary file 6 — Supplementary Material 6: File name: Additional file 6. File format: Doc (Microsoft word). Title of data: Supplementary Table 4: Number of studies conducted in specific countries and regions. Description: Table summarizing the distribution of included studies by country and region within SSA, highlighting areas with high or low representation [file 12939_2025_2694_MOESM6_ESM.docx]

**Supplementary Table 4*:* Number of studies conducted in specific countries and regions**

| **Region of the study** |  | **Number of studies** |
| --- | --- | --- |
| Specific Countries | Kenya **(KEN)** | 17 |
|  | Uganda **(UGA)** | 7 |
|  | South Africa (**ZAF)** | 18 |
|  | Tanzania **(TZA)** | 7 |
|  | Rwanda **(RWA)** | 2 |
|  | Congo DRC **(COD)** | 4 |
|  | Ethiopia **(ETH)** | 11 |
|  | Sudan **(SDN)** | 3 |
|  | South Sudan **(SSD)** | 1 |
|  | Zambia **(ZMB)** | 1 |
|  | Namibia **(NAM)** | 3 |
|  | Mozambique **(MOZ)** | 2 |
|  | Zimbabwe **(ZWE)** | 2 |
|  | Malawi **(MWI)** | 6 |
|  | Madagascar **(MDG)** | 2 |
|  | Lesotho **(LSO)** | 1 |
|  | Swaziland **(SWZ)** | 1 |
|  | Botswana **(BWA)** | 1 |
|  | Somalia **(SOM)** | 3 |
|  | Liberia **(LBR)** | 2 |
|  | Cameroon **(CMR)** | 2 |
|  | Burkina Faso **(BFA)** | 1 |
|  | Ghana **(GHA)** | 7 |
|  | Niger **(NER)** | 1 |
|  | Nigeria **(NGA)** | 9 |
|  | Senegal **(SEN)** | 2 |
|  | Mali **(MLI)** | 1 |
|  | Sierra Leone **(SLE)** | 5 |
|  | Benin **(BEN)** | 1 |
| Multi-country | Sahel | 1 |
|  | Horn of Africa | 1 |
|  | East and West Africa | 1 |
|  | East Africa | 3 |
|  | West Africa | 1 |
|  | Sub-Saharan Africa | 2 |
|  | LMICs | 1 |
| Global |  | 4 |
