# Supplementary material for: Conceptualising hardship areas in Sub-Saharan Africa: a scoping review
Source: Int J Equity Health. 2025 Nov 21;24:326. doi: 10.1186/s12939-025-02694-x (PMC12639685; doi:10.1186/s12939-025-02694-x)
Supplement: Supplementary file 7 — Supplementary Material 7: File name: Additional file 7. File format: Doc (Microsoft word). Title of data: Supplementary Figure 2: Word cloud showing the features of hardship areas in Sub-Saharan Africa. Description: Visual representation of the frequently mentioned features of hardship areas in SSA, generated from the extracted data to highlight commonly occurring concepts [file 12939_2025_2694_MOESM7_ESM.docx]

**Supplementary Figure 2: Word cloud showing the features of hardship areas in Sub-Saharan Africa**

**
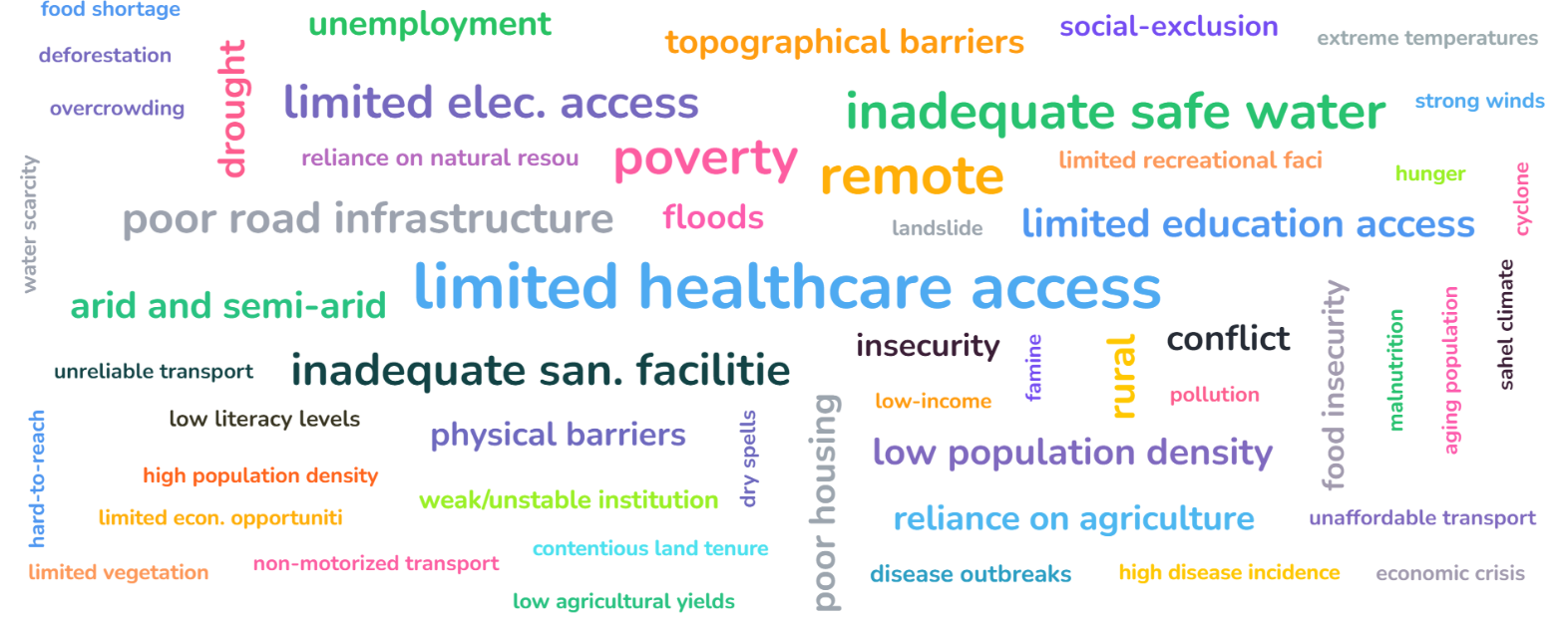
**

Commonly reported features of hardship areas across literature. The variation in font size represents the frequency of citation: the bigger the font size, the more frequent the feature was cited in reviewed literature.
